# Comparative Efficacy and Safety of Weekly GLP-1/GIP Agonists vs. Weekly Insulin in Type 2 Diabetes: A Network Meta-Analysis of Randomized Controlled Trials

**DOI:** 10.3390/biomedicines12091943

**Published:** 2024-08-23

**Authors:** Hazem Ayesh, Sajida Suhail, Suhail Ayesh, Kevin Niswender

**Affiliations:** 1 Deaconess Health System, Evansville, IN 47708, USA; 2 Gene Medical Labs, Gaza 00972, Palestine; sajidasuhail91@gmail.com (S.S.); profayesh@gmail.com (S.A.); 3 Department of Medicine, Division of Diabetes, Endocrinology, and Metabolism, Vanderbilt University Medical Center, Nashville, TN 37232, USA; kevin.niswender@vumc.org

**Keywords:** diabetes management, once-weekly insulin, GLP-1/GIP receptor agonists, HbA1c reduction, treatment tolerability

## Abstract

Background: Diabetes mellitus (DM) significantly impacts global health due to its complications and the economic burden it places on healthcare systems. The rise of novel once-weekly diabetes medications with different mechanisms of action necessitates an evaluation of their relative efficacy and safety. Objectives: This study compares the efficacy and tolerability of once-weekly insulin analogs (icodec and BIF) with once-weekly GLP-1/GIP agonists (semaglutide, exenatide, tirzepatide, dulaglutide) in managing type 2 diabetes mellitus (T2DM). Methods: We conducted a network meta-analysis (NMA) using data from randomized controlled trials (RCTs) that compared these treatments with a baseline of daily basal insulin. Primary outcomes included changes in HbA1c, body weight, and tolerability. Results: The analysis integrated data from 25 RCTs, involving 18,257 patients. Tirzepatide significantly outperformed other treatments in reducing HbA1c and promoting weight loss. Weekly insulins, compared to GLP-1/GIP agonists, showed a more tolerable profile and were beneficial for certain patient demographics emphasizing weight stability. Conclusion: Our findings suggest that while once-weekly GLP-1/GIP agonists provide superior glycemic control and weight management, weekly insulins offer viable options for patients prioritizing fewer side effects and weight stability. This comprehensive comparison aids in refining personalized treatment strategies for T2DM management.

## 1. Introduction

Diabetes mellitus (DM) imposes a substantial global burden, contributing significantly to morbidity and mortality rates, particularly due to associated complications such as cardiovascular disease, renal dysfunction, and neurological impairments [1,2]. Moreover, the economic impact of diabetes on healthcare systems is considerable [2]. Hence, optimizing diabetes management is imperative, especially given the escalating prevalence of this metabolic disorder.

There are remarkable advancements in the practice of diabetes, particularly in the realm of once-weekly agents designed to enhance treatment adherence and simplify patient management [3,4]. However, the proliferation of these agents has created a complex decision-making landscape for healthcare providers, necessitating evidence-based strategies for personalized treatment plans [4].

Among the recent advancements are glucagon-like peptide-1 (GLP-1) agonists and glucose-dependent insulinotropic peptide (GIP) dual agonists and once-weekly insulin, which offer promising avenues for diabetes management [5,6]. These novel agents offer intriguing alternatives to traditional therapies, potentially revolutionizing the treatment paradigm. Nonetheless, the challenge lies in personalizing therapy in order to optimize treatment outcomes while minimizing adverse effects and the risk of polypharmacy.

Our study aims to address this challenge by comparing the efficacy, safety, and tolerability profiles of once-weekly insulin formulations, such as icodec and BIF, against once-weekly GLP-1/GIP agonists, including semaglutide, exenatide, tirzepatide, and dulaglutide. Specifically, we will evaluate their impact on glycated hemoglobin (HbA1c) levels, body weight, and tolerability, providing valuable insights into their relative efficacy and safety profiles.

Conducting a comprehensive comparison through network meta-analysis (NMA) represents a significant endeavor with far-reaching implications. NMA enables the integration of data from diverse randomized controlled trials (RCTs), offering a robust framework for assessing the comparative efficacy and safety of different treatment modalities. By elucidating the relative merits of once-weekly GLP-1/GIP agonists and insulin formulations, our analysis aims to inform clinical decision-making, guide guideline development, and inform healthcare policies. Ultimately, this endeavor seeks to enhance the delivery of personalized and effective care for individuals living with type 2 diabetes mellitus (T2DM).

## 2. Methods

The protocol for this network meta-analysis was registered in OSF registries (https://osf.io/p7szu, accessed on 30 May 2024) [7]. Results were reported according to preferred reporting items for systematic reviews and meta-analyses (PRISMA) guidelines [8].

### 2.1. Data Sources

We conducted a comprehensive search in the databases Scopus, PubMed, Cochrane, and Web of Science from inception to 5 April 2024 (search strategy detailed in Supplement S1).

### 2.2. Study Selection

We included randomized controlled trials (RCTs) involving individuals diagnosed with type 2 diabetes mellitus that evaluated weekly GLP-1/GIP agonists and weekly insulin in comparison to daily insulin. The primary outcome measures evaluated were glycemic control (HbA1c levels and fasting plasma glucose), weight change, and safety parameters (incidence of hypoglycemia and adverse events). We excluded non-randomized studies, observational studies, extension studies, exploratory analyses, post hoc analyses, and animal studies. Additionally, we excluded studies involving individuals without type 2 diabetes mellitus, pediatric populations, studies that did not have daily insulin as a comparator, and studies not reporting relevant outcomes related to efficacy and safety.

### 2.3. Data Extraction

The primary efficacy outcome extracted from each RCT was the mean change from baseline in hemoglobin A1c (HbA1c). Secondary efficacy outcomes included mean changes from baseline in fasting plasma glucose (FPG) and body weight (BW). Safety outcomes encompassed the incidence of hypoglycemia, treatment-emergent adverse events (TEAE), and rates of treatment discontinuation due to adverse events.

In our analysis, we calculated the standard error (SE) of the mean with the equation: SE = (CI_width)/(2 × z_value), where CI_width denotes the breadth of the confidence interval and z_value is the z-score corresponding to the targeted confidence level [9]. This approach enabled us to estimate the SE when only the confidence interval (CI) and the mean of the data were provided [9]. We selected data for analysis that represented the longest follow-up periods and included comparisons with daily basal insulin since there is no direct comparison between GLP-1/GIP and weekly insulin. In cases where the original studies reported data as medians and ranges, we converted these to means and standard deviations to ensure uniformity in our analysis [10]. 

### 2.4. Risk of Bias Assessment

The risk of bias was assessed using the RoB2 tool [11]. This evaluation covered several domains, deviations from intended interventions, randomization process, missing outcome data, outcome measurement, and selection of the reported results.

### 2.5. Statistical Analysis

We performed frequentist random effects network meta-analysis that compared multiple treatments by analyzing data from various studies, allowing for both direct and indirect comparisons. This method synthesizes evidence to determine the relative efficacy of each treatment, even if some treatments were not directly compared in any individual study [12]. 

For continuous outcomes (HbA1c, FPG, BW), we calculated mean differences (MDs) with 95% confidence intervals (CIs). For binary outcomes (TEAE, hypoglycemic events, incidence of serious adverse events, and treatment discontinuation due to adverse events), we calculated risk ratios (RRs) with 95% CIs.

Pairwise comparisons were conducted for direct comparisons [13]. We validated the transitivity assumption by utilizing node splitting to compare direct and indirect outcomes, ensuring their consistency [14]. Heterogeneity was evaluated by comparing the magnitude of the common between-study variance (τ2) for each outcome with empirical distributions of heterogeneity variances [15]. We also created surface under the cumulative ranking curve (SUCRA) graphs for efficacy outcomes and conducted meta-regression analyses for age, BMI, and diabetes duration [16]. SUCRA provides a hierarchical ranking of treatments facilitating comparison across studies [16]. We conducted a sensitivity analysis to ensure the robustness of our results by applying a Bayesian network meta-analysis model with meta-regression (accounting for age, BMI, and duration) for efficacy outcomes, thereby confirming the findings of our network meta-analysis.

We performed the analysis using RStudio version number 4.3.2 (31 October 2023) and R packages: netmeta version number 2.9-0, gemtc version number 1.0-2, and rjags version number 4.15 [17,18,19,20]. The certainty of evidence was evaluated using the confidence in network meta-analysis (CINeMA) framework and online application (https://cinema.ispm.unibe.ch/, accessed on 30 May 2024) [21]. 

## 3. Results

### 3.1. Study Characteristics

**Figure 1:**
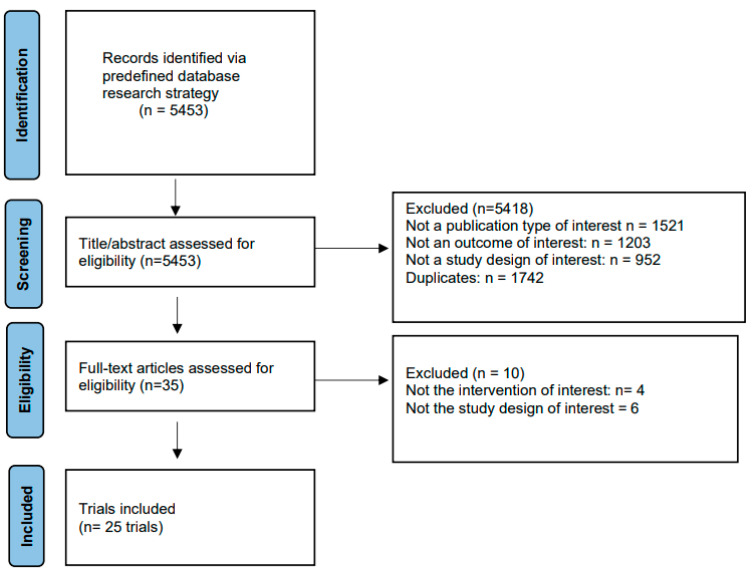
PRISMA flowchart for study selection.

**Figure 2:**
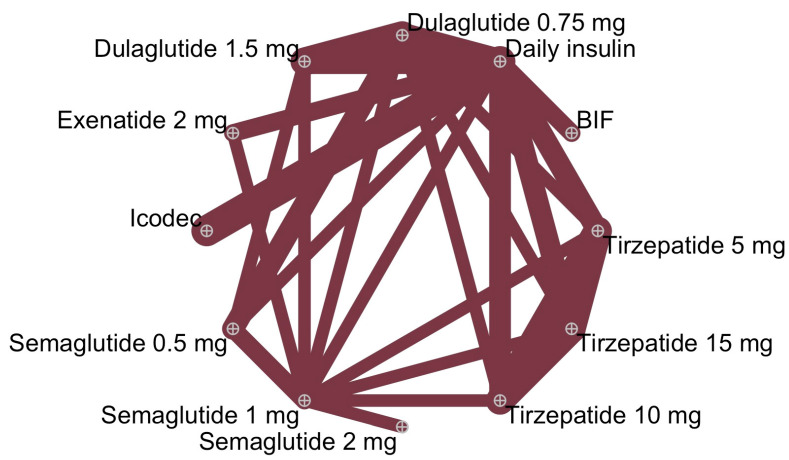
Meta-analysis networks for change in HbA1c level. Each circle indicates a treatment node. Lines connecting two nodes represent direct comparisons between two treatments; the thickness of the lines is proportional to the number of trials directly comparing the two connected treatments.

A total of 25 trials [22,23,24,25,26,27,28,29,30,31,32,33,34,35,36,37,38,39,40,41,42,43,44,45,46] were included in the analysis (Figure 1). The network of trials comparing HbA1c is shown in Figure 2. The characteristics of studies and patients’ baseline features are presented in Supplement S2.

The mean age of participants in the included studies was 57.8 years (standard deviation [SD]: 11.2), with 57.8% being male. The mean body mass index (BMI) was 31.2 kg/m^2^ (SD: 5.6). The mean HbA1c level was 8.3% (SD: 0.8), and the mean duration of diabetes was 11.0 years (SD: 7.7).

There was substantial heterogeneity in HbA1c, fasting plasma glucose (FPG), body weight (BW), risk of adverse events, and risk of hypoglycemia. Overall, global inconsistency was minimal. The primary outcome’s risk of bias was low in 20 trials, with 5 trials presenting some concerns. Comparison-adjusted funnel plots indicated no publication bias for HbA1c, hypoglycemia, FPG, and body weight, but there was some indication of publication bias for the incidence of adverse events. (Supplement S4). The certainty of evidence was generally moderate to high for each of the main comparisons, with some comparisons having low certainty. All data from certainty analysis are included in (Supplement S6).

### 3.2. HbA1c

**Figure 3:**
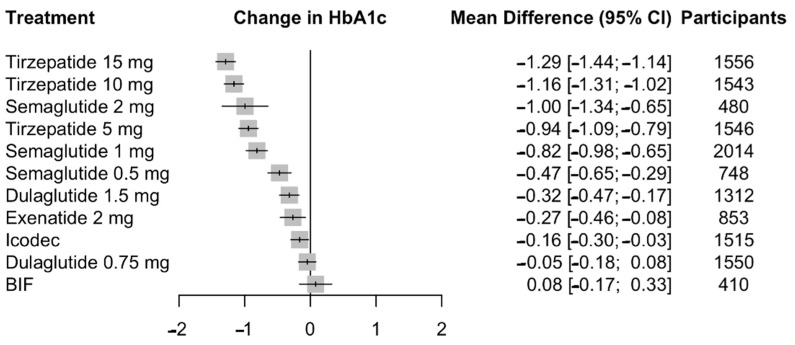
Network meta-analysis results for change from baseline. HbA1c compared with daily insulin. Effect sizes are presented as mean difference (MD) and 95% confidence intervals (CI). Treatments are presented according to their effect estimate compared with glargine. Abbreviations: HbA1c: hemoglobin A1c, BIF: basal insulin Fc.

**Figure 4:**
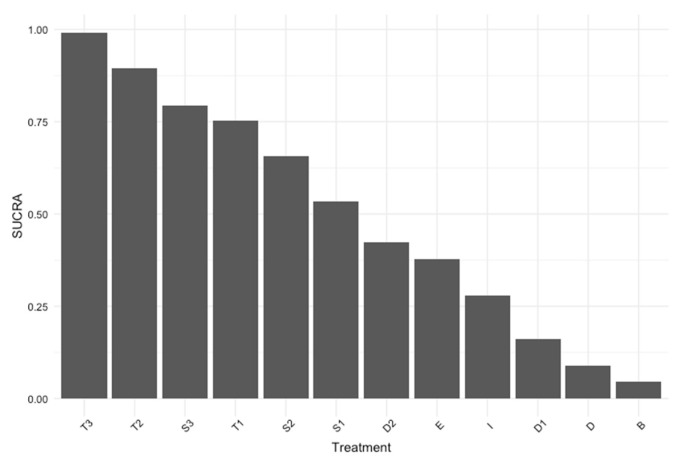
The surface under the cumulative ranking (SUCRA) plot displays the ranking probabilities of treatments in reducing hemoglobin A1c (HbA1c). Higher SUCRA scores indicate a greater likelihood of lowering HbA1c. Abbreviations: T3: tirzepatide 15 mg, T2: tirzepatide 10 mg, T1: tirzepatide 5 mg, S3: semaglutide 2 mg, S2: semaglutide 1 mg, S1: semaglutide 0.5 mg, D2: dulaglutide 1.5 mg, E: exenatide 2 mg, I: icodec, D: daily insulin, B: BIF, D1: dulaglutide 0.75 mg.

We evaluated the effects of GLP-1/GIP receptor agonists and weekly insulin analogs on HbA1c reduction, using daily insulin as the active comparator. The analysis encompassed 25 studies involving 18,257 patients. Tirzepatide showed the highest efficacy with the 15 mg dose achieving a mean difference (MD) in HbA1c reduction of −1.29 (95% CI: −1.44 to −1.14), followed by the 10 mg dose with an MD of −1.16 (95% CI: −1.31 to −1.02), and the 5 mg dose with an MD of −0.94 (95% CI: −1.09 to −0.79). Semaglutide showed substantial HbA1c reductions across dosages: the 2 mg dose had an MD of −1.00 (95% CI: −1.34 to −0.65), the 1 mg dose at −0.82 (95% CI: −0.98 to −0.65), and the 0.5 mg dose at −0.47 (95% CI: −0.65 to −0.29). Exenatide 2 mg also contributed with an MD of −0.27 (95% CI: −0.46 to −0.08). Among the weekly insulin analogs, icodec demonstrated a modest but significant reduction in HbA1c compared to daily insulin, with an MD of −0.16 (95% CI: −0.30 to −0.02). BIF, however, did not demonstrate a statistically significant change, with an MD of 0.08 (95% CI: −0.17 to 0.33). Detailed results of pairwise comparisons can be found in Supplement S5, and network meta-analysis results are presented in Figure 3. The SUCRA ranking is depicted in Figure 4, and confirms tirzepatide 15 mg as the top-ranked treatment for HbA1c reduction, unaffected by patient age, BMI, or diabetes duration according to our meta-regression analyses. Meta-regression results are depicted in Supplement S7.

### 3.3. FPG

**Figure 5:**
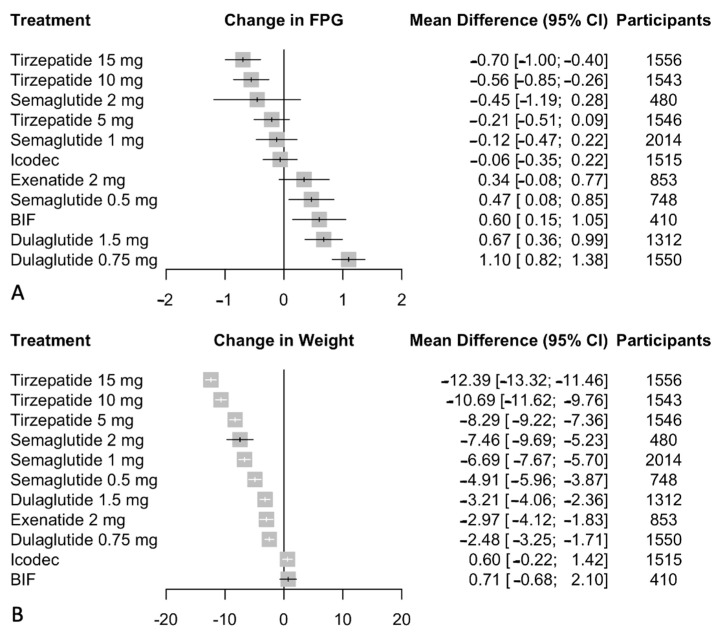
(**A**) Network meta-analysis results for change from baseline in FPG compared with daily insulin. (**B**). Network meta-analysis results for change from baseline in weight compared with daily insulin. Effect sizes are presented as mean difference (MD) and 95% confidence intervals (CI). Treatments are presented according to their effect estimate compared with daily insulin. Abbreviations: FPG: fasting plasma glucose, BIF: basal insulin Fc.

Our analysis focused on the impact of GLP-1/GIP receptor agonists and weekly insulin analogs on fasting plasma glucose (FPG) levels, using daily insulin as the baseline for comparison. The pooled data from 18,257 patients reveal that tirzepatide at 15 mg had the most substantial effect on reducing FPG, with a mean difference (MD) of −0.70 mmol/L (95% CI: −1.00 to −0.41), and the 10 mg dose showed a significant decrease with an MD of −0.55 mmol/L (95% CI: −0.84 to −0.25). The 5 mg dose of tirzepatide also suggested a reduction in FPG levels, with an MD of −0.21 mmol/L (95% CI: −0.50 to 0.09), although this was not statistically significant. Semaglutide displayed varied effects across dosages: the 2 mg dose led to a reduction with an MD of −0.45 mmol/L (95% CI: −1.19 to 0.28), the 1 mg dose had a non-significant impact with an MD of −0.12 mmol/L (95% CI: −0.47 to 0.22), and the 0.5 mg dose increased FPG levels slightly with an MD of 0.47 mmol/L (95% CI: 0.09 to 0.85). Exenatide 2 mg had an MD of 0.34 mmol/L (95% CI: −0.08 to 0.76), indicating a possible, but not statistically significant, increase. BIF and icodec had MDs of 0.60 mmol/L (95% CI: 0.15 to 1.05) and −0.06 mmol/L (95% CI: −0.35 to 0.22), respectively, reflecting a significant increase for BIF and a non-significant difference for icodec. Detailed results of pairwise comparisons can be found in Supplement S5. Network meta-analysis results are presented in Figure 5. Meta-regression results are depicted in Supplement S7.

### 3.4. BW

We evaluated the effects of GLP-1/GIP receptor agonists and weekly insulin analogs on weight loss, using daily insulin as the active comparator. We pooled data from 25 studies encompassing 18,257 patients. Tirzepatide showed the most notable weight loss, with the 15 mg dose achieving a mean difference (MD) of −12.39 kg (95% CI: −13.32 to −11.46), and the 10 mg and 5 mg doses showing MDs of −10.69 kg (95% CI: −11.62 to −9.76) and −8.29 kg (95% CI: −9.22 to −7.36), respectively. Significant reductions in weight were also seen with semaglutide, with the 2 mg dose resulting in an MD of −7.46 kg (95% CI: −9.69 to −5.23), the 1 mg dose at −6.69 kg (95% CI: −7.67 to −5.70), and the 0.5 mg dose at −4.91 kg (95% CI: −5.96 to −3.87). Dulaglutide at 1.5 mg and 0.75 mg also showed substantial weight reductions, with MDs of −3.21 kg (95% CI: −4.06 to −2.36) and −2.48 kg (95% CI: −3.25 to −1.71), respectively. Exenatide 2 mg contributed to weight loss with an MD of −2.97 kg (95% CI: −4.12 to −1.83). In contrast, icodec showed a non-significant trend toward weight gain with an MD of 0.60 kg (95% CI: −0.22 to 1.42). BIF suggested a potential increase in weight, with an MD of 0.71 kg (95% CI: −0.68 to 2.10), although this was not statistically significant. Detailed results of pairwise comparisons can be found in Supplement S5. Network meta-analysis results are presented in Figure 5. Meta-regression results are depicted in Supplement S7.

### 3.5. Hypoglycemia

**Figure 6:**
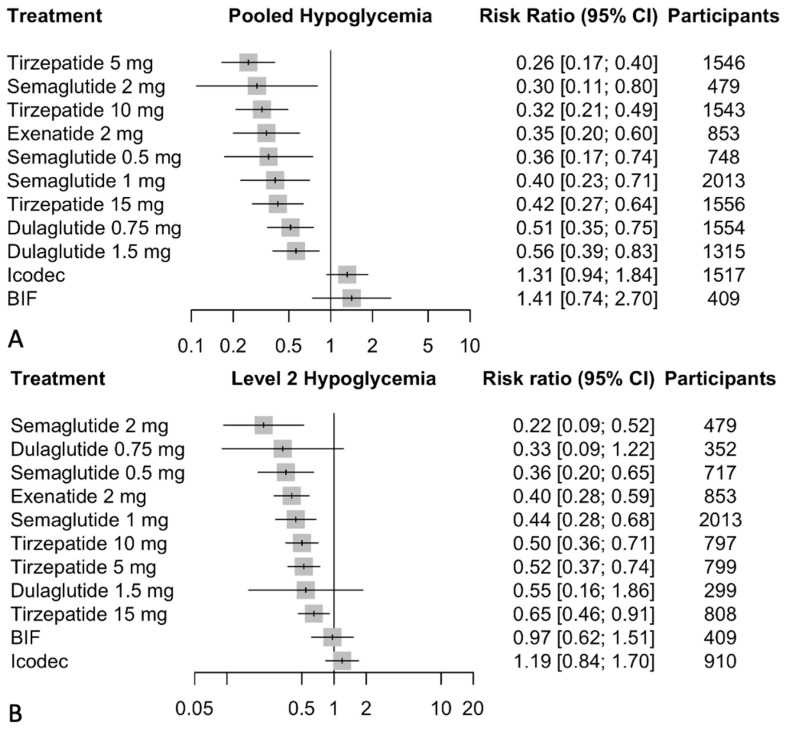
(**A**) Network meta-analysis results for change from baseline in pooled hypoglycemia. (**B**) Network meta-analysis results for change from baseline in level 2 (≤55 mg/dL) hypoglycemia. Effect sizes are presented as risk ratio (RR) and 95% confidence intervals (CI). Treatments are presented according to their effect estimate compared with daily insulin. Abbreviations: BIF: basal insulin Fc.

We conducted a comprehensive analysis to assess the risk of hypoglycemia associated with GLP-1/GIP receptor agonists and weekly insulin analogs, using daily insulin as the baseline comparator. The analysis pooled results from multiple studies that reported level 1 (≤70 mg/dL) and level 2 (≤55 mg/dL) hypoglycemia. It indicated that tirzepatide has a low risk of hypoglycemia, particularly with the 5 mg dose (RR 0.26, 95% CI: 0.17 to 0.40), followed by the 10 mg dose (RR 0.32, 95% CI: 0.21 to 0.49), and the 15 mg dose (RR 0.42, 95% CI: 0.27 to 0.64). Semaglutide also presents a low risk of hypoglycemia across its dosages, with the 2 mg dose showing the most significant effect (RR 0.30, 95% CI: 0.11 to 0.80). Exenatide 2 mg showed a low risk of hypoglycemia as well (RR 0.35, 95% CI: 0.20 to 0.60). In contrast, icodec (RR 1.31, 95% CI: 0.94 to 1.84) and BIF (RR 1.41, 95% CI: 0.74 to 2.70) did not demonstrate a significant reduction in hypoglycemia risk compared to daily insulin. Dulaglutide doses also showed a reduction in hypoglycemia risk, with RRs of 0.56 (95% CI: 0.39 to 0.83) for the 1.5 mg dose and 0.51 (95% CI: 0.35 to 0.75) for the 0.75 mg dose. The network meta-analysis results are presented in Figure 6.

Regarding the subgroup analysis for level 2 hypoglycemia, tirzepatide demonstrated a significant reduction in the risk of severe hypoglycemic episodes across all doses: the 10 mg dose achieved a risk ratio (RR) of 0.50 (95% CI: 0.36 to 0.71), the 5 mg dose an RR of 0.52 (95% CI: 0.37 to 0.74), and the 15 mg dose an RR of 0.65 (95% CI: 0.46 to 0.91), all indicating statistically significant decreases. Similarly, semaglutide showed substantial reductions, with the 2 mg dose showing an RR of 0.22 (95% CI: 0.09 to 0.52), the 1 mg dose an RR of 0.44 (95% CI: 0.28 to 0.68), and the 0.5 mg dose an RR of 0.36 (95% CI: 0.20 to 0.65). Exenatide 2 mg also notably decreased the risk with an RR of 0.40 (95% CI: 0.28 to 0.59). In contrast, dulaglutide, icodec, and BIF did not demonstrate statistically significant effects: dulaglutide’s 1.5 mg and 0.75 mg doses had RRs of 0.55 (95% CI: 0.16 to 1.86) and 0.33 (95% CI: 0.09 to 1.22), respectively; icodec had an RR of 1.19 (95% CI: 0.84 to 1.70); and BIF showed an RR of 0.97 (95% CI: 0.62 to 1.51). The network meta-analysis results are presented in Figure 6.

### 3.6. Incidence of Any Adverse Events

**Figure 7:**
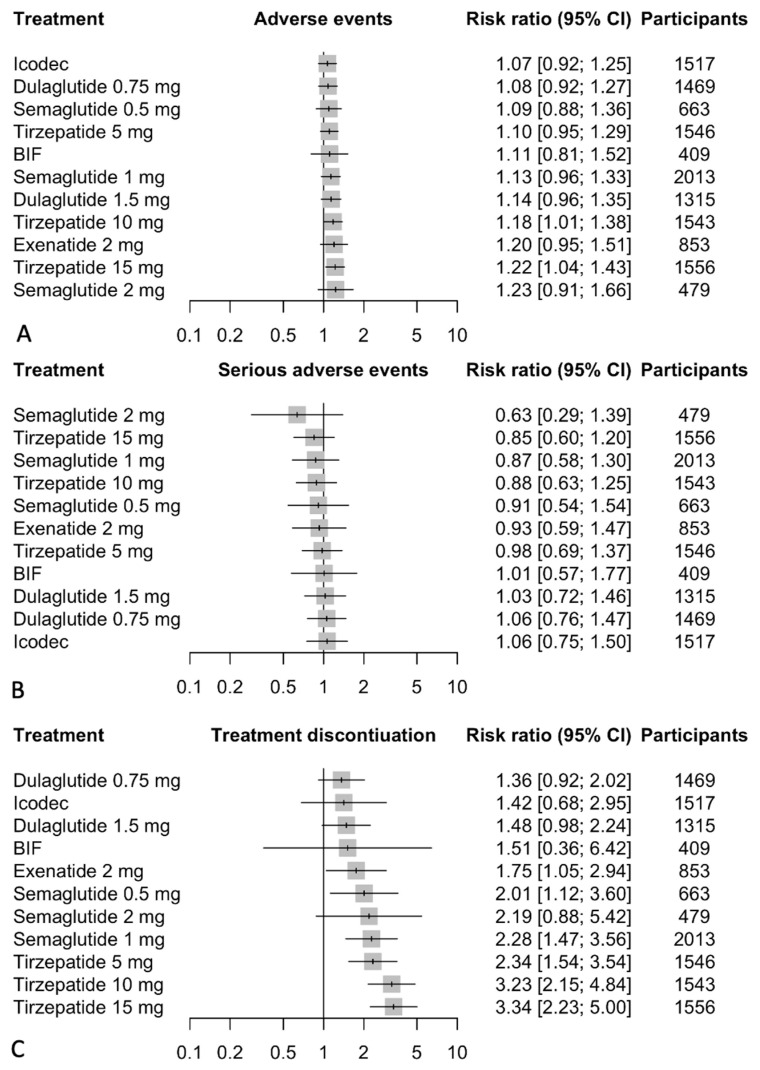
Network meta-analysis results for (**A**) incidence in adverse events. (**B**) incidence in serious adverse events. (**C**) treatment discontinuation due to adverse events compared with daily insulin. Treatments are presented according to their effect estimate compared with daily insulin. Effect sizes are presented as risk ratio (RR) and 95% confidence intervals (CI). Abbreviations: BIF: basal insulin Fc.

Our comprehensive analysis assessed the incidence of adverse events associated with GLP-1/GIP receptor agonists and weekly insulin analogs, using daily insulin as the baseline comparator. The results indicated that tirzepatide had the highest incidence of adverse events, with the 15 mg dose showing a risk ratio (RR) of 1.22 (95% CI: 1.04 to 1.43), followed by the 10 mg dose at an RR of 1.18 (95% CI: 1.01 to 1.38). The 5 mg dose also showed an increased risk of adverse events with an RR of 1.10 (95% CI: 0.95 to 1.29). Semaglutide demonstrated an increased incidence as well, particularly the 1 mg dose with an RR of 1.13 (95% CI: 0.96 to 1.33), and the 2 mg dose with an RR of 1.23 (95% CI: 0.91 to 1.66). Exenatide 2 mg reported a notable increase in adverse events with an RR of 1.20 (95% CI: 0.95 to 1.51). Dulaglutide doses showed an elevated risk with RRs of 1.08 (95% CI: 0.92 to 1.27) for the 0.75 mg dose and 1.14 (95% CI: 0.96 to 1.35) for the 1.5 mg dose. In contrast, icodec and BIF did not demonstrate a statistically significant increase in adverse events compared to daily insulin, with RRs of 1.07 (95% CI: 0.92 to 1.25) and 1.11 (95% CI: 0.81 to 1.52), respectively. The network meta-analysis results are presented in Figure 7.

### 3.7. Incidence of Any Serious Events

Our analysis evaluated the incidence of serious adverse events associated with GLP-1/GIP receptor agonists and weekly insulin analogs, with daily insulin serving as the comparator. The results indicate that the majority of treatments did not demonstrate a statistically significant increase in the risk of serious adverse events compared to daily insulin. Specifically, tirzepatide showed non-significant differences across its doses, with the 15 mg dose having a risk ratio (RR) of 0.85 (95% CI: 0.60 to 1.20), the 10 mg dose an RR of 0.88 (95% CI: 0.63 to 1.25), and the 5 mg dose an RR of 0.98 (95% CI: 0.69 to 1.37). Similarly, semaglutide’s doses all presented non-significant risks, with the 2 mg dose at an RR of 0.63 (95% CI: 0.29 to 1.39), the 1 mg dose at an RR of 0.87 (95% CI: 0.58 to 1.30), and the 0.5 mg dose at an RR of 0.91 (95% CI: 0.54 to 1.54). Exenatide 2 mg and icodec also showed no significant increase in risk with RRs of 0.93 (95% CI: 0.59 to 1.47) and 1.06 (95% CI: 0.75 to 1.50), respectively. The same trend was observed with dulaglutide, where both the 1.5 mg and 0.75 mg doses had non-significant RRs of 1.03 (95% CI: 0.72 to 1.46) and 1.06 (95% CI: 0.76 to 1.47). Additionally, BIF exhibited a reduced risk, though not statistically significant, with an RR of 1.01 (95% CI: 0.57 to 1.77). The network meta-analysis results are presented in Figure 7.

### 3.8. Treatment Discontinuation Due to Adverse Events

Our analysis examined treatment discontinuation due to adverse events among GLP-1/GIP receptor agonists and weekly insulin analogs, using daily insulin as the baseline comparator. The findings reveal that tirzepatide had the highest rates of discontinuation due to adverse events, with the 15 mg dose showing a risk ratio (RR) of 3.34 (95% CI: 2.23 to 5.00), followed closely by the 10 mg dose at an RR of 3.23 (95% CI: 2.15 to 4.84). The 5 mg dose also exhibited a significantly increased risk with an RR of 2.34 (95% CI: 1.54 to 3.54). Semaglutide similarly led to higher discontinuation rates, particularly the 1 mg dose with an RR of 2.28 (95% CI: 1.47 to 3.56), the 2 mg dose at an RR of 2.19 (95% CI: 0.88 to 5.42), and the 0.5 mg dose at an RR of 2.01 (95% CI: 1.12 to 3.60). Exenatide 2 mg also showed a notably high rate of discontinuation due to adverse events with an RR of 1.75 (95% CI: 1.05 to 2.94). In contrast, dulaglutide and icodec had less pronounced effects, with dulaglutide’s 1.5 mg dose showing a borderline significant increase in discontinuation rates (RR 1.48, 95% CI: 0.98 to 2.24), while its 0.75 mg dose and icodec did not show statistically significant increases. BIF also did not demonstrate a significant difference in discontinuation rates compared to daily insulin (RR 1.51, 95% CI: 0.36 to 6.42). The network meta-analysis results are presented in Figure 7. Table 1 below summarizes the results of the study. 

## 4. Discussion

This systematic review and network meta-analysis incorporated data from 23 trials to evaluate the efficacy and safety of GLP-1/GIP receptor agonists and weekly insulin analogs, focusing on their effects on HbA1c reduction, fasting plasma glucose (FPG), body weight (BW), incidence of hypoglycemia, and adverse events. Our analysis not only underscores the variability in efficacy across different treatment modalities but also highlights important safety and tolerability concerns associated with these therapies.

Tirzepatide emerged as the standout treatment, significantly reducing HbA1c levels and body weight across all its dosages when compared to insulin glargine, the active comparator. The superior efficacy of tirzepatide, particularly at the 15 mg dose, is consistent with its dual mechanism of action as both a GLP-1 and GIP receptor agonist, which may offer enhanced metabolic control over single-mechanism treatments. In contrast, weekly insulin analogs like icodec showed modest HbA1c reductions but showed lower efficacy in controlling body weight, sometimes even leading to weight gain. However, these insulins could be a viable option for individuals in low to moderate weight categories where significant weight loss is not a primary treatment goal. This suggests a potential niche for weekly insulins in personalized diabetes management, particularly for patients where weight stability is preferred or where GLP-1/GIP receptor agonists’ effects on weight are contraindicated. These results are consistent with works in the literature that showed 3 to 9 kg weight gain in the first year after starting insulin therapy [47].

The analysis reveals that while higher doses of GLP-1/GIP receptor agonists such as tirzepatide showed higher efficacy, they are also associated with an increased incidence of adverse events and treatment discontinuations. The most common adverse events include gastrointestinal side effects such as nausea, vomiting, and abdominal pain, in addition to hypoglycemia. Gastrointestinal adverse events were more common at higher doses of GLP-1/GIP. On the other hand, hypoglycemia and injection sit reaction were more common with weekly insulin. These findings are consistent with the current literature [48]. 

Lower doses of these agents, while slightly less efficacious in reducing HbA1c and body weight, demonstrate better tolerability, making them suitable for patients who may be sensitive to the side effects of higher doses. Considering the convenience of weekly insulin, our data suggest that a combination therapy involving low doses of GLP-1/GIP agonists with weekly insulin might be a viable strategy for achieving good glycemic control with reduced side effects. This approach would leverage the benefits of both treatments: the metabolic efficacy of GLP-1/GIP agonists and the convenience and tolerability profile of weekly insulin but would increase expense.

The substantial heterogeneity detected in the incidence of adverse events and hypoglycemia across studies suggests that individual patient factors and underlying health conditions play a significant role in the safety profiles of these therapies. The presence of some publication bias for adverse events calls for cautious interpretation of the safety data.

The findings of this meta-analysis have significant implications for clinical practice. The high efficacy of GLP-1/GIP receptor agonists in reducing both HbA1c and body weight makes them attractive options for patients struggling with weight management in addition to glycemic control. However, for patients where weight loss is not necessary or desired, weekly insulins offer an effective alternative that maintains weight stability while still providing good glycemic control. Combining lower doses of GLP-1/GIP agonists with weekly insulin may enhance patient adherence and satisfaction by reducing the frequency of injections and side effects while maintaining efficacy. 

Further research is needed to explore the long-term outcomes of these treatments, particularly regarding cardiovascular health, renal function, and mortality. Additionally, studies should aim to identify patient characteristics that predict better tolerance and response to these therapies, which could enable more personalized treatment approaches.

This investigation has yielded insights into the comparative efficacy, safety, and patient reception of once-weekly GLP-1/GIP agonist treatments and basal insulin in the context of type 2 diabetes mellitus (T2DM). There are, however, several limitations to our study. The novelty of the examined therapies has resulted in a limited selection of studies for inclusion, which, in turn, has introduced a notable degree of heterogeneity to our findings. Moreover, the analysis predominantly reflects short-term effects, underscoring the need for extended longitudinal research to comprehensively discern the long-term impacts of these treatments on the progression of T2DM, associated complications, and the quality of life of the patients. Variations in the duration of studies, as well as the demographic and baseline health characteristics of participants, inject a level of variability that may limit the broad applicability of our findings. The methodology of network meta-analysis, dependent on indirect treatment comparisons, is inherently complex and operates under assumptions like transitivity and consistency that are critical to the integrity of our conclusions. Inconsistencies in defining and documenting adverse events in clinical trials further complicate the precision of our adverse event data. Finally, the very nature of meta-analysis, contingent on the quality and inclusiveness of existing research, carries an omnipresent risk of publication bias that cannot be completely negated.

## 5. Conclusions

In conclusion, this network meta-analysis provides comprehensive insights into the comparative efficacy and safety of GLP-1/GIP receptor agonists and weekly insulin analogs. It highlights the need for balancing efficacy with safety in diabetes management, and the importance of individualizing treatment plans based on patient-specific factors and preferences. Our findings underscore the superior efficacy of GLP-1/GIP agonists in both glycemic control and weight management. However, weekly insulins remain a crucial part of the therapeutic arsenal, especially for individuals where significant weight loss is not desired or patients who cannot tolerate GLP-1/GIP agonists. The integration of these agents into clinical practice should consider patient-specific factors such as baseline body weight, potential side effects, and individual health goals to optimize outcomes in diabetes care.

## Figures and Tables

**Table 1:** Summary of the results of the network meta-analysis. Abbreviations: HbA1c: hemoglobin A1c, MD: mean difference, CI: confidence interval, BIF: basal insulin Fc, BW: body weight, FPG: fasting plasma glucose, RR: risk ratio, mg: milligrams, kg: kilograms, mmol/L: millimoles per liter.

Outcome	Most Favorable Intervention	Treatment Effect	Least Favorable Intervention	Treatment Effect
Change in HbA1c	Tirzepatide 15 mg	MD: −1.29% (95% CI: −1.44 to −1.14)	BIF	MD: 0.08 (95% CI: −0.17 to 0.33)
Change in FPG	Tirzepatide 15 mg	MD: −0.70 mmol/L (95% CI: −1.00 to −0.41)	Dulaglutide 0.75 mg	MD: 1.10 (95% CI: 0.82 to 1.38)
Change in BW	Tirzepatide 15 mg	MD: −12.39 kg (95% CI: −13.32 to −11.46)	BIF	MD: 0.71 (95% CI: −0.68 to 2.10)
Pooled hypoglycemia	Tirzepatide 5 mg	RR: 0.26 (95% CI: 0.17 to 0.40)	BIF	RR: 1.41 (95% CI: 0.74 to 2.70)
Level 2 hypoglycemia	Semaglutide 2 mg	RR: 0.22 (95% CI: 0.09 to 0.52)	Icodec	RR: 1.19 (95% CI: 0.84 to 1.70)
Incidence of any adverse events	Icodec	RR: 1.07 (95% CI: 0.92 to 1.25)	Semaglutide 2 mg	RR: 1.23 (95% CI: 0.91 to 1.66)
Serious adverse events	Semaglutide 2 mg	RR: 0.63 (95% CI: 0.29 to 1.39)	Icodec	RR: 1.06 (95% CI: 0.75 to 1.50)
Treatment discontinuation due to adverse events	Dulaglutide 0.75 mg	RR: 1.36 (95% CI: 0.92 to 2.02)	Tirzepatide 15 mg	RR: 3.34 (95% CI: 2.23 to 5.00)

## Data Availability

The original contributions presented in the study are included in the article/Supplementary Materials, further inquiries can be directed to the corresponding author.

## Supplementary Materials

The following supporting information can be downloaded at: https://www.mdpi.com/article/10.3390/biomedicines12091943/s1. Supplement S1: Search strategy. Supplement S2: Baseline characteristics of included studies. Supplement S3: Risk of bias assessment of included trials for each outcome. Supplement S4: Comparison-adjusted funnel plots. Supplement S5: Pairwise meta-analysis results. Supplement S6: Certainty of the effect estimates. Supplement S7: Meta-regression results. Supplement S8: Summary of the excluded studies. Supplement S9: Evaluation of heterogeneity and inconsistency under full design-by-treatment interaction random effects model. References [22,23,24,25,26,27,28,29,30,31,32,33,34,35,36,37,38,39,40,41,42,43,44,45,46,49,50,51,52,53,54,55,56,57,58] are cited in the supplementary materials.

## Abbreviations

DM	Diabetes mellitus
GLP-1	Glucagon-like peptide-1
GIP	Glucose-dependent insulinotropic peptide
NMA	Network meta-analysis
RCT	Randomized controlled trial
HbA1c	Hemoglobin A1c
BW	Body weight
FPG	Fasting plasma glucose
TEAE	Treatment-emergent adverse events
SE	Standard error
CI	Confidence interval
RR	Risk ratio
T2DM	Type 2 diabetes mellitus
SUCRA	Surface under the cumulative ranking curve
PRISMA	Preferred reporting items for systematic reviews and meta-analyses

## References

[1] Leon B.M., Maddox T.M. (2015). Diabetes and cardiovascular disease: Epidemiology, biological mechanisms, treatment recommendations and future research. World J. Diabetes.

[2] Fan W. (2017). Epidemiology in diabetes mellitus and cardiovascular disease. Cardiovasc. Endocrinol..

[3] Kerr D., Rajpura J.R., Namvar T. (2024). Evaluating Patient and Provider Preferences for a Once-Weekly Basal Insulin in Adults with Type 2 Diabetes. Patient Prefer. Adherence.

[4] Cornell S. (2020). A review of GLP-1 receptor agonists in type 2 diabetes: A focus on the mechanism of action of once-weekly agents. J. Clin. Pharm. Ther..

[5] Rizvi A.A., Rizzo M. (2022). The Emerging Role of Dual GLP-1 and GIP Receptor Agonists in Glycemic Management and Cardiovascular Risk Reduction. Diabetes Metab. Syndr. Obes..

[6] Scheen A.J. (2023). Dual GIP/GLP-1 receptor agonists: New advances for treating type-2 diabetes. Ann. D’endocrinologie.

[7] Ayesh H. (2024). Comparative Efficacy and Safety of Weekly GLP-1/GIP Agonists versus Weekly Insulin in Type 2 Diabetes: A Network Meta-Analysis of Randomized Controlled Trials. https://osf.io/p7szu.

[8] Page M.J., McKenzie J.E., Bossuyt P.M., Boutron I., Hoffmann T.C., Mulrow C.D., Shamseer L., Tetzlaff J.M., Akl E.A., Brennan S.E. (2021). The PRISMA 2020 statement: An updated guideline for reporting systematic reviews. BMJ.

[9] Altman D.G., Bland J.M. (2005). Standard deviations and standard errors. BMJ.

[10] Wan X., Wang W., Liu J., Tong T. (2014). Estimating the sample mean and standard deviation from the sample size, median, range and/or interquartile range. BMC Med. Res. Methodol..

[11] Sterne J.A.C., Savović J., Page M.J., Elbers R.G., Blencowe N.S., Boutron I., Cates C.J., Cheng H.-Y., Corbett M.S., Eldridge S.M. (2019). RoB 2: A revised tool for assessing risk of bias in randomised trials. BMJ.

[12] Shim S.R., Kim S.J., Lee J., Rücker G. (2019). Network meta-analysis: Application and practice using R software. Epidemiol. Health.

[13] Dias S., Caldwell D.M. (2019). Network meta-analysis explained. Arch. Dis. Child. Fetal Neonatal Ed..

[14] Jansen J.P., Fleurence R., Devine B., Itzler R., Barrett A., Hawkins N., Lee K., Boersma C., Annemans L., Cappelleri J.C. (2011). Interpreting indirect treatment comparisons and network meta-analysis for health-care decision making: Report of the ISPOR Task Force on Indirect Treatment Comparisons Good Research Practices: Part 1. Value Health.

[15] Rhodes K.M., Turner R.M., Higgins J.P. (2015). Predictive distributions were developed for the extent of heterogeneity in meta-analyses of continuous outcome data. J. Clin. Epidemiol..

[16] Mbuagbaw L., Rochwerg B., Jaeschke R., Heels-Andsell D., Alhazzani W., Thabane L., Guyatt G.H. (2017). Approaches to interpreting and choosing the best treatments in network meta-analyses. Syst. Rev..

[17] RStudio Team (2020). RStudio: Integrated Development Environment for R.

[18] Balduzzi S., Rücker G., Nikolakopoulou A., Papakonstantinou T., Salanti G., Efthimiou O., Schwarzer G. (2023). netmeta: An R Package for Network Meta-Analysis Using Frequentist Methods. J. Stat. Softw..

[19] van Valkenhoef G., Kuiper J. (2023). gemtc: Network Meta-Analysis Using Bayesian Methods. https://cran.r-project.org/web/packages/gemtc/gemtc.pdf.

[20] Plummer M. (2024). rjags: Bayesian Graphical Models Using MCMC. https://cran.r-project.org/web//packages/rjags/rjags.pdf.

[21] Nikolakopoulou A., Higgins J.P.T., Papakonstantinou T., Chaimani A., Del Giovane C., Egger M., Salanti G. (2020). CINeMA: An approach for assessing confidence in the results of a network meta-analysis. PLoS Med..

[22] Ahmann A.J., Capehorn M., Charpentier G., Dotta F., Henkel E., Lingvay I., Holst A.G., Annett M.P., Aroda V.R. (2018). Efficacy and Safety of Once-Weekly Semaglutide Versus Exenatide ER in Subjects With Type 2 Diabetes (SUSTAIN 3): A 56-Week, Open-Label, Randomized Clinical Trial. Diabetes Care.

[23] Aroda V.R., Bain S.C., Cariou B., Piletic M., Rose L., Axelsen M., Rowe E., DeVries J.H. (2017). Efficacy and safety of once-weekly semaglutide versus once-daily insulin glargine as add-on to metformin (with or without sulfonylureas) in insulin-naive patients with type 2 diabetes (SUSTAIN 4): A randomised, open-label, parallel-group, multicentre, multinational, phase 3a trial. Lancet Diabetes Endocrinol..

[24] Bajaj H.S., Bergenstal R.M., Christoffersen A., Davies M.J., Gowda A., Isendahl J., Lingvay I., Senior P.A., Silver R.J., Trevisan R. (2021). Switching to Once-Weekly Insulin Icodec Versus Once-Daily Insulin Glargine U100 in Type 2 Diabetes Inadequately Controlled on Daily Basal Insulin: A Phase 2 Randomized Controlled Trial. Diabetes Care.

[25] Blonde L., Jendle J., Gross J., Woo V., Jiang H., Fahrbach J.L., Milicevic Z. (2015). Once-weekly dulaglutide versus bedtime insulin glargine, both in combination with prandial insulin lispro, in patients with type 2 diabetes (AWARD-4): A randomised, open-label, phase 3, non-inferiority study. Lancet.

[26] Diamant M., Van Gaal L., Stranks S., Northrup J., Cao D., Taylor K., Trautmann M. (2010). Once weekly exenatide compared with insulin glargine titrated to target in patients with type 2 diabetes (DURATION-3): An open-label randomised trial. Lancet.

[27] Frias J., Chien J., Zhang Q., Chigutsa E., Landschulz W., Syring K., Wullenweber P., Haupt A., Kazda C. (2023). Safety and efficacy of once-weekly basal insulin Fc in people with type 2 diabetes previously treated with basal insulin: A multicentre, open-label, randomised, phase 2 study. Lancet Diabetes Endocrinol..

[28] Frías J.P., Auerbach P., Bajaj H.S., Fukushima Y., Lingvay I., Macura S., Søndergaard A.L., Tankova T.I., Tentolouris N., Buse J.B. (2021). Efficacy and safety of once-weekly semaglutide 2·0 mg versus 1·0 mg in patients with type 2 diabetes (SUSTAIN FORTE): A double-blind, randomised, phase 3B trial. Lancet Diabetes Endocrinol..

[29] Frías J.P., Davies M.J., Rosenstock J., Pérez Manghi F.C., Fernández Landó L., Bergman B.K., Liu B., Cui X., Brown K. (2021). Tirzepatide versus Semaglutide Once Weekly in Patients with Type 2 Diabetes. N. Engl. J. Med..

[30] Gao L., Lee B.W., Chawla M., Kim J., Huo L., Du L., Huang Y., Ji L. (2023). Tirzepatide versus insulin glargine as second-line or third-line therapy in type 2 diabetes in the Asia-Pacific region: The SURPASS-AP-Combo trial. Nat. Med..

[31] Giorgino F., Benroubi M., Sun J.H., Zimmermann A.G., Pechtner V. (2015). Efficacy and Safety of Once-Weekly Dulaglutide Versus Insulin Glargine in Patients With Type 2 Diabetes on Metformin and Glimepiride (AWARD-2). Diabetes Care.

[32] Inagaki N., Atsumi Y., Oura T., Saito H., Imaoka T. (2012). Efficacy and safety profile of exenatide once weekly compared with insulin once daily in Japanese patients with type 2 diabetes treated with oral antidiabetes drug(s): Results from a 26-week, randomized, open-label, parallel-group, multicenter, noninferiority study. Clin. Ther..

[33] Inagaki N., Takeuchi M., Oura T., Imaoka T., Seino Y. (2022). Efficacy and safety of tirzepatide monotherapy compared with dulaglutide in Japanese patients with type 2 diabetes (SURPASS J-mono): A double-blind, multicentre, randomised, phase 3 trial. Lancet Diabetes Endocrinol..

[34] Kimura T., Katakura Y., Shimoda M., Kawasaki F., Yamabe M., Tatsumi F., Matsuki M., Iwamoto Y., Anno T., Fushimi Y. (2023). Comparison of clinical efficacy and safety of weekly glucagon-like peptide-1 receptor agonists dulaglutide and semaglutide in Japanese patients with type 2 diabetes: Randomized, parallel-group, multicentre, open-label trial (COMING study). Diabetes Obes. Metab..

[35] Lingvay I., Asong M., Desouza C., Gourdy P., Kar S., Vianna A., Vilsboll T., Vinther S., Mu Y. (2023). Once-Weekly Insulin Icodec vs Once-Daily Insulin Degludec in Adults With Insulin-Naive Type 2 Diabetes: The ONWARDS 3 Randomized Clinical Trial. JAMA.

[36] Ludvik B., Giorgino F., Jodar E., Frias J.P., Fernandez Lando L., Brown K., Bray R., Rodriguez A. (2021). Once-weekly tirzepatide versus once-daily insulin degludec as add-on to metformin with or without SGLT2 inhibitors in patients with type 2 diabetes (SURPASS-3): A randomised, open-label, parallel-group, phase 3 trial. Lancet.

[37] Mathieu C., Ásbjörnsdóttir B., Bajaj H.S., Lane W., Matos A., Murthy S., Stachlewska K., Rosenstock J. (2023). Switching to once-weekly insulin icodec versus once-daily insulin glargine U100 in individuals with basal-bolus insulin-treated type 2 diabetes (ONWARDS 4): A phase 3a, randomised, open-label, multicentre, treat-to-target, non-inferiority trial. Lancet.

[38] Philis-Tsimikas A., Asong M., Franek E., Jia T., Rosenstock J., Stachlewska K., Watada H., Kellerer M. (2023). Switching to once-weekly insulin icodec versus once-daily insulin degludec in individuals with basal insulin-treated type 2 diabetes (ONWARDS 2): A phase 3a, randomised, open label, multicentre, treat-to-target trial. Lancet Diabetes Endocrinol..

[39] Pratley R.E., Aroda V.R., Lingvay I., Lüdemann J., Andreassen C., Navarria A., Viljoen A. (2018). Semaglutide versus dulaglutide once weekly in patients with type 2 diabetes (SUSTAIN 7): A randomised, open-label, phase 3b trial. Lancet Diabetes Endocrinol..

[40] Del Prato S., Kahn S.E., Pavo I., Weerakkody G.J., Yang Z., Doupis J., Aizenberg D., Wynne A.G., Riesmeyer J.S., Heine R.J. (2021). Tirzepatide versus insulin glargine in type 2 diabetes and increased cardiovascular risk (SURPASS-4): A randomised, open-label, parallel-group, multicentre, phase 3 trial. Lancet.

[41] Rosenstock J., Bain S.C., Gowda A., Jodar E., Liang B., Lingvay I., Nishida T., Trevisan R., Mosenzon O., Investigators O.T. (2023). Weekly Icodec versus Daily Glargine U100 in Type 2 Diabetes without Previous Insulin. N. Engl. J. Med..

[42] Rosenstock J., Bajaj H.S., Janež A., Silver R., Begtrup K., Hansen M.V., Jia T., Goldenberg R. (2020). Once-weekly insulin for type 2 diabetes without previous insulin treatment. N. Engl. J. Med..

[43] Takahashi Y., Nomoto H., Yokoyama H., Takano Y., Nagai S., Tsuzuki A., Cho K.Y., Miya A., Kameda H., Takeuchi J. (2023). Improvement of glycaemic control and treatment satisfaction by switching from liraglutide or dulaglutide to subcutaneous semaglutide in patients with type 2 diabetes: A multicentre, prospective, randomized, open-label, parallel-group comparison study (SWITCH-SEMA 1 study). Diabetes Obes. Metab..

[44] Tuttle K.R., Lakshmanan M.C., Rayner B., Busch R.S., Zimmermann A.G., Woodward D.B., Botros F.T. (2018). Dulaglutide versus insulin glargine in patients with type 2 diabetes and moderate-to-severe chronic kidney disease (AWARD-7): A multicentre, open-label, randomised trial. Lancet Diabetes Endocrinol..

[45] Wang J., Li H.-Q., Xu X.-H., Kong X.-C., Sun R., Jing T., Ye L., Su X.-F., Ma J.-H. (2019). The Effects of Once-Weekly Dulaglutide and Insulin Glargine on Glucose Fluctuation in Poorly Oral-Antidiabetic Controlled Patients with Type 2 Diabetes Mellitus. BioMed Res. Int..

[46] Bue-Valleskey J.M., Kazda C.M., Ma C., Chien J., Zhang Q., Chigutsa E., Landschulz W., Haupt A., Frias J.P. (2023). Once-Weekly Basal Insulin Fc Demonstrated Similar Glycemic Control to Once-Daily Insulin Degludec in Insulin-Naive Patients with Type 2 Diabetes: A Phase 2 Randomized Control Trial. Diabetes Care.

[47] Brown A., Guess N., Dornhorst A., Taheri S., Frost G. (2017). Insulin-associated weight gain in obese type 2 diabetes mellitus patients: What can be done?. Diabetes Obes. Metab..

[48] Hayes M.R., Borner T., De Jonghe B.C. (2021). The Role of GIP in the Regulation of GLP-1 Satiety and Nausea. Diabetes.

[49] Umpierrez G., Tofé Povedano S., Pérez Manghi F., Shurzinske L., Pechtner V. (2014). Efficacy and safety of dulaglutide monotherapy versus metformin in type 2 diabetes in a randomized controlled trial (AWARD-3). Diabetes Care.

[50] Wysham C., Blevins T., Arakaki R., Colon G., Garcia P., Atisso C., Kuhstoss D., Lakshmanan M. (2014). Efficacy and safety of dulaglutide added onto pioglitazone and metformin versus exenatide in type 2 diabetes in a randomized controlled trial (AWARD-1). Diabetes Care.

[51] Weinstock R.S., Guerci B., Umpierrez G., Nauck M.A., Skrivanek Z., Milicevic Z. (2015). Safety and efficacy of once-weekly dulaglutide versus sitagliptin after 2 years in metformin-treated patients with type 2 diabetes (AWARD-5): A randomized, phase III study. Diabetes Obes. Metab..

[52] Dungan K.M., Povedano S.T., Forst T., Gonzalez J.G.G., Atisso C., Sealls W., Fahrbach J.L. (2014). Once-weekly dulaglutide versus once-daily liraglutide in metformin-treated patients with type 2 diabetes (AWARD-6): A randomised, open-label, phase 3, non-inferiority trial. Lancet.

[53] Dungan K.M., Weitgasser R., Perez Manghi F., Pintilei E., Fahrbach J.L., Jiang H.H., Shell J., Robertson K.E. (2016). A 24-week study to evaluate the efficacy and safety of once-weekly dulaglutide added on to glimepiride in type 2 diabetes (AWARD-8). Diabetes Obes. Metab..

[54] Pozzilli P., Norwood P., Jodar E., Davies M.J., Ivanyi T., Jiang H., Woodward D.B., Milicevic Z. (2017). Placebo-controlled, randomized trial of the addition of once-weekly glucagon-like peptide-1 receptor agonist dulaglutide to titrated daily insulin glargine in patients with type 2 diabetes (AWARD-9). Diabetes Obes. Metab..

[55] Chen Y.H., Huang C.-N., Cho Y.M., Li P., Gu L., Wang F., Yang J., Wang W.Q. (2018). Efficacy and safety of dulaglutide monotherapy compared with glimepiride in East-Asian patients with type 2 diabetes in a multicentre, double-blind, randomized, parallel-arm, active comparator, phase III trial. Diabetes Obes. Metab..

[56] Battelino T., Bergenstal R.M., Rodriguez A., Fernandez Lando L., Bray R., Tong Z., Brown K. (2022). Efficacy of once-weekly tirzepatide versus once-daily insulin degludec on glycaemic control measured by continuous glucose monitoring in adults with type 2 diabetes (SURPASS-3 CGM): A substudy of the randomised, open-label, parallel-group, phase 3 SURPASS-3 trial. Lancet Diabetes Endocrinol..

[57] Gastaldelli A., Cusi K., Fernández Landó L., Bray R., Brouwers B., Rodríguez Á. (2022). Effect of tirzepatide versus insulin degludec on liver fat content and abdominal adipose tissue in people with type 2 diabetes (SURPASS-3 MRI): A substudy of the randomised, open-label, parallel-group, phase 3 SURPASS-3 trial. Lancet Diabetes Endocrinol..

[58] Wilson J.M., Nikooienejad A., Robins D.A., Roell W.C., Riesmeyer J.S., Haupt A., Duffin K.L., Taskinen M.R., Ruotolo G. (2020). The dual glucose-dependent insulinotropic peptide and glucagon-like peptide-1 receptor agonist, tirzepatide, improves lipoprotein biomarkers associated with insulin resistance and cardiovascular risk in patients with type 2 diabetes. Diabetes Obes. Metab..

