# Supplementary material for: Comparative Efficacy and Safety of Weekly GLP-1/GIP Agonists vs. Weekly Insulin in Type 2 Diabetes: A Network Meta-Analysis of Randomized Controlled Trials
Source: Biomedicines. 2024 Aug 23;12(9):1943. doi: 10.3390/biomedicines12091943 (PMC11428590; doi:10.3390/biomedicines12091943)
Supplement: Supplementary file 1 [file biomedicines-12-01943-s001.zip › biomedicines-3154906-supplementary.pdf]

Table of Contents

Supplement S1: Search strategy *2*

Supplement S2: Baseline characteristics of included studies *3*

Supplement S3. Risk of bias assessment of included trials for each outcome *8*

S3.1 Change from baseline in HbA1c (%) **8**

S3.2 Change from baseline in FPG (mmol/L) **11**

S3.3 Change from baseline in BW (kg) **14**

S3.4 Change from baseline in TEAE (OR) **17**

S3.5 Change from baseline in the risk of hypoglycemia (OR) **20**

S3.6 Proportion of serious adverse events (OR) **23**

S3.7 Proportion of treatment discontinuation due to adverse events (OR) **26**

Supplement S4. Comparison-adjusted funnel plots *29*

S4.1 Comparison adjusted funnel plot for change from baseline in HbA1c **29**

S4.2 Comparison adjusted funnel plot for change from baseline in BW **29**

S4.3 Comparison adjusted funnel plot for change from baseline in FPG **30**

S4.4 Comparison adjusted funnel plot for incidence of TEAE (RR) **31**

S4.5 Comparison adjusted funnel plot for incidence of pooled hypoglycemia (RR) **31**

S4.6 Comparison adjusted funnel plot for incidence of treatment discontinuation due to adverse events (RR) **32**

S4.7 Comparison adjusted funnel plot for incidence of serious adverse events (RR) **33**

Supplement S5. Pairwise meta-analysis results *35*

S5.1 Pairwise meta-analysis results for change in HbA1c **35**

S5.2 Pairwise meta-analysis results for change in BW **36**

S5.3 Pairwise meta-analysis results for change in FPG **37**

Supplement S6. Certainty of the effect estimates *39*

S6.1 Certainty in effect estimates for change in HbA1c **39**

S6.2 Certainty in effect estimates for change in FPG **47**

S6.3 Certainty in effect estimates for change in BW **53**

S6.4 Certainty in effect estimates for incidence of TEAE **59**

S6.5 Certainty in effect estimates for incidence of hypoglycemia **65**

S6.6 Certainty in effect estimates for incidence of treatment discontinuation due to adverse events **71**

S6.7 Certainty in effect estimates for the incidence of serious adverse events **76**

Supplement S7. Meta-regression results: *83*

S7.1 Meta-regression results for HbA1c (age, diabetes duration) **83**

S7.2 Meta-regression results for FPG (age, diabetes duration) **84**

S7.3 Meta-regression results for BW (age, diabetes duration) **85**

.... *85*

Supplement S8. Summary of the excluded studies: *86*

Supplement S9. Evaluation of heterogeneity and inconsistency under full design-by-treatment interaction random effects model *87*

References: *91*

Supplement S1: Search strategy

From inception to April 5th, 2024:

PubMed

("Dulaglutide"[Mesh] OR "Exenatide"[Mesh] OR "Semaglutide"[Mesh] OR "Semaglutide and Tirzepatide"[Mesh] OR "Tirzepatide"[Mesh] OR "Ozempic" OR "Trulicity" OR "Bydureon" OR "Mounjaro" OR "weekly GLP-1 agonist" OR "weekly GLP-1/GIP agonist") AND "Diabetes Mellitus"[Mesh] AND "Clinical Trial"[Publication Type]

("Insulin Glargine"[Mesh] OR "Insulin Degludec"[Mesh] OR "BIF insulin" OR "Efistora" OR "weekly insulin" OR "insulin glargine" OR "insulin degludec" OR "insulin detemir" OR "weekly insulin icodec") AND "Diabetes Mellitus"[Mesh] AND "Clinical Trial"[Publication Type]

Web of Science
("Dulaglutide"[Mesh] OR "Exenatide"[Mesh] OR "Semaglutide"[Mesh] OR "Semaglutide and Tirzepatide"[Mesh] OR "Tirzepatide"[Mesh] OR "Ozempic" OR "Trulicity" OR "Bydureon" OR "Mounjaro" OR "weekly GLP-1 agonist" OR "weekly GLP-1/GIP agonist") AND "Diabetes Mellitus"[Mesh] AND "Clinical Trial"[Publication Type]

("Insulin Glargine"[Mesh] OR "Insulin Degludec"[Mesh] OR "BIF insulin" OR "Efistora" OR "weekly insulin" OR "insulin glargine" OR "insulin degludec" OR "insulin detemir" OR "weekly insulin icodec") AND "Diabetes Mellitus"[Mesh] AND "Clinical Trial"[Publication Type]

Scopus
("Dulaglutide"[Mesh] OR "Exenatide"[Mesh] OR "Semaglutide"[Mesh] OR "Semaglutide and Tirzepatide"[Mesh] OR "Tirzepatide"[Mesh] OR "Ozempic" OR "Trulicity" OR "Bydureon" OR "Mounjaro" OR "weekly GLP-1 agonist" OR "weekly GLP-1/GIP agonist") AND "Diabetes Mellitus"[Mesh] AND "Clinical Trial"[Publication Type]

("Insulin Glargine"[Mesh] OR "Insulin Degludec"[Mesh] OR "BIF insulin" OR "Efistora" OR "weekly insulin" OR "insulin glargine" OR "insulin degludec" OR "insulin detemir" OR "weekly insulin icodec") AND "Diabetes Mellitus"[Mesh] AND "Clinical Trial"[Publication Type]

Cochrane
("Dulaglutide"[Mesh] OR "Exenatide"[Mesh] OR "Semaglutide"[Mesh] OR "Semaglutide and Tirzepatide"[Mesh] OR "Tirzepatide"[Mesh] OR "Ozempic" OR "Trulicity" OR "Bydureon" OR "Mounjaro" OR "weekly GLP-1 agonist" OR "weekly GLP-1/GIP agonist") AND "Diabetes Mellitus"[Mesh] AND "Clinical Trial"[Publication Type]

("Insulin Glargine"[Mesh] OR "Insulin Degludec"[Mesh] OR "BIF insulin" OR "Efistora" OR "weekly insulin" OR "insulin glargine" OR "insulin degludec" OR "insulin detemir" OR "weekly insulin icodec") AND "Diabetes Mellitus"[Mesh] AND "Clinical

Supplement S2: Baseline characteristics of included studies

Study	Registration	Country and region	Design	Follow-up duration	Randomized treatments	Duration of type 2 diabetes	Age (mean +- SD), years	Male %	BMI (mean +/- SD), kg/m2	HbA1c(mean+/- SD), %	
**Ahman,2018** [1]	NCT01885208	Argentina, Croatia, Finland, France, Germany, Greece, Italy, Netherlands, Puerto Rico, Serbia, Switzerland, UK, US	RCT Phase 3	56 weeks	Semaglutide 1.0 mg: 404<br>Exenatide 2.0 mg: 405<br>**Total: 809**	9.0 (0.4–37.1)<br>9.4 (0.3–54.0)<br>**9.2 (0.3–54.0)**	56.4 (20–82)<br>56.7 (21–83)<br>**56.6 (20–83)**	54.2<br>56.3<br>**55.3**	34.0(21.0–72.8)<br>33.6(21.2–55.8)<br>**33.8 (21.1–72.8)**	8.4(6.7–11.1)<br>8.3(6.5–11.2)<br>**8.3 (6.5–11.2**	Metformin +/- sulfonylurea
**Aroda,2017** [2]	NCT02128932	Argentina, Croatia, France, Germany, India, Mexico, Netherlands, North Macedonia, Puerto Rico, Romania, Slovakia, Slovenia, South Africa, UK, US	RCT Phase 3	30 weeks	Semaglutide 0.5 mg: 362<br>Semaglutide 1.0 mg: 360<br>Glargine: 360<br>**Total: 1082**	7.8(5.1)<br>9.3(7.2)<br>8.6(6.3)<br>**8.6(6.3)**	56.5(10.3)<br>56.7(10.4)<br>56.2(10.6)<br>**56.5(10.4)**	54.0<br>51.0<br>54.0<br>**53.0**	33.1(6.5)<br>33.0(6.5)<br>33.0(6.5)<br>**33.0(6.5)**	8.1(0.8)<br>8.3(0.9)<br>8.1(0.9)<br>**8.2(0.9)**	Metformin +/- sulfonylurea
**Bajaj,2021** [3]	NCT03922750	Canada, Czech Republic, Germany, Italy, US	RCT Phase 2	16 weeks	Icodec LD: 54<br>Icodec NLD: 50<br>Glargine: 50<br>**Total: 154**	13.8(7.7)<br>16.8(8.2)<br>14.8(8.1)<br>**15.1(7.8)**	62.4(7.2)<br>62.1(8.2)<br>60.5(7.9)<br>**62.0(7.6)**	72.0<br>78.0<br>66.0<br>**72.0**	30.2 (4.3)<br>29.0(4.1)<br>30.3(5.0)<br>**29.7(4.3)**	7.8(0.7)<br>7.9(0.7)<br>7.9(0.7)<br>**7.8(0.7)**	Metformin +/- SGLT2is, DPP4 inhibitors +/- insulin 10-50 units daily
**Blonde,2015** [4]	NCT01191268	Argentina, Australia, Belgium, Brazil, Canada, Denmark, Greece, Hungary, Mexico, Poland, Russia, Spain, Sweden, Taiwan, USA	RCT Phase 3	52 weeks	Dulaglutide 1.5 mg: 295<br>Dulaglutide 0.75 mg: 293<br>Glargine: 296<br>**Total: 884**	12.8(7.2)<br>12.4(6.9)<br>13.0(6.8)<br>**12.7(6.9)**	58.9(9.6)<br>59.3(9.0)<br>59.9(9.1)<br>**59.3(9.2)**	54.0<br>50.0<br>56.0<br>**53.0**	32.0(5.1)<br>33.1(5.2)<br>32.4(5.3)<br>**32.5(5.2)**	8.4(1.0)<br>8.0(1.0)<br>8.5(1.0)<br>**8.4(1.0)**	Metformin

**Bue-Valleskey,2023**[5]	NCT04450394	Argentina, Germany, Poland, Puerto Rico, USA	RCT Phase 2	26 weeks	BIF: 143<br>Degludec: 135<br>**Total: 278**	10.4(6.8)<br>9.7(6.0)<br>**10.1(6.4)**	57.3(9.7)<br>59.4(9.1)<br>**58.3(9.5)**	53.1<br>56.3<br>**54.7**	32.3 (5.4)<br>31.6(5.5)<br>**32.0(5.5)**	8.1 (0.8)<br>8.0 (0.8)<br>8.0(0.8)	Metformin +/- SGLT2is, DPP4 inhibitors
**Diamant,2010**[6]	NCT00641056	Australia, Belgium, Czech Republic, Denmark, France, Germany, Greece, Hungary, Korea, Mexico, Netherlands, Puerto Rico, Russia, Spain, Taiwan, US	RCT Phase 3	26 weeks	Exenatide: 233<br>Glargine: 223<br>**Total: 466**	8.0(6.0)<br>7.8(6.0)<br>**7.9(6.0)**	58.0(10.0)<br>58.0(9.0)<br>**58.0(9.0)**	52<br>55<br>**53.5**	32.0(5.0)<br>32.0(5.0)<br>**32.0(5.0)**	8.3(1.1)<br>8.3(1.0)<br>**8.3(1.0)**	Metformin +/- sulfonylurea
**Frias,2021**[7]	NCT03989232	Bulgaria, Canada, Czechia, Greece, Hungary, Japan, Poland, Puerto Rico, Slovakia, Ukraine, US	RCT Phase 3	40 weeks	Semaglutide 1 mg: 481<br>Semaglutide 2 mg: 480<br>**Total: 961**	9.9(6.2)<br>9.2(6.2)<br>**9.5(6.2)**	58.2(9.9)<br>57.9(10.0)<br>**58.0(10.0)**	59.0<br>58.0<br>**59.0**	34.4(7.2)<br>34.8(6.8)<br>**34.6(7.0)**	8.8(0.6)<br>8.9(0.6)<br>**8.9(0.6)**	Metformin +/- sulfonylurea
**Frias,2022**[8]	NCT03987919	Argentina, Australia, Brazil, Canada, Israel, Mexico, Puerto Rico, UK, US	RCT Phase 3	40 weeks	Tirzepatide 5 mg:470<br>Tirzepatide 10 mg:469<br>Tirzepatide 15 mg:470<br>Semaglutide 1 mg: 469<br>**Total: 1878**	9.1(7.1)<br>8.4(5.9)<br>8.7(6.8)<br>8.3(5.8)<br>**8.6(6.4)**	56.3(10)<br>57.2(10.5)<br>55.9(10.4)<br>56.9(10.8)<br>**56.6(10.4)**	44.4<br>51.7<br>55.5<br>48.0<br>**47.0**	33.8(6.8)<br>34.3(6.6)<br>34.5(7.1)<br>34.2(7.1)<br>**34.2(6.9)**	8.3(1.0)<br>8.3(1.0)<br>8.2(1.0)<br>8.2(1.0)<br>**8.2(1.0)**	Metformin
**Frias,2023**[9]	NCT03736785	Mexico, Puerto Rico, USA	RCT Phase 2	32 weeks	Degludec: 132<br>BIF: 267<br>**Total: 399**	15.1(8.0)<br>14.6(8.8)<br>**14.7(8.5)**	60.8(10.0)<br>59.9(10.6)<br>**60.1(10.4)**	51.0<br>49.0<br>**49.6**	31.8(5.7)<br>32.5(5.9)<br>**32.2(5.8)**	8.1(0.9)<br>8.1(0.9)<br>**8.1(0.9)**	Insulin +/- Oral Anti-diabetic Drugs
**Gao,2023**[10]	NCT04093752	Australia, China, India, Korea	RCT Phase 3	78 weeks	Tirzepatide 5 mg: 230<br>Tirzepatide 10 mg: 228<br>Tirzepatide 15 mg: 229<br>Glargine: 220<br>**Total: 907**	7.4(5.9)<br>7.9(5.6)<br>7.6(5.6)<br>7.6(5.7)<br>**7.6(5.7)**	53.1(11.2)<br>53.5(11.1)<br>54.3(11.6)<br>55.6(11.4)<br>**54.1(11.3)**	58.35<br>55.3<br>56.3<br>53.6<br>**55.9**	28.1(3.9)<br>27.7(3.8)<br>27.8(3.8)<br>28.0(4.6)<br>**27.9(4.0)**	8.7(0.9)<br>8.7(0.9)<br>8.6(0.9)<br>8.6(0.9)<br>**8.7(0.9)**	Metformin +/- sulfonylurea

**Giorgino,2015**[11]	NCT01075282	Argentina, Australia, Belgium, Brazil, Canada, Croatia, Czech Republic, Greece, Hungary, India, Italy, Korea, Mexico, Poland, Romania, Slovakia, Spain, Sweden, Taiwan,	RCT Phase 3	78 weeks	Dulaglutide 1.5 mg: 273<br>Dulaglutide 0.75 mg:272<br>Glargine: 262<br>**Total: 807**	9.0 (6.0)<br>9.0(6.0)<br>9.0 (6.0)<br>**9.0 (6.0)**	56.0 (10.0)<br>57.0 (9.0)<br>57.0 (9.0)<br>**56.6(9.3)**	53.0<br>50.0<br>51.0<br>**52.0**	31(5)<br>32(5)<br>32(6)<br>**31.6(5.3)**	8.2 (1.0)<br>8.1(1.0)<br>8.1(1.0)<br>**8.1(1.0)**	Metformin or sulfonylurea +/- other oral agents
**Inagaki,2012**[12]	NCT00935532	Japan	RCT Phase 3	26 weeks	Exenatide: 215<br>Glargine: 212<br>**Total: 427**	8.8(6.0)<br>9.2(5.9)<br>**9.0(6.0)**	57.0(10.4)<br>56.4(11.1)<br>**56.7(10.8)**	66.0<br>69.8<br>**67.9**	26.1(4.0)<br>26.1(3.7)<br>**26.1(3.9)**	8.5(0.8)<br>8.5(0.8)<br>**8.5(0.8)**	Oral antidiabetic drugs
**Inagaki,2022**[13]	NCT03861052	Japan	RCT Phase 3	52 weeks	Tirzepatide 5 mg: 159<br>Tirzepatide 5 mg: 158<br>Tirzepatide 5 mg: 160<br>Dulaglutide 0.75 mg: 159<br>**Total: 636**	4.5(2.1-7.5)<br>5.1(2.2-8.4)<br>5.1(2.2-8.)<br>5.0(1.9-8.4)<br>**4.8(2.1-8.3)**	56.8(10.1)<br>56.2(10.3)<br>56.0(10.7)<br>57.5(10.2)<br>**56.6(10.3)**	71.0<br>75.0<br>83.0<br>74.0<br>**76.0**	28.6(5.4)<br>28.0(4.1)<br>28.1(4.4)<br>27.8(3.7)<br>**28.1(4.4)**	8.2(0.9)<br>8.2(0.9)<br>8.2(0.9)<br>8.2(0.9)<br>**8.2(0.9)**	Oral antidiabetic drugs
**Kimura,2023**[14]	UMIN000044264	Japan	RCT Phase 3	24 weeks	Dulaglutide: 53<br>Semaglutide: 54<br>**Total: 107**	13.2(7.0)<br>14.6(7.8)<br>**13.9(7.4)**	62.7(11.4)<br>62.7(10.1)<br>**62.7(10.6)**	55.0<br>56.0<br>**55.5**	29.2(5.9)<br>29.4(5.9)<br>**29.3(5.9)**	8.1(0.6)<br>7.9(0.5)<br>**8.0(0.5)**	Oral antidiabetic drugs
**Lingvay,2023**[15]	NCT04795531	Argentina, Austria, Brazil, Canada, China, Czechia, Denmark, France, Mexico, Puerto Rico, Taiwan, USA	RCT Phase 3	26 weeks	Icodec: 294<br>Degludec: 294<br>**Total: 588**	10.5 (6.5)<br>10.7 (7.2)<br>**10.6(6.8)**	58.0 (10.0)<br>59.0 (10.0)<br>**58.5(10.0)**	62.9<br>62.6<br>**62.7**	29.9(5.2)<br>29.2(5.1)<br>**29.5(5.1)**	8.5(1.1)<br>8.4(1.0)<br>**8.4(1.0)**	Insulin naïve<br>Oral Anti-diabetic Drugs +/- GLP-1 agonist
**Ludvik,2021**[16]	NCT03882970	Argentina, Austria, Greece, Hungary, Italy, Korea, Poland, Puerto Rico, Romania,	RCT Phase 3	52 weeks	Tirzepatide 5 mg: 358<br>Tirzepatide 10 mg: 360<br>Tirzepatide 15 mg: 359<br>Degludec: 360<br>**Total: 1437**	8.5(5.8)<br>8.4(6.6)<br>8.5(6.5)<br>8.1(6.0)<br>**8.4(6.2)**	57.2 (10.1)<br>57.4 (9.7)<br>57.5 (10.2)<br>57.5 (10.1)<br>**47.4(10.0)**	56.0<br>54.0<br>54.0<br>59.0<br>**56.0**	33.6 (5.9)<br>33.4(6.2)<br>33.7(6.1)<br>33.4(6.1)<br>**33.5(6.1)**	8.1(0.8)<br>8.1(0.8)<br>8.2(0.9)<br>8.1(0.9)<br>**8.1(0.9)**	Metformin +/- SGLT-2 inhibitor

		Spain, Taiwan, Ukraine, USA									
**Mathieu,2023**[17]	NCT04880850	Belgium, India, Italy, Japan, Mexico, the Netherlands, Romania, Russia, and the USA	RCT Phase 3	26 weeks	Icodec: 291<br>Glargine: 291<br>**Total: 582**	18.0 (9.1)<br>16.3 (7.7)<br>**17.1(8.4)**	59.7(10.1)<br>59.9(9.9)<br>**59.8(10.0)**	53.0<br>52.0<br>**52.5**	30.5(5.0)<br>30.0(5.0)<br>**30.2(5.0)**	8.2(0.8)<br>8.3(0.9)<br>**8.2(0.8)**	Basal/bolus insulin
**Philis-Tsimikas,2023**[18]	NCT04770532	Bulgaria, Germany, Japan, Korea, Poland, Portugal, South Africa, Ukraine, USA	RCT Phase 3	26 weeks	Icodec: 263<br>Degludec: 263<br>**Total: 526**	16.5(8.4)<br>16.9(7.9)<br>**16.7(8.1)**	62.3(9.8)<br>62.6(8.4)<br>**62.5(9.1)**	62.0<br>53.0<br>**57.0**	29.5(5.2)<br>29.2(4.9)<br>**29.3(5.0)**	8.1(0.7)<br>8.1(0.7)<br>**8.1(0.7)**	Basal insulin +/- oral anti-diabetic drugs
**Pratley, 2018**[19]	NCT02648204	Bulgaria, Croatia, Finland, Germany, Greece, Hong Kong, India, Ireland, Latvia, Lithuania, Portugal, Puerto Rico, Romania, Slovakia, Spain, UK, US	RCT Phase 3	40 weeks	Semaglutide 0.5 mg: 301<br>Dulaglutide 0.75 mg:299<br>Semaglutide 1.0 mg:300<br>Dulaglutide 1.5 mg:299<br>**Total: 1199**	7.7(5.9)<br>7.0(5.5)<br>7.3(5.7)<br>7.6(5.6)<br>**7.4(5.6)**	56(10.9)<br>55(10.4)<br>55(10.6)<br>56(10.6)<br>**56(10.6)**	56.0<br>54.0<br>54.0<br>57.0<br>**55.2**	33.7(7.1)<br>33.6(6.9)<br>33.6(6.5)<br>33.1(6.6)<br>**33.5(6.7)**	8.3(0.9)<br>8.2(0.9)<br>8.2(0.9)<br>8.2(0.9)<br>**8.2(0.9)**	
**Prato,2021**[20]	NCT03730662	Argentina, Australia, Brazil, Canada, Greece, Israel, Mexico, Poland, Puerto Rico, Romania, Russia, Slovakia, Spain, Taiwan, USA	RCT Phase 3	52 weeks	Tirzepatide 5 mg: 329<br>Tirzepatide 10 mg: 328<br>Tirzepatide 15 mg: 338<br>Glargine: 1000<br>**Total: 1995**	9.8(6.7)<br>10.6(7.1)<br>10.4(7.5)<br>10.7(7.5)<br>**10.5(7.5)**	62.9(8.6)<br>63.7(8.7)<br>63.7(8.6)<br>63.8(8.5)<br>**63.6(8.6)**	60.0<br>64.0<br>60.0<br>64.0<br>**62.0**	32.6(6.0)<br>32.8(5.5)<br>32.5(5.0)<br>32.5(5.5)<br>**32.6(5.5)**	8.5(0.8)<br>8.5(0.9)<br>8.5(0.9)<br>8.5(0.8)<br>**8.5(0.8)**	Metformin +/- sulfonylurea, SGLT-2 inhibitors
**Rosenstock, 2020**[21]	NCT03751657	United States, Canada, Czechia, Greece, Poland, Slovakia, Slovenia	RCT Phase 2	26 weeks	Icodec: 125<br>Glargine: 122<br>**Total: 247**	10.5 (8.4)<br>8.8 (6.1)<br>**9.7(7.4)**	59.7(8.2)<br>59.4(9.5)<br>**59.6(8.9)**	56<br>56.6<br>**56.3**	31.1(4.9)<br>31.4(4.4)<br>**31.3(4.6)**	8.0(0.7)<br>7.9(0.6)<br>**8.0(0.6)**	Metformin +/- DPP4 inhibitor

**Rosenstock,2023[22]**	NCT04460885	Croatia, India, Israel, Italy, Japan, Mexico, Poland, Russia, Slovakia, Spain, United Kingdom, and United States	RCT Phase 3	78 weeks	Icodec: 492<br>Glargine: 492<br>**Total: 984**	11.6 (6.7)<br>11.5 (6.8)<br>**11.5(6.7)**	59.1(10.1)<br>58.9(9.9)<br>**59.0(9.9)**	60<br>53.5<br>**56.7**	30.0 (4.8)<br>30.1(5.1)<br>**30.0(4.9)**	8.5(1)<br>8.4(1)<br>**8.4(1)**	Non-insulin agent
**Takahashi, 2023[23]**	jRCTs1011200008	Japan	RCT Phase 3	24 weeks	Dulaglutide: 32<br>Semaglutide: 31<br>**Total: 63**	11.8(6.4)<br>13.7(4.2)<br>**12.7(5.4)**	60.8(49.0-71.8)<br>64.6(54.0-75.0)<br>**62.7(49.0-75.0)**	40.6<br>64.5<br>**52.5**	28.1 (24.5-30.0)<br>26.9 (24.1-30.9)<br>**27.5 (24.1-30.9)**	7.8 (1.0)<br>7.8 (0.8)<br>**7.8 (0.9)**	Oral anti-diabetic medications +/- insulin
**Tuttle,2018[24]**	NCT01621178	Brazil, Hungary, Mexico, Poland, Romania, South Africa, Spain, Ukraine, and the USA	RCT Phase 3	52 weeks	Dulaglutide 1.5 mg: 192<br>Dulaglutide 0.75mg: 190<br>Glargine: 194<br>**Total: 574**	17.6 (8.7)<br>18.0 (8.8)<br>18.7 (8.7)<br>**18.1(8.7)**	64.7(8.8)<br>64.7(8.6)<br>64.3(8.4)<br>**64.5(8.5)**	54.0<br>55.0<br>48.0<br>**52.0**	32.1(4.8)<br>33.0(5.5)<br>32.4(5.3)<br>**32.5(5.2)**	8.6(0.9)<br>8.6(1.1)<br>8.6(1.0)<br>**8.6(1.0)**	Insulin +/- oral antihyperglycemic agent
**Wang,2018[25]**	NCT01648582	China, South Korea, Mexico and Russia	RCT Phase 3	52 weeks	Dulaglutide 1.5 mg: 253<br>Dulaglutide 0.75mg: 252<br>Glargine: 250<br>**Total:755**	7.9 (4.8)<br>8.1 (5.3)<br>8.4 (5.3)<br>**8.1(5.1)**	55.0(9.6)<br>54.5(10.0)<br>55.4(9.2)<br>**55(9.6)**	53.4<br>56.7<br>55.6<br>**55.2**	26.6(3.7)<br>27.0(3.8)<br>26.7(3.5<br>**26.8(3.7)**	8.5(1.2)<br>8.3(1.1)<br>8.3(1.1)<br>**8.4(1.1)**	Metformin +/- sulfonylurea

Supplement S3. Risk of bias assessment of included trials for each outcome

S3.1 Change from baseline in HbA1c (%)

**Study ID**	**Randomization process**	**Deviations from intended intervention**	**Missing outcome data**	**Measurement of the outcome**	**Selection of the reported result**	**Overall bias**
Ahmann,2018	Low risk	Low risk	Low risk	Low risk	Low risk	Low risk
Aroda,2018	Low risk	Low risk	Low risk	Low risk	Low risk	Low risk
Bajaj, 2021	Low risk	Low risk	Some concerns	Low risk	Low risk	Some concerns
Blonde,2015	Low risk	Low risk	Low risk	Low risk	Low risk	Low risk
Bue-Valleskey,2023	Low risk	Low risk	Low risk	Low risk	Some concerns	Some concerns
Diamant,2010	Low risk	Low risk	Low risk	Low risk	Low risk	Low risk
Frias,2021	Low risk	Low risk	Low risk	Low risk	Low risk	Low risk
Frias,2022	Low risk	Low risk	Low risk	Low risk	Low risk	Low risk
Frias,2023	Low risk	Low risk	Low risk	Low risk	Low risk	Low risk

Study ID	Randomization process	Deviations from intended intervention	Missing outcome data	Measurement of the outcome	Selection of the reported result	Overall bias
Gao,2023	Low risk	Low risk	Low risk	Low risk	Low risk	Low risk
Giorgino,2015	Low risk	Low risk	Low risk	Low risk	Low risk	Low risk
Inagaki,2012	Low risk	Low risk	Low risk	Low risk	Some concerns	Some concerns
Inagaki,2022	Low risk	Low risk	Low risk	Low risk	Low risk	Low risk
Kimura,2023	Low risk	Low risk	Some concerns	Low risk	Some concerns	Some concerns
Lingvay,2023	Low risk	Low risk	Low risk	Low risk	Low risk	Low risk
Ludvik,2021	Low risk	Low risk	Low risk	Low risk	Low risk	Low risk
Mathieu,2023	Low risk	Low risk	Low risk	Low risk	Low risk	Low risk
Philis-Tsimikas,2023	Low risk	Low risk	Low risk	Low risk	Low risk	Low risk
Pratley,2018	Low risk	Low risk	Low risk	Low risk	Low risk	Low risk
Prato,2021	Low risk	Low risk	Low risk	Low risk	Low risk	Low risk

Study ID	Randomization process	Deviations from intended intervention	Missing outcome data	Measurement of the outcome	Selection of the reported result	Overall bias
Rosenstock,2020	Low risk	Low risk	Low risk	Low risk	Low risk	Low risk
Rosenstock,2023	Low risk	Low risk	Low risk	Low risk	Low risk	Low risk
Takahashi ,2023	Low risk	Low risk	Some concerns	Low risk	Some concerns	Some concerns
Tuttle,2018	Low risk	Low risk	Low risk	Low risk	Low risk	Low risk
Wang,2018	Low risk	Low risk	Low risk	Low risk	Low risk	Low risk

S3.2 Change from baseline in FPG (mmol/L)

Study ID	Randomization process	Deviations from intended intervention	Missing outcome data	Measurement of the outcome	Selection of the reported result	Overall bias
Ahmann,2018	Low risk	Low risk	Low risk	Low risk	Low risk	Low risk
Aroda,2018	Low risk	Low risk	Low risk	Low risk	Low risk	Low risk
Bajaj, 2021	Low risk	Low risk	Some concerns	Low risk	Low risk	Some concerns
Blonde,2015	Low risk	Low risk	Low risk	Low risk	Low risk	Low risk
Bue-Valleskey,2023	Low risk	Low risk	Low risk	Low risk	Some concerns	Some concerns
Diamant,2010	Low risk	Low risk	Low risk	Low risk	Low risk	Low risk
Frias,2021	Low risk	Low risk	Low risk	Low risk	Low risk	Low risk
Frias,2022	Low risk	Low risk	Low risk	Low risk	Low risk	Low risk
Frias,2023	Low risk	Low risk	Low risk	Low risk	Low risk	Low risk
Gao,2023	Low risk	Low risk	Low risk	Low risk	Low risk	Low risk

Study ID	Randomization process	Deviations from intended intervention	Missing outcome data	Measurement of the outcome	Selection of the reported result	Overall bias
Giorgino,2015	Low risk	Low risk	Low risk	Low risk	Low risk	Low risk
Inagaki,2012	Low risk	Low risk	Low risk	Low risk	Some concerns	Some concerns
Inagaki,2022	Low risk	Low risk	Low risk	Low risk	Low risk	Low risk
Kimura,2023	Low risk	Low risk	Some concerns	Low risk	Some concerns	Some concerns
Lingvay,2023	Low risk	Low risk	Low risk	Low risk	Low risk	Low risk
Ludvik,2021	Low risk	Low risk	Low risk	Low risk	Low risk	Low risk
Mathieu,2023	Low risk	Low risk	Low risk	Low risk	Low risk	Low risk
Philis-Tsimikas,2023	Low risk	Low risk	Low risk	Low risk	Low risk	Low risk
Pratley,2018	Low risk	Low risk	Low risk	Low risk	Low risk	Low risk
Prato,2021	Low risk	Low risk	Low risk	Low risk	Low risk	Low risk
Rosenstock,2020	Low risk	Low risk	Low risk	Low risk	Low risk	Low risk

Study ID	Randomization process	Deviations from intended intervention	Missing outcome data	Measurement of the outcome	Selection of the reported result	Overall bias
Rosenstock,2023	Low risk	Low risk	Low risk	Low risk	Low risk	Low risk
Takahashi ,2023	Low risk	Low risk	Some concerns	Low risk	Some concerns	Some concerns
Tuttle,2018	Low risk	Low risk	Low risk	Low risk	Low risk	Low risk
Wang,2018	Low risk	Low risk	Low risk	Low risk	Low risk	Low risk

S3.3 Change from baseline in BW (kg)

Study ID	Randomization process	Deviations from intended intervention	Missing outcome data	Measurement of the outcome	Selection of the reported result	Overall bias
Ahmann,2018	Low risk	Low risk	Low risk	Low risk	Low risk	Low risk
Aroda,2018	Low risk	Low risk	Low risk	Low risk	Low risk	Low risk
Bajaj, 2021	Low risk	Low risk	Some concerns	Low risk	Low risk	Some concerns
Blonde,2015	Low risk	Low risk	Low risk	Low risk	Low risk	Low risk
Bue-Valleskey,2023	Low risk	Low risk	Low risk	Low risk	Some concerns	Some concerns
Diamant,2010	Low risk	Low risk	Low risk	Low risk	Low risk	Low risk
Frias,2021	Low risk	Low risk	Low risk	Low risk	Low risk	Low risk
Frias,2022	Low risk	Low risk	Low risk	Low risk	Low risk	Low risk
Frias,2023	Low risk	Low risk	Low risk	Low risk	Low risk	Low risk
Gao,2023	Low risk	Low risk	Low risk	Low risk	Low risk	Low risk

Study ID	Randomization process	Deviations from intended intervention	Missing outcome data	Measurement of the outcome	Selection of the reported result	Overall bias
Giorgino,2015	Low risk	Low risk	Low risk	Low risk	Low risk	Low risk
Inagaki,2012	Low risk	Low risk	Low risk	Low risk	Some concerns	Some concerns
Inagaki,2022	Low risk	Low risk	Low risk	Low risk	Low risk	Low risk
Kimura,2023	Low risk	Low risk	Some concerns	Low risk	Some concerns	Some concerns
Lingvay,2023	Low risk	Low risk	Low risk	Low risk	Low risk	Low risk
Ludvik,2021	Low risk	Low risk	Low risk	Low risk	Low risk	Low risk
Mathieu,2023	Low risk	Low risk	Low risk	Low risk	Low risk	Low risk
Philis-Tsimikas,2023	Low risk	Low risk	Low risk	Low risk	Low risk	Low risk
Pratley,2018	Low risk	Low risk	Low risk	Low risk	Low risk	Low risk
Prato,2021	Low risk	Low risk	Low risk	Low risk	Low risk	Low risk
Rosenstock,2020	Low risk	Low risk	Low risk	Low risk	Low risk	Low risk

Study ID	Randomization process	Deviations from intended intervention	Missing outcome data	Measurement of the outcome	Selection of the reported result	Overall bias
Rosenstock,2023	Low risk	Low risk	Low risk	Low risk	Low risk	Low risk
Takahashi ,2023	Low risk	Low risk	Some concerns	Low risk	Some concerns	Some concerns
Tuttle,2018	Low risk	Low risk	Low risk	Low risk	Low risk	Low risk
Wang,2018	Low risk	Low risk	Low risk	Low risk	Low risk	Low risk

S3.4 Change from baseline in TEAE (OR)

Study ID	Randomization process	Deviations from intended intervention	Missing outcome data	Measurement of the outcome	Selection of the reported result	Overall bias
Ahmann,2018	Low risk	Low risk	Low risk	Low risk	Low risk	Low risk
Aroda,2018	Low risk	Low risk	Low risk	Low risk	Low risk	Low risk
Bajaj, 2021	Low risk	Low risk	Some concerns	Low risk	Low risk	Some concerns
Blonde,2015	Low risk	Low risk	Low risk	Low risk	Low risk	Low risk
Bue-Valleskey,2023	Low risk	Low risk	Low risk	Low risk	Some concerns	Some concerns
Diamant,2010	Low risk	Low risk	Low risk	Low risk	Low risk	Low risk
Frias,2021	Low risk	Low risk	Low risk	Low risk	Low risk	Low risk
Frias,2022	Low risk	Low risk	Low risk	Low risk	Low risk	Low risk
Frias,2023	Low risk	Low risk	Low risk	Low risk	Low risk	Low risk

Study ID	Randomization process	Deviations from intended intervention	Missing outcome data	Measurement of the outcome	Selection of the reported result	Overall bias
Gao,2023	Low risk	Low risk	Low risk	Low risk	Low risk	Low risk
Giorgino,2015	Low risk	Low risk	Low risk	Low risk	Low risk	Low risk
Inagaki,2012	Low risk	Low risk	Low risk	Low risk	Some concerns	Some concerns
Inagaki,2022	Low risk	Low risk	Low risk	Low risk	Low risk	Low risk
Kimura,2023	Low risk	Low risk	Some concerns	Low risk	Some concerns	Some concerns
Lingvay,2023	Low risk	Low risk	Low risk	Low risk	Low risk	Low risk
Ludvik,2021	Low risk	Low risk	Low risk	Low risk	Low risk	Low risk
Mathieu,2023	Low risk	Low risk	Low risk	Low risk	Low risk	Low risk
Philis-Tsimikas,2023	Low risk	Low risk	Low risk	Low risk	Low risk	Low risk
Pratley,2018	Low risk	Low risk	Low risk	Low risk	Low risk	Low risk
Prato,2021	Low risk	Low risk	Low risk	Low risk	Low risk	Low risk

Study ID	Randomization process	Deviations from intended intervention	Missing outcome data	Measurement of the outcome	Selection of the reported result	Overall bias
Rosenstock,2020	Low risk	Low risk	Low risk	Low risk	Low risk	Low risk
Rosenstock,2023	Low risk	Low risk	Low risk	Low risk	Low risk	Low risk
Takahashi ,2023	Low risk	Low risk	Some concerns	Low risk	Some concerns	Some concerns
Tuttle,2018	Low risk	Low risk	Low risk	Low risk	Low risk	Low risk
Wang,2018	Low risk	Low risk	Low risk	Low risk	Low risk	Low risk

S3.5 Change from baseline in the risk of hypoglycemia (OR)

Study ID	Randomization process	Deviations from intended intervention	Missing outcome data	Measurement of the outcome	Selection of the reported result	Overall bias
Ahmann,2018	Low risk	Low risk	Low risk	Low risk	Low risk	Low risk
Aroda,2018	Low risk	Low risk	Low risk	Low risk	Low risk	Low risk
Bajaj, 2021	Low risk	Low risk	Some concerns	Low risk	Low risk	Some concerns
Blonde,2015	Low risk	Low risk	Low risk	Low risk	Low risk	Low risk
Bue-Valleskey,2023	Low risk	Low risk	Low risk	Low risk	Some concerns	Some concerns
Diamant,2010	Low risk	Low risk	Low risk	Low risk	Low risk	Low risk
Frias,2021	Low risk	Low risk	Low risk	Low risk	Low risk	Low risk
Frias,2022	Low risk	Low risk	Low risk	Low risk	Low risk	Low risk
Frias,2023	Low risk	Low risk	Low risk	Low risk	Low risk	Low risk
Gao,2023	Low risk	Low risk	Low risk	Low risk	Low risk	Low risk

Study ID	Randomization process	Deviations from intended intervention	Missing outcome data	Measurement of the outcome	Selection of the reported result	Overall bias
Giorgino,2015	Low risk	Low risk	Low risk	Low risk	Low risk	Low risk
Inagaki,2012	Low risk	Low risk	Low risk	Low risk	Some concerns	Some concerns
Inagaki,2022	Low risk	Low risk	Low risk	Low risk	Low risk	Low risk
Kimura,2023	Low risk	Low risk	Some concerns	Low risk	Some concerns	Some concerns
Lingvay,2023	Low risk	Low risk	Low risk	Low risk	Low risk	Low risk
Ludvik,2021	Low risk	Low risk	Low risk	Low risk	Low risk	Low risk
Mathieu,2023	Low risk	Low risk	Low risk	Low risk	Low risk	Low risk
Philis-Tsimikas,2023	Low risk	Low risk	Low risk	Low risk	Low risk	Low risk
Pratley,2018	Low risk	Low risk	Low risk	Low risk	Low risk	Low risk
Prato,2021	Low risk	Low risk	Low risk	Low risk	Low risk	Low risk
Rosenstock,2020	Low risk	Low risk	Low risk	Low risk	Low risk	Low risk

Study ID	Randomization process	Deviations from intended intervention	Missing outcome data	Measurement of the outcome	Selection of the reported result	Overall bias
Rosenstock,2023	Low risk	Low risk	Low risk	Low risk	Low risk	Low risk
Takahashi ,2023	Low risk	Low risk	Some concerns	Low risk	Some concerns	Some concerns
Tuttle,2018	Low risk	Low risk	Low risk	Low risk	Low risk	Low risk
Wang,2018	Low risk	Low risk	Low risk	Low risk	Low risk	Low risk

S3.6 Proportion of serious adverse events (OR)

Study ID	Randomization process	Deviations from intended intervention	Missing outcome data	Measurement of the outcome	Selection of the reported result	Overall bias
Ahmann,2018	Low risk	Low risk	Low risk	Low risk	Low risk	Low risk
Aroda,2018	Low risk	Low risk	Low risk	Low risk	Low risk	Low risk
Bajaj, 2021	Low risk	Low risk	Some concerns	Low risk	Low risk	Some concerns
Blonde,2015	Low risk	Low risk	Low risk	Low risk	Low risk	Low risk
Bue-Valleskey,2023	Low risk	Low risk	Low risk	Low risk	Some concerns	Some concerns
Diamant,2010	Low risk	Low risk	Low risk	Low risk	Low risk	Low risk
Frias,2021	Low risk	Low risk	Low risk	Low risk	Low risk	Low risk
Frias,2022	Low risk	Low risk	Low risk	Low risk	Low risk	Low risk
Frias,2023	Low risk	Low risk	Low risk	Low risk	Low risk	Low risk
Gao,2023	Low risk	Low risk	Low risk	Low risk	Low risk	Low risk

Study ID	Randomization process	Deviations from intended intervention	Missing outcome data	Measurement of the outcome	Selection of the reported result	Overall bias
Giorgino,2015	Low risk	Low risk	Low risk	Low risk	Low risk	Low risk
Inagaki,2012	Low risk	Low risk	Low risk	Low risk	Some concerns	Some concerns
Inagaki,2022	Low risk	Low risk	Low risk	Low risk	Low risk	Low risk
Kimura,2023	Low risk	Low risk	Some concerns	Low risk	Some concerns	Some concerns
Lingvay,2023	Low risk	Low risk	Low risk	Low risk	Low risk	Low risk
Ludvik,2021	Low risk	Low risk	Low risk	Low risk	Low risk	Low risk
Mathieu,2023	Low risk	Low risk	Low risk	Low risk	Low risk	Low risk
Philis-Tsimikas,2023	Low risk	Low risk	Low risk	Low risk	Low risk	Low risk
Pratley,2018	Low risk	Low risk	Low risk	Low risk	Low risk	Low risk
Prato,2021	Low risk	Low risk	Low risk	Low risk	Low risk	Low risk
Rosenstock,2020	Low risk	Low risk	Low risk	Low risk	Low risk	Low risk

Study ID	Randomization process	Deviations from intended intervention	Missing outcome data	Measurement of the outcome	Selection of the reported result	Overall bias
Rosenstock,2023	Low risk	Low risk	Low risk	Low risk	Low risk	Low risk
Takahashi ,2023	Low risk	Low risk	Some concerns	Low risk	Some concerns	Some concerns
Tuttle,2018	Low risk	Low risk	Low risk	Low risk	Low risk	Low risk
Wang,2018	Low risk	Low risk	Low risk	Low risk	Low risk	Low risk

S3.7 Proportion of treatment discontinuation due to adverse events (OR)

Study ID	Randomization process	Deviations from intended intervention	Missing outcome data	Measurement of the outcome	Selection of the reported result	Overall bias
Ahmann,2018	Low risk	Low risk	Low risk	Low risk	Low risk	Low risk
Aroda,2018	Low risk	Low risk	Low risk	Low risk	Low risk	Low risk
Bajaj, 2021	Low risk	Low risk	Some concerns	Low risk	Low risk	Some concerns
Blonde,2015	Low risk	Low risk	Low risk	Low risk	Low risk	Low risk
Bue-Valleskey,2023	Low risk	Low risk	Low risk	Low risk	Some concerns	Some concerns
Diamant,2010	Low risk	Low risk	Low risk	Low risk	Low risk	Low risk
Frias,2021	Low risk	Low risk	Low risk	Low risk	Low risk	Low risk
Frias,2022	Low risk	Low risk	Low risk	Low risk	Low risk	Low risk
Frias,2023	Low risk	Low risk	Low risk	Low risk	Low risk	Low risk
Gao,2023	Low risk	Low risk	Low risk	Low risk	Low risk	Low risk

Study ID	Randomization process	Deviations from intended intervention	Missing outcome data	Measurement of the outcome	Selection of the reported result	Overall bias
Giorgino,2015	Low risk	Low risk	Low risk	Low risk	Low risk	Low risk
Inagaki,2012	Low risk	Low risk	Low risk	Low risk	Some concerns	Some concerns
Inagaki,2022	Low risk	Low risk	Low risk	Low risk	Low risk	Low risk
Kimura,2023	Low risk	Low risk	Some concerns	Low risk	Some concerns	Some concerns
Lingvay,2023	Low risk	Low risk	Low risk	Low risk	Low risk	Low risk
Ludvik,2021	Low risk	Low risk	Low risk	Low risk	Low risk	Low risk
Mathieu,2023	Low risk	Low risk	Low risk	Low risk	Low risk	Low risk
Philis-Tsimikas,2023	Low risk	Low risk	Low risk	Low risk	Low risk	Low risk
Pratley,2018	Low risk	Low risk	Low risk	Low risk	Low risk	Low risk
Prato,2021	Low risk	Low risk	Low risk	Low risk	Low risk	Low risk
Rosenstock,2020	Low risk	Low risk	Low risk	Low risk	Low risk	Low risk

Study ID	Randomization process	Deviations from intended intervention	Missing outcome data	Measurement of the outcome	Selection of the reported result	Overall bias
Rosenstock,2023	Low risk	Low risk	Low risk	Low risk	Low risk	Low risk
Takahashi ,2023	Low risk	Low risk	Some concerns	Low risk	Some concerns	Some concerns
Tuttle,2018	Low risk	Low risk	Low risk	Low risk	Low risk	Low risk
Wang,2018	Low risk	Low risk	Low risk	Low risk	Low risk	Low risk

Supplement S4. Comparison-adjusted funnel plots

S4.1 Comparison adjusted funnel plot for change from baseline in HbA1c

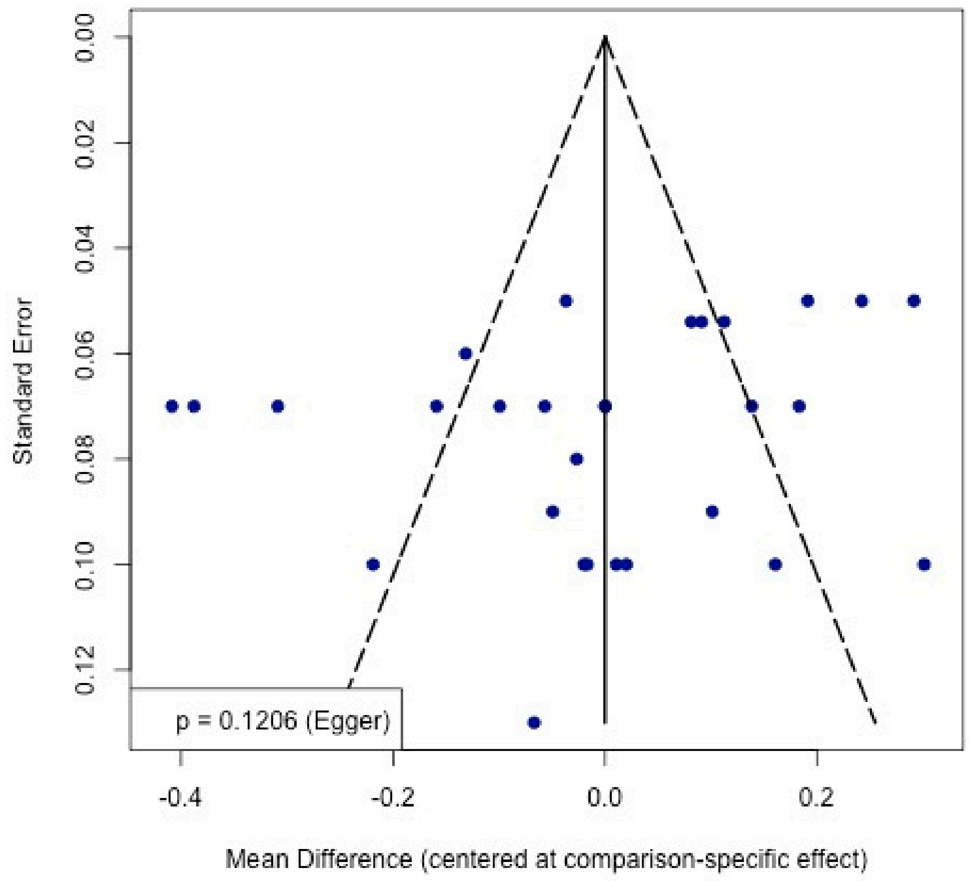

S4.2 Comparison adjusted funnel plot for change from baseline in BW

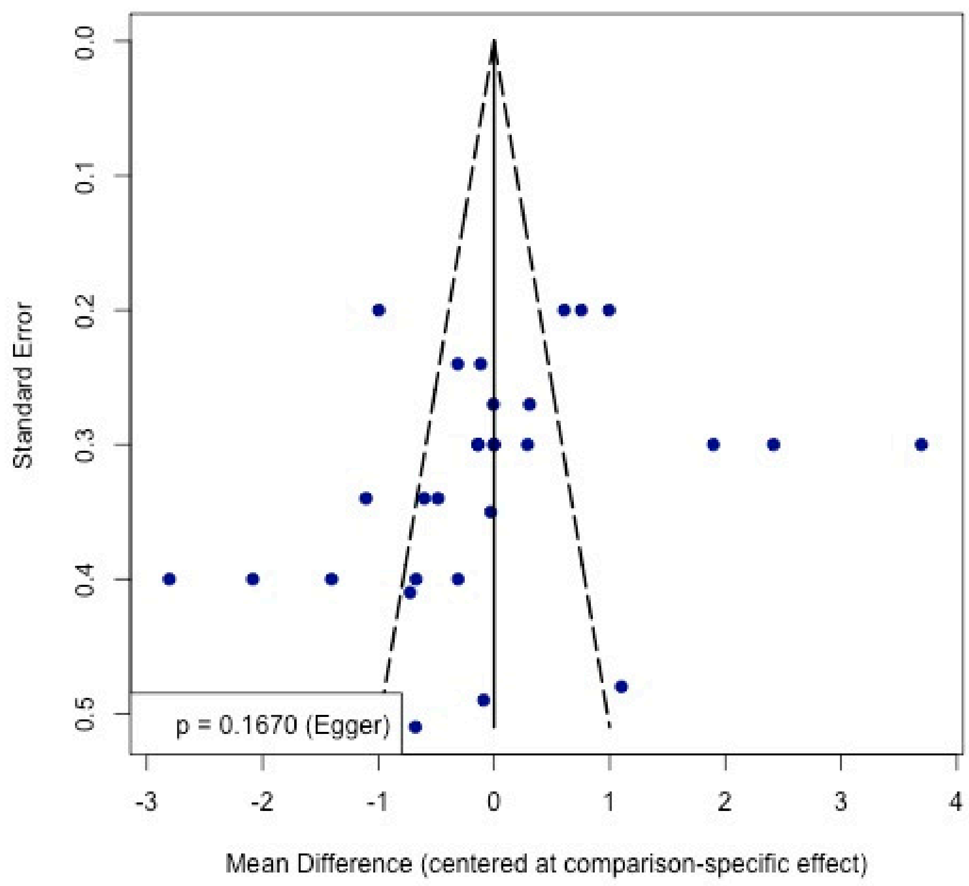

S4.3 Comparison adjusted funnel plot for change from baseline in FPG

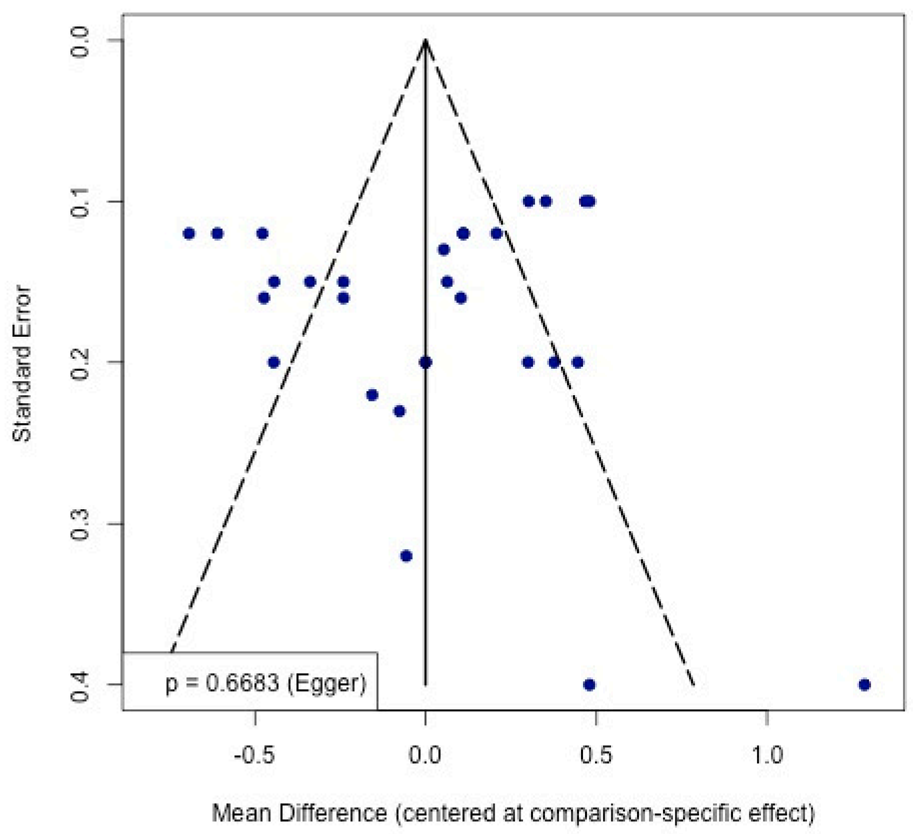

S4.4 Comparison adjusted funnel plot for incidence of TEAE (RR)

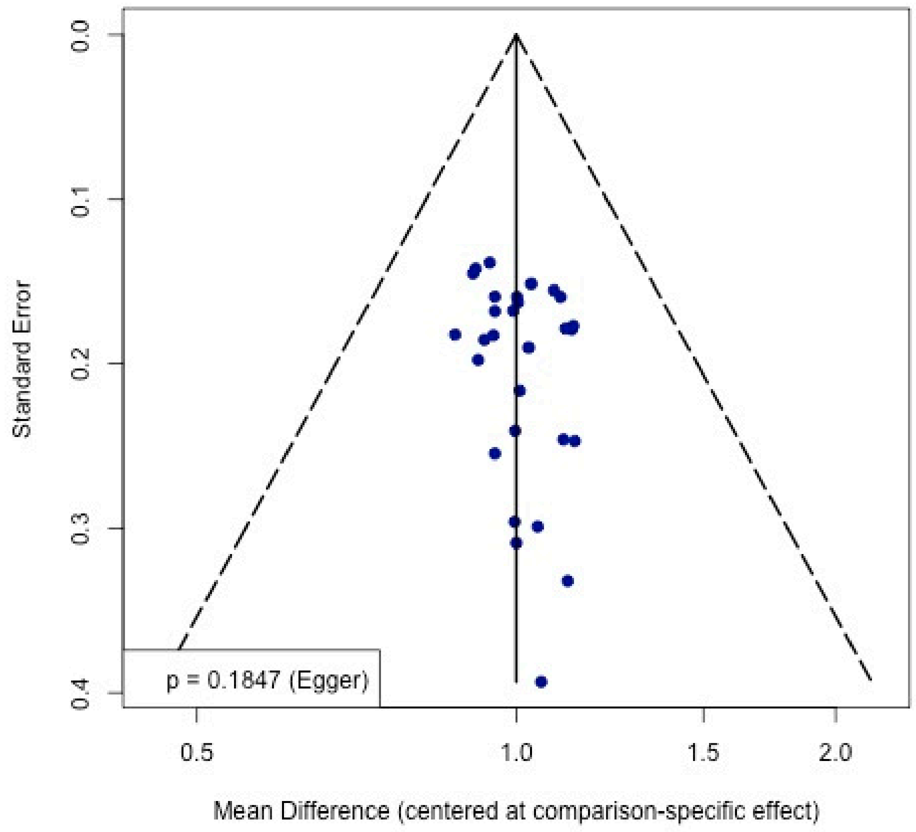

S4.5 Comparison adjusted funnel plot for incidence of pooled hypoglycemia (RR)

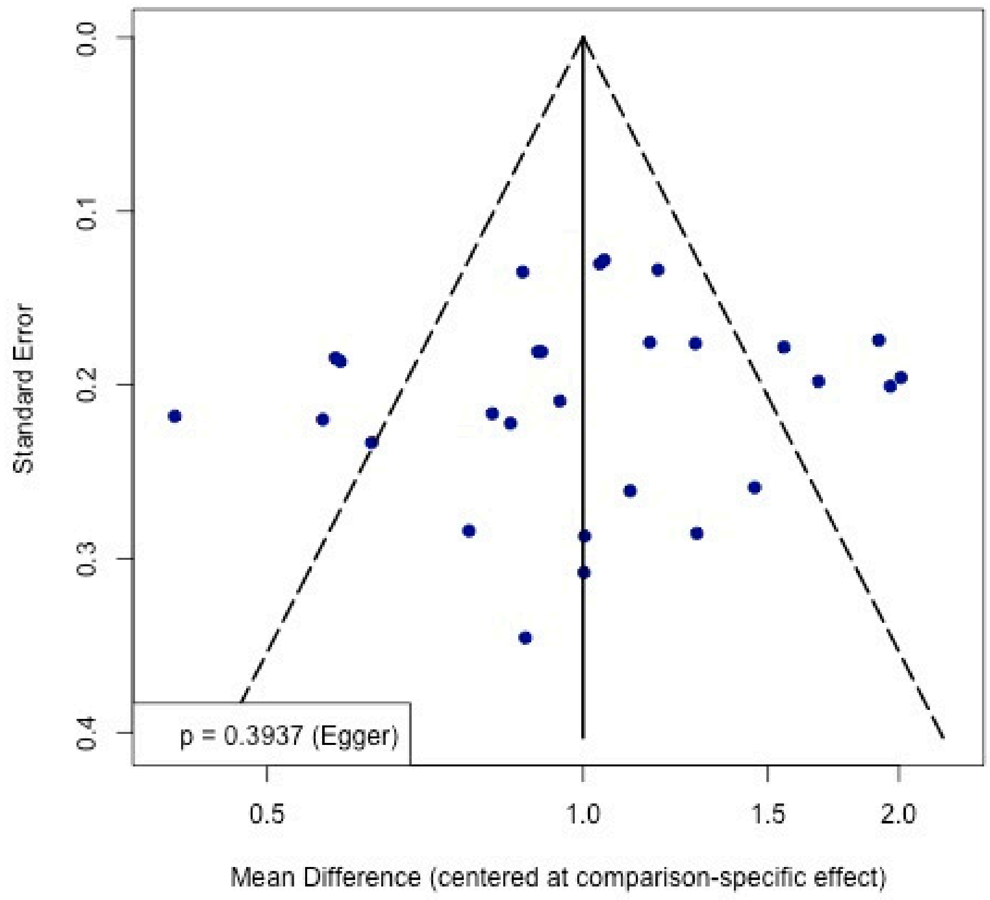

S4.6 Comparison adjusted funnel plot for incidence of treatment discontinuation due to adverse events (RR)

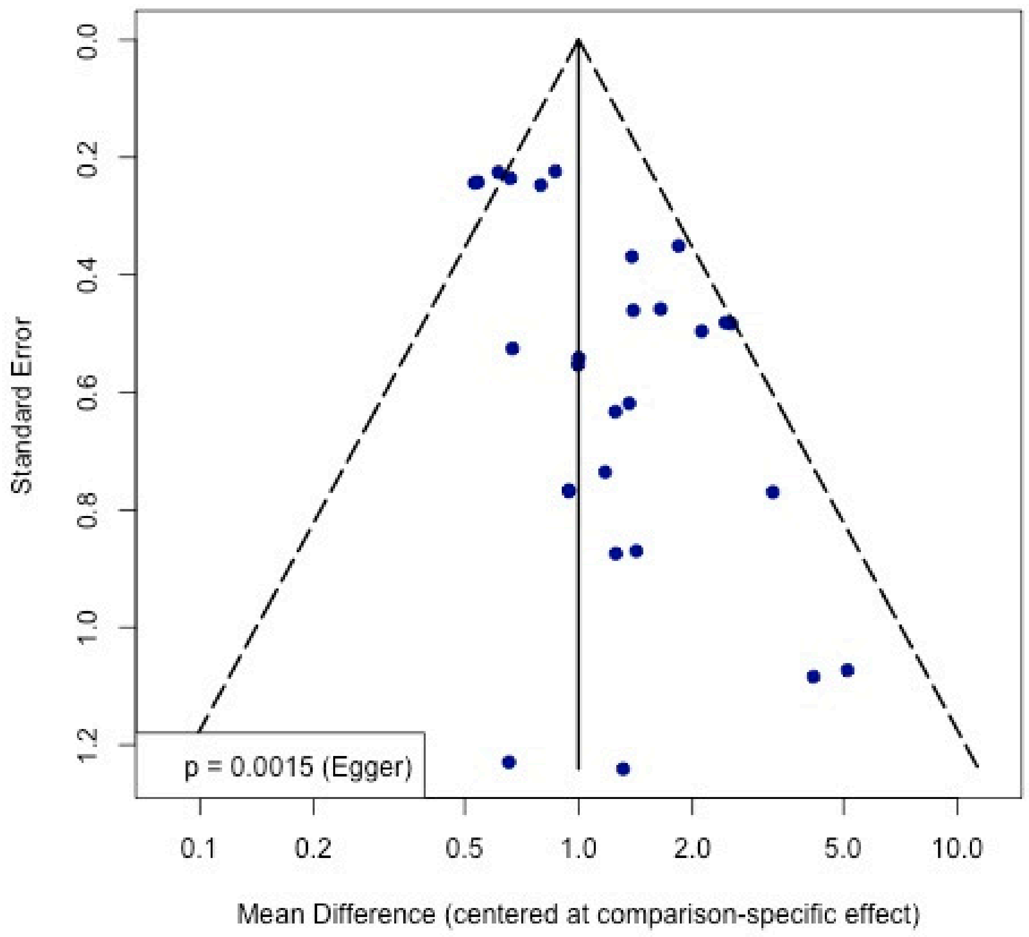

S4.7 Comparison adjusted funnel plot for incidence of serious adverse events (RR)

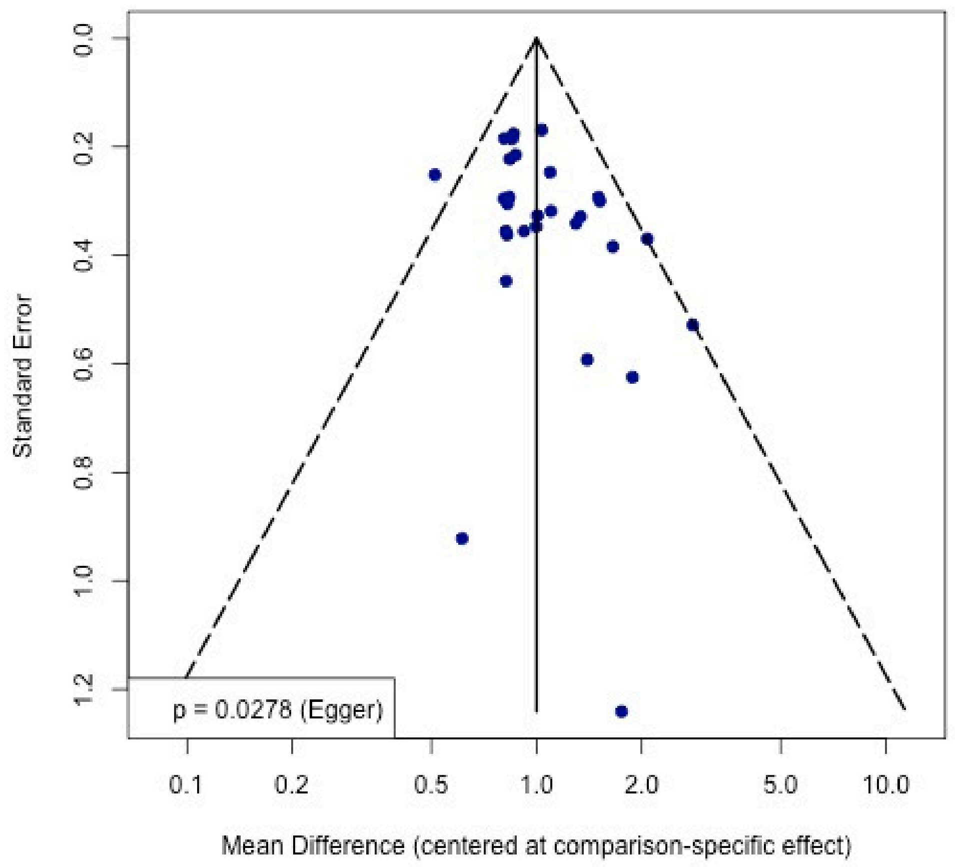

Standard Error
0.0
0.2
0.4
0.6
0.8
1.0
1.2
p = 0.0278 (Egger)
0.1
0.2
0.5
1.0
2.0
5.0
10.0
Mean Difference (centered at comparison-specific effect)

Supplement S5. Pairwise meta-analysis results

S5.1 Pairwise meta-analysis results for change in HbA1c

**Tirzepatide 15 mg**	**-0.13 (-0.18; -0.07)**	**.**	**-0.36 (-0.41; -0.30)**	**-0.45 (-0.57; -0.33)**	**.**	**.**	**.**	**.**	**-1.50 (-1.70; -1.30)**	**-1.18 (-1.25; -1.12)**	**.**
**-0.13 (-0.18; -0.07)**	Tirzepatide 10 mg	.	-0.23 (-0.28; -0.17)	-0.39 (-0.51; -0.27)	.	.	.	.	-1.30 (-1.50; -1.10)	-1.04 (-1.11; -0.98)	.
**-0.26 (-0.40; -0.12)**	-0.13 (-0.27; 0.01)	Semaglutide 2 mg	.	-0.18 (-0.30; -0.06)	.	.	.	.	.	.	.
**-0.36 (-0.41; -0.30)**	-0.23 (-0.28; -0.17)	-0.10 (-0.24; 0.04)	Tirzepatide 5 mg	-0.15 (-0.27; -0.03)	.	.	.	.	-1.10 (-1.30; -0.90)	-0.82 (-0.88; -0.75)	.
**-0.44 (-0.52; -0.36)**	-0.31 (-0.39; -0.23)	-0.18 (-0.30; -0.06)	-0.08 (-0.16; -0.01)	Semaglutide 1 mg	-0.37 (-0.48; -0.27)	-0.40 (-0.56; -0.24)	-0.62 (-0.80; -0.44)	.	-0.70 (-0.86; -0.54)	-0.81 (-0.95; -0.67)	.
**-0.78 (-0.87; -0.68)**	-0.65 (-0.74; -0.55)	-0.52 (-0.66; -0.37)	-0.42 (-0.51; -0.33)	-0.34 (-0.42; -0.25)	Semaglutide 0.5 mg	-0.10 (-0.26; 0.06)	.	.	-0.45 (-0.54; -0.35)	-0.38 (-0.52; -0.24)	.
**-0.89 (-0.98; -0.81)**	-0.76 (-0.85; -0.68)	-0.63 (-0.78; -0.49)	-0.54 (-0.63; -0.45)	-0.45 (-0.54; -0.37)	-0.12 (-0.21; -0.03)	Dulaglutide 1.5 mg	.	.	-0.23 (-0.31; -0.16)	-0.37 (-0.46; -0.29)	.
**-0.94 (-1.03; -0.84)**	-0.81 (-0.90; -0.71)	-0.68 (-0.83; -0.53)	-0.58 (-0.68; -0.49)	-0.50 (-0.59; -0.40)	-0.16 (-0.27; -0.05)	-0.04 (-0.15; 0.06)	Exenatide 2 mg	.	.	-0.32 (-0.40; -0.23)	.
**-1.06 (-1.14; -0.98)**	-0.93 (-1.01; -0.85)	-0.80 (-0.95; -0.65)	-0.70 (-0.79; -0.62)	-0.62 (-0.71; -0.53)	-0.28 (-0.39; -0.18)	-0.17 (-0.26; -0.07)	-0.12 (-0.22; -0.02)	Icodec	.	-0.16 (-0.22; -0.10)	.
**-1.17 (-1.25; -1.09)**	-1.05 (-1.13; -0.97)	-0.92 (-1.06; -0.77)	-0.82 (-0.90; -0.74)	-0.74 (-0.82; -0.65)	-0.40 (-0.48; -0.32)	-0.28 (-0.35; -0.21)	-0.24 (-0.34; -0.14)	-0.11 (-0.20; -0.03)	Dulaglutide 0.75 mg	-0.16 (-0.24; -0.07)	.
**-1.22 (-1.28; -1.17)**	-1.09 (-1.15; -1.04)	-0.96 (-1.10; -0.83)	-0.87 (-0.92; -0.81)	-0.78 (-0.85; -0.71)	-0.45 (-0.53; -0.36)	-0.33 (-0.40; -0.25)	-0.28 (-0.37; -0.20)	-0.16 (-0.22; -0.10)	-0.05 (-0.11; 0.02)	Daily insulin	-0.08 (-0.22; 0.06)
**-1.30 (-1.45; -1.15)**	-1.17 (-1.32; -1.02)	-1.04 (-1.24; -0.85)	-0.95 (-1.10; -0.80)	-0.86 (-1.02; -0.71)	-0.53 (-0.69; -0.36)	-0.41 (-0.56; -0.25)	-0.36 (-0.52; -0.20)	-0.24 (-0.39; -0.09)	-0.13 (-0.28; 0.03)	-0.08 (-0.22; 0.06)	BIF

S5.2 Pairwise meta-analysis results for change in BW

**Tirzepatide 15 mg**	**-1.54 (-1.86; -1.22)**	**-3.91 (-4.23; -3.59)**	**.**	**-5.50 (-6.38; -4.62)**	**.**	**.**	**.**	**-10.10 (-10.88; -9.32)**	**-11.86 (-12.24; -11.47)**	**.**	**.**
**-1.54 (-1.86; -1.22)**	Tirzepatide 10 mg	-2.37 (-2.69; -2.05)	.	-3.60 (-4.48; -2.72)	.	.	.	-7.90 (-8.68; -7.12)	-10.55 (-10.93; -10.16)	.	.
**-3.91 (-4.23; -3.59)**	-2.37 (-2.69; -2.05)	Tirzepatide 5 mg	.	-1.90 (-2.78; -1.02)	.	.	.	-5.20 (-5.98; -4.42)	-8.12 (-8.51; -7.74)	.	.
**-4.68 (-5.57; -3.79)**	-3.14 (-4.03; -2.25)	-0.77 (-1.66; 0.12)	Semaglutide 2 mg	-0.77 (-1.55; 0.01)	.	.	.	.	.	.	.
**-5.45 (-5.87; -5.03)**	-3.91 (-4.33; -3.49)	-1.54 (-1.96; -1.12)	-0.77 (-1.55; 0.01)	Semaglutide 1 mg	-1.81 (-2.22; -1.39)	-3.50 (-4.09; -2.91)	-3.78 (-4.56; -3.00)	-4.20 (-4.79; -3.61)	-6.33 (-6.92; -5.74)	.	.
**-7.22 (-7.65; -6.78)**	-5.68 (-6.11; -5.24)	-3.30 (-3.73; -2.87)	-2.53 (-3.40; -1.67)	-1.76 (-2.12; -1.40)	Semaglutide 0.5 mg	-1.60 (-2.19; -1.01)	.	-2.50 (-2.84; -2.16)	-4.62 (-5.21; -4.03)	.	.
**-8.97 (-9.35; -8.58)**	-7.43 (-7.81; -7.05)	-5.06 (-5.44; -4.67)	-4.29 (-5.14; -3.43)	-3.52 (-3.87; -3.17)	-1.75 (-2.08; -1.43)	Dulaglutide 1.5 mg	.	-0.70 (-0.93; -0.47)	-2.99 (-3.24; -2.74)	.	.
**-8.99 (-9.41; -8.58)**	-7.45 (-7.87; -7.04)	-5.08 (-5.50; -4.67)	-4.31 (-5.19; -3.44)	-3.54 (-3.93; -3.15)	-1.78 (-2.18; -1.38)	-0.03 (-0.37; 0.32)	Exenatide 2 mg	.	-3.00 (-3.28; -2.73)	.	.

**-9.68 (-10.04; -9.31)**	-8.14 (-8.50; -7.77)	-5.76 (-6.13; -5.40)	-4.99 (-5.85; -4.14)	-4.22 (-4.56; -3.89)	-2.46 (-2.75; -2.17)	-0.71 (-0.93; -0.49)	-0.68 (-1.02; -0.35)	Dulaglutide 0.75 mg	-2.30 (-2.55; -2.05)	.	.
**-11.97 (-12.30; -11.64)**	-10.43 (-10.76; -10.10)	-8.06 (-8.38; -7.73)	-7.29 (-8.14; -6.44)	-6.52 (-6.84; -6.19)	-4.75 (-5.07; -4.44)	-3.00 (-3.23; -2.77)	-2.98 (-3.24; -2.71)	-2.29 (-2.51; -2.08)	Daily insulin	-0.56 (-0.86; -0.26)	-0.78 (-1.25; -0.31)
**-12.53 (-12.98; -12.09)**	-10.99 (-11.44; -10.55)	-8.62 (-9.06; -8.18)	-7.85 (-8.75; -6.95)	-7.08 (-7.52; -6.64)	-5.32 (-5.75; -4.88)	-3.56 (-3.94; -3.19)	-3.54 (-3.94; -3.14)	-2.86 (-3.23; -2.49)	-0.56 (-0.86; -0.26)	Icodec	.
**-12.75 (-13.33; -12.18)**	-11.21 (-11.79; -10.64)	-8.84 (-9.41; -8.27)	-8.07 (-9.04; -7.10)	-7.30 (-7.87; -6.73)	-5.54 (-6.10; -4.97)	-3.79 (-4.31; -3.26)	-3.76 (-4.30; -3.22)	-3.08 (-3.59; -2.56)	-0.78 (-1.25; -0.31)	-0.22 (-0.78; 0.34)	BIF

S5.3 Pairwise meta-analysis results for change in FPG

Tirzepatide 15 mg	-0.18 (-0.28; -0.08)	.	-0.51 (-0.60; -0.41)	.	-0.80 (-1.04; -0.56)	-0.51 (-0.64; -0.38)	.	.	.	.	-2.00 (-2.20; -1.80)
-0.17 (-0.26; -0.07)	Tirzepatide 10 mg	.	-0.35 (-0.45; -0.26)	.	-0.70 (-0.94; -0.46)	-0.36 (-0.49; -0.24)	.	.	.	.	-1.80 (-2.00; -1.60)
-0.32 (-0.64; 0.00)	-0.15 (-0.47; 0.17)	Semaglutide 2 mg	.	.	-0.33 (-0.60; -0.06)	.	.	.	.	.	.
-0.51 (-0.61; -0.42)	-0.35 (-0.44; -0.25)	-0.19 (-0.51; 0.12)	Tirzepatide 5 mg	.	-0.40 (-0.64; -0.16)	-0.01 (-0.14; 0.11)	.	.	.	.	-1.40 (-1.60; -1.20)
-0.61 (-0.79; -0.43)	-0.44 (-0.62; -0.26)	-0.29 (-0.64; 0.06)	-0.10 (-0.27; 0.08)	Icodec	.	-0.04 (-0.18; 0.10)	.	.	.	.	.

-0.65 (-0.81; -0.49)	-0.48 (-0.64; -0.32)	-0.33 (-0.60; -0.06)	-0.14 (-0.30; 0.02)	-0.04 (-0.25; 0.17)	Semaglutide 1 mg	-0.61 (-1.00; -0.22)	-0.84 (-1.23; -0.45)	-0.64 (-0.92; -0.37)	-0.60 (-0.99; -0.21)	.	-0.90 (-1.29; -0.51)
-0.65 (-0.75; -0.54)	-0.48 (-0.59; -0.37)	-0.33 (-0.65; -0.01)	-0.13 (-0.24; -0.03)	-0.04 (-0.18; 0.10)	0.00 (-0.16; 0.16)	Daily insulin	-0.16 (-0.43; 0.12)	-0.08 (-0.47; 0.31)	-0.53 (-0.72; -0.35)	-0.70 (-0.87; -0.54)	-1.04 (-1.22; -0.85)
-1.03 (-1.28; -0.78)	-0.86 (-1.11; -0.62)	-0.71 (-1.08; -0.34)	-0.52 (-0.77; -0.27)	-0.42 (-0.69; -0.15)	-0.38 (-0.63; -0.13)	-0.38 (-0.62; -0.15)	Exenatide 2 mg	.	.	.	.
-1.17 (-1.38; -0.96)	-1.00 (-1.21; -0.79)	-0.85 (-1.20; -0.50)	-0.66 (-0.87; -0.45)	-0.56 (-0.81; -0.32)	-0.52 (-0.74; -0.31)	-0.52 (-0.72; -0.32)	-0.14 (-0.44; 0.16)	Semaglutide 0.5 mg	-0.00 (-0.39; 0.39)	.	-0.55 (-0.78; -0.32)
-1.26 (-1.43; -1.08)	-1.09 (-1.26; -0.91)	-0.94 (-1.28; -0.60)	-0.74 (-0.92; -0.57)	-0.65 (-0.86; -0.44)	-0.61 (-0.81; -0.41)	-0.61 (-0.77; -0.45)	-0.23 (-0.50; 0.05)	-0.09 (-0.31; 0.14)	Dulaglutide 1.5 mg	.	-0.47 (-0.63; -0.30)
-1.35 (-1.54; -1.15)	-1.18 (-1.38; -0.99)	-1.03 (-1.39; -0.68)	-0.84 (-1.03; -0.64)	-0.74 (-0.96; -0.53)	-0.70 (-0.93; -0.47)	-0.70 (-0.87; -0.54)	-0.32 (-0.60; -0.04)	-0.18 (-0.44; 0.08)	-0.09 (-0.32; 0.13)	BIF	.
-1.81 (-1.94; -1.68)	-1.64 (-1.78; -1.51)	-1.49 (-1.82; -1.17)	-1.30 (-1.43; -1.17)	-1.20 (-1.39; -1.01)	-1.16 (-1.33; -0.99)	-1.16 (-1.29; -1.04)	-0.78 (-1.04; -0.52)	-0.64 (-0.83; -0.45)	-0.55 (-0.71; -0.40)	-0.46 (-0.66; -0.26)	Dulaglutide 0.75 mg

Supplement S6. Certainty of the effect estimates

S6.1 Certainty in effect estimates for change in HbA1c

Comparison	Number of studies	Within-study bias	Reporting bias	Indirectness	Imprecision	Heterogeneity	Incoherence	Confidence rating	Reason(s) for downgrading
BIF:Degludec	2	Some concerns	Low risk	No concerns	Some concerns	Some concerns	No concerns	Moderate	["Within-study bias","Imprecision","Heterogeneity"]
Degludec:Icodec	2	No concerns	Low risk	No concerns	Some concerns	Some concerns	Major concerns	Low	["Imprecision","Heterogeneity","Incoherence"]
Degludec:Tirzepatide 10 mg	1	No concerns	Low risk	No concerns	No concerns	No concerns	No concerns	High	[]
Degludec:Tirzepatide 15 mg	1	No concerns	Low risk	No concerns	No concerns	No concerns	No concerns	High	[]
Degludec:Tirzepatide 5 mg	1	No concerns	Low risk	No concerns	No concerns	No concerns	No concerns	High	[]
Dulaglutide 0.75 mg:Dulaglutide 1.5 mg	5	No concerns	Low risk	No concerns	No concerns	Some concerns	Some concerns	Moderate	["Heterogeneity","Incoherence"]
Dulaglutide 0.75 mg:Glargine	4	No concerns	Low risk	No concerns	No concerns	Major concerns	No concerns	Low	["Heterogeneity"]

Dulaglutide 0.75 mg:Semaglutide 0.5 mg	3	No concerns	Low risk	No concerns	No concerns	No concerns	No concerns	High	[]
Dulaglutide 0.75 mg:Semaglutide 1 mg	1	No concerns	Low risk	No concerns	No concerns	No concerns	No concerns	High	[]
Dulaglutide 0.75 mg:Tirzepatide 10 mg	1	No concerns	Low risk	No concerns	No concerns	No concerns	No concerns	High	[]
Dulaglutide 0.75 mg:Tirzepatide 15 mg	1	No concerns	Low risk	No concerns	No concerns	No concerns	No concerns	High	[]
Dulaglutide 0.75 mg:Tirzepatide 5 mg	1	No concerns	Low risk	No concerns	No concerns	No concerns	No concerns	High	[]
Dulaglutide 1.5 mg:Glargine	4	No concerns	Low risk	No concerns	No concerns	No concerns	No concerns	High	[]
Dulaglutide 1.5 mg:Semaglutide 0.5 mg	1	No concerns	Low risk	No concerns	Some concerns	No concerns	No concerns	Moderate	["Imprecision"]
Dulaglutide 1.5 mg:Semaglutide 1 mg	1	No concerns	Low risk	No concerns	No concerns	No concerns	No concerns	High	[]
Exenatide 2 mg:Glargine	2	No concerns	Low risk	No concerns	No concerns	Some concerns	No concerns	Moderate	["Heterogeneity"]
Exenatide 2 mg:Semaglutide 1 mg	1	No concerns	Low risk	No concerns	No concerns	No concerns	No concerns	High	[]
Glargine:Icodec	4	No concerns	Low risk	No concerns	No concerns	Some concerns	Some concerns	Moderate	["Heterogeneity","Incoherence"]

Glargine:Semaglutide 0.5 mg	1	No concerns	Low risk	No concerns	No concerns	No concerns	No concerns	High	[]
Glargine:Semaglutide 1 mg	1	No concerns	Low risk	No concerns	No concerns	No concerns	No concerns	High	[]
Glargine:Tirzepatide 10 mg	2	No concerns	Low risk	No concerns	No concerns	No concerns	No concerns	High	[]
Glargine:Tirzepatide 15 mg	2	No concerns	Low risk	No concerns	No concerns	No concerns	No concerns	High	[]
Glargine:Tirzepatide 5 mg	2	No concerns	Low risk	No concerns	No concerns	No concerns	No concerns	High	[]
Semaglutide 0.5 mg:Semaglutide 1 mg	2	No concerns	Low risk	No concerns	No concerns	No concerns	No concerns	High	[]
Semaglutide 1 mg:Semaglutide 2 mg	1	No concerns	Low risk	No concerns	Some concerns	Some concerns	No concerns	Moderate	["Imprecision","Heterogeneity"]
Semaglutide 1 mg:Tirzepatide 10 mg	1	No concerns	Low risk	No concerns	No concerns	No concerns	No concerns	High	[]
Semaglutide 1 mg:Tirzepatide 15 mg	1	No concerns	Low risk	No concerns	No concerns	No concerns	No concerns	High	[]
Semaglutide 1 mg:Tirzepatide 5 mg	1	No concerns	Low risk	No concerns	Some concerns	No concerns	No concerns	Moderate	["Imprecision"]
Tirzepatide 10 mg:Tirzepatide 15 mg	5	No concerns	Low risk	No concerns	Some concerns	No concerns	No concerns	Moderate	["Imprecision"]

Tirzepatide 10 mg:Tirzepatide 5 mg	5	No concerns	Low risk	No concerns	No concerns	Some concerns	No concerns	Moderate	["Heterogeneity"]
Tirzepatide 15 mg:Tirzepatide 5 mg	5	No concerns	Low risk	No concerns	No concerns	No concerns	No concerns	High	[]
BIF:Dulaglutide 0.75 mg	0	No concerns	Low risk	No concerns	Major concerns	No concerns	No concerns	Low	["Imprecision"]
BIF:Dulaglutide 1.5 mg	0	No concerns	Low risk	No concerns	Some concerns	No concerns	No concerns	Moderate	["Imprecision"]
BIF:Exenatide 2 mg	0	No concerns	Low risk	No concerns	Some concerns	Some concerns	No concerns	Moderate	["Imprecision","Heterogeneity"]
BIF:Glargine	0	No concerns	Low risk	No concerns	Major concerns	No concerns	No concerns	Low	["Imprecision"]
BIF:Icodec	0	No concerns	Low risk	No concerns	Some concerns	Some concerns	No concerns	Moderate	["Imprecision","Heterogeneity"]
BIF:Semaglutide 0.5 mg	0	No concerns	Low risk	No concerns	No concerns	Some concerns	No concerns	Moderate	["Heterogeneity"]
BIF:Semaglutide 1 mg	0	No concerns	Low risk	No concerns	No concerns	No concerns	No concerns	High	[]
BIF:Semaglutide 2 mg	0	No concerns	Low risk	No concerns	No concerns	No concerns	No concerns	High	[]
BIF:Tirzepatide 10 mg	0	No concerns	Low risk	No concerns	No concerns	No concerns	No concerns	High	[]

BIF:Tirzepatide 15 mg	0	No concerns	Low risk	No concerns	No concerns	No concerns	No concerns	High	[]
BIF:Tirzepatide 5 mg	0	No concerns	Low risk	No concerns	No concerns	No concerns	No concerns	High	[]
Degludec:Dulaglutide 0.75 mg	0	No concerns	Low risk	No concerns	Some concerns	Some concerns	No concerns	Moderate	["Imprecision","Heterogeneity"]
Degludec:Dulaglutide 1.5 mg	0	No concerns	Low risk	No concerns	Some concerns	Some concerns	No concerns	Moderate	["Imprecision","Heterogeneity"]
Degludec:Exenatide 2 mg	0	No concerns	Low risk	No concerns	Some concerns	Some concerns	No concerns	Moderate	["Imprecision","Heterogeneity"]
Degludec:Glargine	0	No concerns	Low risk	No concerns	Some concerns	No concerns	No concerns	Moderate	["Imprecision"]
Degludec:Semaglutide 0.5 mg	0	No concerns	Low risk	No concerns	No concerns	Some concerns	No concerns	Moderate	["Heterogeneity"]
Degludec:Semaglutide 1 mg	0	No concerns	Low risk	No concerns	No concerns	No concerns	No concerns	High	[]
Degludec:Semaglutide 2 mg	0	No concerns	Low risk	No concerns	No concerns	No concerns	No concerns	High	[]
Dulaglutide 0.75 mg:Exenatide 2 mg	0	No concerns	Low risk	No concerns	Some concerns	No concerns	No concerns	Moderate	["Imprecision"]
Dulaglutide 0.75 mg:Icodec	0	No concerns	Low risk	No concerns	Some concerns	No concerns	No concerns	Moderate	["Imprecision"]

Dulaglutide 0.75 mg:Semaglutide 2 mg	0	No concerns	Low risk	No concerns	No concerns	No concerns	No concerns	High	[]
Dulaglutide 1.5 mg:Exenatide 2 mg	0	No concerns	Low risk	No concerns	Some concerns	Some concerns	No concerns	Moderate	["Imprecision","Heterogeneity"]
Dulaglutide 1.5 mg:Icodec	0	No concerns	Low risk	No concerns	Some concerns	Some concerns	No concerns	Moderate	["Imprecision","Heterogeneity"]
Dulaglutide 1.5 mg:Semaglutide 2 mg	0	No concerns	Low risk	No concerns	No concerns	No concerns	No concerns	High	[]
Dulaglutide 1.5 mg:Tirzepatide 10 mg	0	No concerns	Low risk	No concerns	No concerns	No concerns	No concerns	High	[]
Dulaglutide 1.5 mg:Tirzepatide 15 mg	0	No concerns	Low risk	No concerns	No concerns	No concerns	No concerns	High	[]
Dulaglutide 1.5 mg:Tirzepatide 5 mg	0	No concerns	Low risk	No concerns	No concerns	No concerns	No concerns	High	[]
Exenatide 2 mg:Icodec	0	No concerns	Low risk	No concerns	Some concerns	Some concerns	No concerns	Moderate	["Imprecision","Heterogeneity"]
Exenatide 2 mg:Semaglutide 0.5 mg	0	No concerns	Low risk	No concerns	Some concerns	No concerns	No concerns	Moderate	["Imprecision"]
Exenatide 2 mg:Semaglutide 2 mg	0	No concerns	Low risk	No concerns	No concerns	No concerns	No concerns	High	[]
Exenatide 2 mg:Tirzepatide 10 mg	0	No concerns	Low risk	No concerns	No concerns	No concerns	No concerns	High	[]

Exenatide 2 mg:Tirzepatide 15 mg	0	No concerns	Low risk	No concerns	No concerns	No concerns	No concerns	High	[]
Exenatide 2 mg:Tirzepatide 5 mg	0	No concerns	Low risk	No concerns	No concerns	No concerns	No concerns	High	[]
Glargine:Semaglutide 2 mg	0	No concerns	Low risk	No concerns	No concerns	No concerns	No concerns	High	[]
Icodec:Semaglutide 0.5 mg	0	No concerns	Low risk	No concerns	No concerns	Some concerns	No concerns	Moderate	["Heterogeneity"]
Icodec:Semaglutide 1 mg	0	No concerns	Low risk	No concerns	No concerns	No concerns	No concerns	High	[]
Icodec:Semaglutide 2 mg	0	No concerns	Low risk	No concerns	No concerns	No concerns	No concerns	High	[]
Icodec:Tirzepatide 10 mg	0	No concerns	Low risk	No concerns	No concerns	No concerns	No concerns	High	[]
Icodec:Tirzepatide 15 mg	0	No concerns	Low risk	No concerns	No concerns	No concerns	No concerns	High	[]
Icodec:Tirzepatide 5 mg	0	No concerns	Low risk	No concerns	No concerns	No concerns	No concerns	High	[]
Semaglutide 0.5 mg:Semaglutide 2 mg	0	No concerns	Low risk	No concerns	No concerns	No concerns	No concerns	High	[]
Semaglutide 0.5 mg:Tirzepatide 10 mg	0	No concerns	Low risk	No concerns	No concerns	No concerns	No concerns	High	[]

Semaglutide 0.5 mg:Tirzepatide 15 mg	0	No concerns	Low risk	No concerns	No concerns	No concerns	No concerns	High	[]
Semaglutide 0.5 mg:Tirzepatide 5 mg	0	No concerns	Low risk	No concerns	No concerns	No concerns	No concerns	High	[]
Semaglutide 2 mg:Tirzepatide 10 mg	0	No concerns	Low risk	No concerns	Some concerns	Some concerns	No concerns	Moderate	["Imprecision","Heterogeneity"]
Semaglutide 2 mg:Tirzepatide 15 mg	0	No concerns	Low risk	No concerns	Some concerns	No concerns	No concerns	Moderate	["Imprecision"]
Semaglutide 2 mg:Tirzepatide 5 mg	0	No concerns	Low risk	No concerns	Major concerns	No concerns	No concerns	Low	["Imprecision"]

S6.2 Certainty in effect estimates for change in FPG

Comparison	Number of studies	Within-study bias	Reporting bias	Indirectness	Imprecision	Heterogeneity	Incoherence	Confidence rating	Reason(s) for downgrading
BIF:Degludec	2	No concerns	Low risk	No concerns	No concerns	Some concerns	No concerns	Moderate	["Heterogeneity"]
Degludec:Icodec	2	No concerns	Low risk	No concerns	Some concerns	Some concerns	Some concerns	Moderate	["Imprecision","Heterogeneity","Incoherence"]
Degludec:Tirzepatide 10 mg	1	No concerns	Low risk	No concerns	Some concerns	Some concerns	No concerns	Moderate	["Imprecision","Heterogeneity"]
Degludec:Tirzepatide 15 mg	1	No concerns	Low risk	No concerns	Some concerns	Some concerns	No concerns	Moderate	["Imprecision","Heterogeneity"]
Degludec:Tirzepatide 5 mg	1	No concerns	Low risk	No concerns	Major concerns	No concerns	No concerns	Low	["Imprecision"]
Dulaglutide 0.75 mg:Dulaglutide 1.5 mg	5	No concerns	Low risk	No concerns	No concerns	Some concerns	No concerns	Moderate	["Heterogeneity"]
Dulaglutide 0.75 mg:Glargine	4	No concerns	Low risk	No concerns	No concerns	No concerns	No concerns	High	[]
Dulaglutide 0.75 mg:Semaglutide 0.5 mg	3	No concerns	Low risk	No concerns	No concerns	No concerns	No concerns	High	[]
Dulaglutide 0.75 mg:Semaglutide 1 mg	1	No concerns	Low risk	No concerns	No concerns	No concerns	No concerns	High	[]
Dulaglutide 0.75 mg:Tirzepatide 10 mg	1	No concerns	Low risk	No concerns	No concerns	No concerns	No concerns	High	[]
Dulaglutide 0.75 mg:Tirzepatide 15 mg	1	No concerns	Low risk	No concerns	No concerns	No concerns	No concerns	High	[]
Dulaglutide 0.75 mg:Tirzepatide 5 mg	1	No concerns	Low risk	No concerns	No concerns	No concerns	No concerns	High	[]
Dulaglutide 1.5 mg:Glargine	4	No concerns	Low risk	No concerns	No concerns	No concerns	No concerns	High	[]

Dulaglutide 1.5 mg:Semaglutide 0.5 mg	1	No concerns	Low risk	No concerns	Some concerns	Some concerns	No concerns	Moderate	["Imprecision","Heterogeneity"]
Dulaglutide 1.5 mg:Semaglutide 1 mg	1	No concerns	Low risk	No concerns	No concerns	No concerns	No concerns	High	[]
Exenatide 2 mg:Glargine	2	No concerns	Low risk	No concerns	Some concerns	Some concerns	No concerns	Moderate	["Imprecision","Heterogeneity"]
Exenatide 2 mg:Semaglutide 1 mg	1	No concerns	Low risk	No concerns	No concerns	Some concerns	No concerns	Moderate	["Heterogeneity"]
Glargine:Icodec	4	No concerns	Low risk	No concerns	Some concerns	Some concerns	Some concerns	Moderate	["Imprecision","Heterogeneity","Incoherence"]
Glargine:Semaglutide 0.5 mg	1	No concerns	Low risk	No concerns	No concerns	Some concerns	No concerns	Moderate	["Heterogeneity"]
Glargine:Semaglutide 1 mg	1	No concerns	Low risk	No concerns	Some concerns	Some concerns	No concerns	Moderate	["Imprecision","Heterogeneity"]
Glargine:Tirzepatide 10 mg	2	No concerns	Low risk	No concerns	No concerns	No concerns	No concerns	High	[]
Glargine:Tirzepatide 15 mg	2	No concerns	Low risk	No concerns	No concerns	No concerns	No concerns	High	[]
Glargine:Tirzepatide 5 mg	2	No concerns	Low risk	No concerns	No concerns	Major concerns	No concerns	Low	["Heterogeneity"]
Semaglutide 0.5 mg:Semaglutide 1 mg	2	No concerns	Low risk	No concerns	No concerns	Some concerns	No concerns	Moderate	["Heterogeneity"]
Semaglutide 1 mg:Semaglutide 2 mg	1	No concerns	Low risk	No concerns	Major concerns	No concerns	No concerns	Low	["Imprecision"]
Semaglutide 1 mg:Tirzepatide 10 mg	1	No concerns	Low risk	No concerns	No concerns	Some concerns	No concerns	Moderate	["Heterogeneity"]
Semaglutide 1 mg:Tirzepatide 15 mg	1	No concerns	Low risk	No concerns	No concerns	No concerns	No concerns	High	[]
Semaglutide 1 mg:Tirzepatide 5 mg	1	No concerns	Low risk	No concerns	Some concerns	Some concerns	No concerns	Moderate	["Imprecision","Heterogeneity"]
Tirzepatide 10 mg:Tirzepatide 15 mg	5	No concerns	Low risk	No concerns	Some concerns	Some concerns	No concerns	Moderate	["Imprecision","Heterogeneity"]

Tirzepatide 10 mg:Tirzepatide 5 mg	5	No concerns	Low risk	No concerns	No concerns	Major concerns	No concerns	Low	["Heterogeneity"]
Tirzepatide 15 mg:Tirzepatide 5 mg	5	No concerns	Low risk	No concerns	No concerns	Some concerns	No concerns	Moderate	["Heterogeneity"]
BIF:Dulaglutide 0.75 mg	0	No concerns	Low risk	No concerns	No concerns	No concerns	No concerns	High	[]
BIF:Dulaglutide 1.5 mg	0	No concerns	Low risk	No concerns	Some concerns	Some concerns	No concerns	Moderate	["Imprecision","Heterogeneity"]
BIF:Exenatide 2 mg	0	No concerns	Low risk	No concerns	Major concerns	No concerns	No concerns	Low	["Imprecision"]
BIF:Glargine	0	No concerns	Low risk	No concerns	Major concerns	No concerns	No concerns	Low	["Imprecision"]
BIF:Icodec	0	No concerns	Low risk	No concerns	Some concerns	Some concerns	No concerns	Moderate	["Imprecision","Heterogeneity"]
BIF:Semaglutide 0.5 mg	0	No concerns	Low risk	No concerns	Major concerns	No concerns	No concerns	Low	["Imprecision"]
BIF:Semaglutide 1 mg	0	No concerns	Low risk	No concerns	Major concerns	No concerns	No concerns	Low	["Imprecision"]
BIF:Semaglutide 2 mg	0	No concerns	Low risk	No concerns	Some concerns	Some concerns	No concerns	Moderate	["Imprecision","Heterogeneity"]
BIF:Tirzepatide 10 mg	0	No concerns	Low risk	No concerns	No concerns	No concerns	No concerns	High	[]
BIF:Tirzepatide 15 mg	0	No concerns	Low risk	No concerns	No concerns	No concerns	No concerns	High	[]
BIF:Tirzepatide 5 mg	0	No concerns	Low risk	No concerns	Some concerns	Some concerns	No concerns	Moderate	["Imprecision","Heterogeneity"]
Degludec:Dulaglutide 0.75 mg	0	No concerns	Low risk	No concerns	No concerns	No concerns	No concerns	High	[]
Degludec:Dulaglutide 1.5 mg	0	No concerns	Low risk	No concerns	No concerns	No concerns	No concerns	High	[]
Degludec:Exenatide 2 mg	0	No concerns	Low risk	No concerns	No concerns	No concerns	No concerns	High	[]

Degludec:Glargine	0	No concerns	Low risk	No concerns	No concerns	Some concerns	No concerns	Moderate	["Heterogeneity"]
Degludec:Semaglutide 0.5 mg	0	No concerns	Low risk	No concerns	No concerns	No concerns	No concerns	High	[]
Degludec:Semaglutide 1 mg	0	No concerns	Low risk	No concerns	Some concerns	Some concerns	No concerns	Moderate	["Imprecision","Heterogeneity"]
Degludec:Semaglutide 2 mg	0	No concerns	Low risk	No concerns	Major concerns	No concerns	No concerns	Low	["Imprecision"]
Dulaglutide 0.75 mg:Exenatide 2 mg	0	No concerns	Low risk	No concerns	No concerns	No concerns	No concerns	High	[]
Dulaglutide 0.75 mg:Icodec	0	No concerns	Low risk	No concerns	No concerns	No concerns	No concerns	High	[]
Dulaglutide 0.75 mg:Semaglutide 2 mg	0	No concerns	Low risk	No concerns	No concerns	No concerns	No concerns	High	[]
Dulaglutide 1.5 mg:Exenatide 2 mg	0	No concerns	Low risk	No concerns	Some concerns	Some concerns	No concerns	Moderate	["Imprecision","Heterogeneity"]
Dulaglutide 1.5 mg:Icodec	0	No concerns	Low risk	No concerns	No concerns	No concerns	No concerns	High	[]
Dulaglutide 1.5 mg:Semaglutide 2 mg	0	No concerns	Low risk	No concerns	No concerns	No concerns	No concerns	High	[]
Dulaglutide 1.5 mg:Tirzepatide 10 mg	0	No concerns	Low risk	No concerns	No concerns	No concerns	No concerns	High	[]
Dulaglutide 1.5 mg:Tirzepatide 15 mg	0	No concerns	Low risk	No concerns	No concerns	No concerns	No concerns	High	[]
Dulaglutide 1.5 mg:Tirzepatide 5 mg	0	No concerns	Low risk	No concerns	No concerns	No concerns	No concerns	High	[]
Exenatide 2 mg:Icodec	0	No concerns	Low risk	No concerns	No concerns	Some concerns	No concerns	Moderate	["Heterogeneity"]
Exenatide 2 mg:Semaglutide 0.5 mg	0	No concerns	Low risk	No concerns	Major concerns	No concerns	No concerns	Low	["Imprecision"]
Exenatide 2 mg:Semaglutide 2 mg	0	No concerns	Low risk	No concerns	No concerns	Some concerns	No concerns	Moderate	["Heterogeneity"]

Exenatide 2 mg:Tirzepatide 10 mg	0	No concerns	Low risk	No concerns	No concerns	No concerns	No concerns	High	[]
Exenatide 2 mg:Tirzepatide 15 mg	0	No concerns	Low risk	No concerns	No concerns	No concerns	No concerns	High	[]
Exenatide 2 mg:Tirzepatide 5 mg	0	No concerns	Low risk	No concerns	No concerns	Some concerns	No concerns	Moderate	["Heterogeneity"]
Glargine:Semaglutide 2 mg	0	No concerns	Low risk	No concerns	Some concerns	Some concerns	No concerns	Moderate	["Imprecision","Heterogeneity"]
Icodec:Semaglutide 0.5 mg	0	No concerns	Low risk	No concerns	No concerns	Some concerns	No concerns	Moderate	["Heterogeneity"]
Icodec:Semaglutide 1 mg	0	No concerns	Low risk	No concerns	Major concerns	No concerns	No concerns	Low	["Imprecision"]
Icodec:Semaglutide 2 mg	0	No concerns	Low risk	No concerns	Major concerns	No concerns	No concerns	Low	["Imprecision"]
Icodec:Tirzepatide 10 mg	0	No concerns	Low risk	No concerns	No concerns	Major concerns	No concerns	Low	["Heterogeneity"]
Icodec:Tirzepatide 15 mg	0	No concerns	Low risk	No concerns	No concerns	Some concerns	No concerns	Moderate	["Heterogeneity"]
Icodec:Tirzepatide 5 mg	0	No concerns	Low risk	No concerns	Major concerns	No concerns	No concerns	Low	["Imprecision"]
Semaglutide 0.5 mg:Semaglutide 2 mg	0	No concerns	Low risk	No concerns	No concerns	No concerns	No concerns	High	[]
Semaglutide 0.5 mg:Tirzepatide 10 mg	0	No concerns	Low risk	No concerns	No concerns	No concerns	No concerns	High	[]
Semaglutide 0.5 mg:Tirzepatide 15 mg	0	No concerns	Low risk	No concerns	No concerns	No concerns	No concerns	High	[]
Semaglutide 0.5 mg:Tirzepatide 5 mg	0	No concerns	Low risk	No concerns	No concerns	No concerns	No concerns	High	[]
Semaglutide 2 mg:Tirzepatide 10 mg	0	No concerns	Low risk	No concerns	Major concerns	No concerns	No concerns	Low	["Imprecision"]
Semaglutide 2 mg:Tirzepatide 15 mg	0	No concerns	Low risk	No concerns	Major concerns	No concerns	No concerns	Low	["Imprecision"]

Semaglutide 2 mg:Tirzepatide 5 mg	0	No concerns	Low risk	No concerns	Major concerns	No concerns	No concerns	Low	["Imprecision"]

S6.3 Certainty in effect estimates for change in BW

Comparison	Number of studies	Within-study bias	Reporting bias	Indirectness	Imprecision	Heterogeneity	Incoherence	Confidence rating	Reason(s) for downgrading
BIF:Degludec	2	No concerns	Low risk	No concerns	Major concerns	No concerns	No concerns	Low	["Imprecision"]
Degludec:Icodec	2	No concerns	Low risk	No concerns	Major concerns	No concerns	Major concerns	Low	["Imprecision","Incoherence"]
Degludec:Tirzepatide 10 mg	1	No concerns	Low risk	No concerns	No concerns	No concerns	No concerns	High	[]
Degludec:Tirzepatide 15 mg	1	No concerns	Low risk	No concerns	No concerns	No concerns	No concerns	High	[]
Degludec:Tirzepatide 5 mg	1	No concerns	Low risk	No concerns	No concerns	No concerns	No concerns	High	[]
Dulaglutide 0.75 mg:Dulaglutide 1.5 mg	5	No concerns	Low risk	No concerns	Some concerns	Some concerns	No concerns	Moderate	["Imprecision","Heterogeneity"]
Dulaglutide 0.75 mg:Glargine	4	No concerns	Low risk	No concerns	No concerns	No concerns	No concerns	High	[]
Dulaglutide 0.75 mg:Semaglutide 0.5 mg	3	No concerns	Low risk	No concerns	No concerns	No concerns	No concerns	High	[]
Dulaglutide 0.75 mg:Semaglutide 1 mg	1	No concerns	Low risk	No concerns	No concerns	No concerns	No concerns	High	[]
Dulaglutide 0.75 mg:Tirzepatide 10 mg	1	No concerns	Low risk	No concerns	No concerns	No concerns	No concerns	High	[]
Dulaglutide 0.75 mg:Tirzepatide 15 mg	1	No concerns	Low risk	No concerns	No concerns	No concerns	No concerns	High	[]
Dulaglutide 0.75 mg:Tirzepatide 5 mg	1	No concerns	Low risk	No concerns	No concerns	No concerns	No concerns	High	[]
Dulaglutide 1.5 mg:Glargine	4	No concerns	Low risk	No concerns	No concerns	No concerns	No concerns	High	[]

Dulaglutide 1.5 mg:Semaglutide 0.5 mg	1	No concerns	Low risk	No concerns	No concerns	Major concerns	No concerns	Low	["Heterogeneity"]
Dulaglutide 1.5 mg:Semaglutide 1 mg	1	No concerns	Low risk	No concerns	No concerns	No concerns	No concerns	High	[]
Exenatide 2 mg:Glargine	2	No concerns	Low risk	No concerns	No concerns	No concerns	No concerns	High	[]
Exenatide 2 mg:Semaglutide 1 mg	1	No concerns	Low risk	No concerns	No concerns	No concerns	No concerns	High	[]
Glargine:Icodec	4	No concerns	Low risk	No concerns	No concerns	Major concerns	Major concerns	Low	["Heterogeneity","Incoherence"]
Glargine:Semaglutide 0.5 mg	1	No concerns	Low risk	No concerns	No concerns	No concerns	No concerns	High	[]
Glargine:Semaglutide 1 mg	1	No concerns	Low risk	No concerns	No concerns	No concerns	No concerns	High	[]
Glargine:Tirzepatide 10 mg	2	No concerns	Low risk	No concerns	No concerns	No concerns	No concerns	High	[]
Glargine:Tirzepatide 15 mg	2	No concerns	Low risk	No concerns	No concerns	No concerns	No concerns	High	[]
Glargine:Tirzepatide 5 mg	2	No concerns	Low risk	No concerns	No concerns	No concerns	No concerns	High	[]
Semaglutide 0.5 mg:Semaglutide 1 mg	2	No concerns	Low risk	No concerns	No concerns	Major concerns	No concerns	Low	["Heterogeneity"]
Semaglutide 1 mg:Semaglutide 2 mg	1	No concerns	Low risk	No concerns	Major concerns	No concerns	No concerns	Low	["Imprecision"]
Semaglutide 1 mg:Tirzepatide 10 mg	1	No concerns	Low risk	No concerns	No concerns	No concerns	No concerns	High	[]
Semaglutide 1 mg:Tirzepatide 15 mg	1	No concerns	Low risk	No concerns	No concerns	No concerns	No concerns	High	[]
Semaglutide 1 mg:Tirzepatide 5 mg	1	No concerns	Low risk	No concerns	No concerns	Major concerns	No concerns	Low	["Heterogeneity"]
Tirzepatide 10 mg:Tirzepatide 15 mg	5	No concerns	Low risk	No concerns	No concerns	Major concerns	No concerns	Low	["Heterogeneity"]

Tirzepatide 10 mg:Tirzepatide 5 mg	5	No concerns	Low risk	No concerns	No concerns	No concerns	No concerns	High	[]
Tirzepatide 15 mg:Tirzepatide 5 mg	5	No concerns	Low risk	No concerns	No concerns	No concerns	No concerns	High	[]
BIF:Dulaglutide 0.75 mg	0	No concerns	Low risk	No concerns	No concerns	No concerns	No concerns	High	[]
BIF:Dulaglutide 1.5 mg	0	No concerns	Low risk	No concerns	No concerns	No concerns	No concerns	High	[]
BIF:Exenatide 2 mg	0	No concerns	Low risk	No concerns	No concerns	No concerns	No concerns	High	[]
BIF:Glargine	0	No concerns	Low risk	No concerns	Some concerns	Some concerns	No concerns	Moderate	["Imprecision","Heterogeneity"]
BIF:Icodec	0	No concerns	Low risk	No concerns	Major concerns	No concerns	No concerns	Low	["Imprecision"]
BIF:Semaglutide 0.5 mg	0	No concerns	Low risk	No concerns	No concerns	No concerns	No concerns	High	[]
BIF:Semaglutide 1 mg	0	No concerns	Low risk	No concerns	No concerns	No concerns	No concerns	High	[]
BIF:Semaglutide 2 mg	0	No concerns	Low risk	No concerns	No concerns	No concerns	No concerns	High	[]
BIF:Tirzepatide 10 mg	0	No concerns	Low risk	No concerns	No concerns	No concerns	No concerns	High	[]
BIF:Tirzepatide 15 mg	0	No concerns	Low risk	No concerns	No concerns	No concerns	No concerns	High	[]
BIF:Tirzepatide 5 mg	0	No concerns	Low risk	No concerns	No concerns	No concerns	No concerns	High	[]
Degludec:Dulaglutide 0.75 mg	0	No concerns	Low risk	No concerns	No concerns	No concerns	No concerns	High	[]
Degludec:Dulaglutide 1.5 mg	0	No concerns	Low risk	No concerns	No concerns	No concerns	No concerns	High	[]
Degludec:Exenatide 2 mg	0	No concerns	Low risk	No concerns	No concerns	No concerns	No concerns	High	[]

Degludec:Glargine	0	No concerns	Low risk	No concerns	Major concerns	No concerns	No concerns	Low	["Imprecision"]
Degludec:Semaglutide 0.5 mg	0	No concerns	Low risk	No concerns	No concerns	No concerns	No concerns	High	[]
Degludec:Semaglutide 1 mg	0	No concerns	Low risk	No concerns	No concerns	No concerns	No concerns	High	[]
Degludec:Semaglutide 2 mg	0	No concerns	Low risk	No concerns	No concerns	No concerns	No concerns	High	[]
Dulaglutide 0.75 mg:Exenatide 2 mg	0	No concerns	Low risk	No concerns	Major concerns	No concerns	No concerns	Low	["Imprecision"]
Dulaglutide 0.75 mg:Icodec	0	No concerns	Low risk	No concerns	No concerns	No concerns	No concerns	High	[]
Dulaglutide 0.75 mg:Semaglutide 2 mg	0	No concerns	Low risk	No concerns	No concerns	No concerns	No concerns	High	[]
Dulaglutide 1.5 mg:Exenatide 2 mg	0	No concerns	Low risk	No concerns	Major concerns	No concerns	No concerns	Low	["Imprecision"]
Dulaglutide 1.5 mg:Icodec	0	No concerns	Low risk	No concerns	No concerns	No concerns	No concerns	High	[]
Dulaglutide 1.5 mg:Semaglutide 2 mg	0	No concerns	Low risk	No concerns	No concerns	No concerns	No concerns	High	[]
Dulaglutide 1.5 mg:Tirzepatide 10 mg	0	No concerns	Low risk	No concerns	No concerns	No concerns	No concerns	High	[]
Dulaglutide 1.5 mg:Tirzepatide 15 mg	0	No concerns	Low risk	No concerns	No concerns	No concerns	No concerns	High	[]
Dulaglutide 1.5 mg:Tirzepatide 5 mg	0	No concerns	Low risk	No concerns	No concerns	No concerns	No concerns	High	[]
Exenatide 2 mg:Icodec	0	No concerns	Low risk	No concerns	No concerns	No concerns	No concerns	High	[]
Exenatide 2 mg:Semaglutide 0.5 mg	0	No concerns	Low risk	No concerns	No concerns	Major concerns	No concerns	Low	["Heterogeneity"]
Exenatide 2 mg:Semaglutide 2 mg	0	No concerns	Low risk	No concerns	No concerns	No concerns	No concerns	High	[]

Exenatide 2 mg:Tirzepatide 10 mg	0	No concerns	Low risk	No concerns	No concerns	No concerns	No concerns	High	[]
Exenatide 2 mg:Tirzepatide 15 mg	0	No concerns	Low risk	No concerns	No concerns	No concerns	No concerns	High	[]
Exenatide 2 mg:Tirzepatide 5 mg	0	No concerns	Low risk	No concerns	No concerns	No concerns	No concerns	High	[]
Glargine:Semaglutide 2 mg	0	No concerns	Low risk	No concerns	No concerns	No concerns	No concerns	High	[]
Icodec:Semaglutide 0.5 mg	0	No concerns	Low risk	No concerns	No concerns	No concerns	No concerns	High	[]
Icodec:Semaglutide 1 mg	0	No concerns	Low risk	No concerns	No concerns	No concerns	No concerns	High	[]
Icodec:Semaglutide 2 mg	0	No concerns	Low risk	No concerns	No concerns	No concerns	No concerns	High	[]
Icodec:Tirzepatide 10 mg	0	No concerns	Low risk	No concerns	No concerns	No concerns	No concerns	High	[]
Icodec:Tirzepatide 15 mg	0	No concerns	Low risk	No concerns	No concerns	No concerns	No concerns	High	[]
Icodec:Tirzepatide 5 mg	0	No concerns	Low risk	No concerns	No concerns	No concerns	No concerns	High	[]
Semaglutide 0.5 mg:Semaglutide 2 mg	0	No concerns	Low risk	No concerns	No concerns	Major concerns	No concerns	Low	["Heterogeneity"]
Semaglutide 0.5 mg:Tirzepatide 10 mg	0	No concerns	Low risk	No concerns	No concerns	No concerns	No concerns	High	[]
Semaglutide 0.5 mg:Tirzepatide 15 mg	0	No concerns	Low risk	No concerns	No concerns	No concerns	No concerns	High	[]
Semaglutide 0.5 mg:Tirzepatide 5 mg	0	No concerns	Low risk	No concerns	No concerns	No concerns	No concerns	High	[]
Semaglutide 2 mg:Tirzepatide 10 mg	0	No concerns	Low risk	No concerns	No concerns	No concerns	No concerns	High	[]
Semaglutide 2 mg:Tirzepatide 15 mg	0	No concerns	Low risk	No concerns	No concerns	No concerns	No concerns	High	[]

Semaglutide 2 mg:Tirzepatide 5 mg	0	No concerns	Low risk	No concerns	Major concerns	No concerns	No concerns	Low	["Imprecision"]

S6.4 Certainty in effect estimates for incidence of TEAE

Comparison	Number of studies	Within-study bias	Reporting bias	Indirectness	Imprecision	Heterogeneity	Incoherence	Confidence rating	Reason(s) for downgrading
BIF:Degludec	2	No concerns	Low risk	No concerns	No concerns	No concerns	No concerns	High	[]
Degludec:Icodec	2	No concerns	Low risk	No concerns	No concerns	No concerns	No concerns	High	[]
Degludec:Tirzepatide 10 mg	1	No concerns	Low risk	No concerns	No concerns	No concerns	No concerns	High	[]
Degludec:Tirzepatide 15 mg	1	No concerns	Low risk	No concerns	No concerns	No concerns	No concerns	High	[]
Degludec:Tirzepatide 5 mg	1	No concerns	Low risk	No concerns	No concerns	No concerns	No concerns	High	[]
Dulaglutide 0.75 mg:Dulaglutide 1.5 mg	5	No concerns	Low risk	No concerns	No concerns	No concerns	No concerns	High	[]
Dulaglutide 0.75 mg:Glargine	4	No concerns	Low risk	No concerns	No concerns	No concerns	No concerns	High	[]
Dulaglutide 0.75 mg:Semaglutide 0.5 mg	1	No concerns	Low risk	No concerns	No concerns	No concerns	No concerns	High	[]
Dulaglutide 0.75 mg:Semaglutide 1 mg	1	No concerns	Low risk	No concerns	No concerns	No concerns	No concerns	High	[]
Dulaglutide 0.75 mg:Tirzepatide 10 mg	1	No concerns	Low risk	No concerns	No concerns	No concerns	No concerns	High	[]
Dulaglutide 0.75 mg:Tirzepatide 15 mg	1	No concerns	Low risk	No concerns	No concerns	No concerns	No concerns	High	[]
Dulaglutide 0.75 mg:Tirzepatide 5 mg	1	No concerns	Low risk	No concerns	No concerns	No concerns	No concerns	High	[]
Dulaglutide 1.5 mg:Glargine	4	No concerns	Low risk	No concerns	No concerns	No concerns	No concerns	High	[]
Dulaglutide 1.5 mg:Semaglutide 0.5 mg	1	No concerns	Low risk	No concerns	No concerns	No concerns	No concerns	High	[]

Dulaglutide 1.5 mg:Semaglutide 1 mg	1	No concerns	Low risk	No concerns	No concerns	No concerns	No concerns	High	[]
Exenatide 2 mg:Glargine	2	No concerns	Low risk	No concerns	No concerns	No concerns	No concerns	High	[]
Exenatide 2 mg:Semaglutide 1 mg	1	No concerns	Low risk	No concerns	No concerns	No concerns	No concerns	High	[]
Glargine:Icodec	4	No concerns	Low risk	No concerns	No concerns	No concerns	No concerns	High	[]
Glargine:Semaglutide 0.5 mg	1	No concerns	Low risk	No concerns	No concerns	No concerns	No concerns	High	[]
Glargine:Semaglutide 1 mg	1	No concerns	Low risk	No concerns	No concerns	No concerns	No concerns	High	[]
Glargine:Tirzepatide 10 mg	2	No concerns	Low risk	No concerns	No concerns	No concerns	No concerns	High	[]
Glargine:Tirzepatide 15 mg	2	No concerns	Low risk	No concerns	No concerns	No concerns	No concerns	High	[]
Glargine:Tirzepatide 5 mg	2	No concerns	Low risk	No concerns	No concerns	No concerns	No concerns	High	[]
Semaglutide 0.5 mg:Semaglutide 1 mg	2	No concerns	Low risk	No concerns	No concerns	No concerns	No concerns	High	[]
Semaglutide 1 mg:Semaglutide 2 mg	1	No concerns	Low risk	No concerns	No concerns	No concerns	No concerns	High	[]
Semaglutide 1 mg:Tirzepatide 10 mg	1	No concerns	Low risk	No concerns	No concerns	No concerns	No concerns	High	[]
Semaglutide 1 mg:Tirzepatide 15 mg	1	No concerns	Low risk	No concerns	No concerns	No concerns	No concerns	High	[]
Semaglutide 1 mg:Tirzepatide 5 mg	1	No concerns	Low risk	No concerns	No concerns	No concerns	No concerns	High	[]
Tirzepatide 10 mg:Tirzepatide 15 mg	5	No concerns	Low risk	No concerns	No concerns	No concerns	Major concerns	Low	["Incoherence"]
Tirzepatide 10 mg:Tirzepatide 5 mg	5	No concerns	Low risk	No concerns	No concerns	No concerns	Some concerns	Moderate	["Incoherence"]

Tirzepatide 15 mg:Tirzepatide 5 mg	5	No concerns	Low risk	No concerns	No concerns	No concerns	No concerns	High	[]
BIF:Dulaglutide 0.75 mg	0	No concerns	Low risk	No concerns	No concerns	No concerns	No concerns	High	[]
BIF:Dulaglutide 1.5 mg	0	No concerns	Low risk	No concerns	No concerns	No concerns	No concerns	High	[]
BIF:Exenatide 2 mg	0	No concerns	Low risk	No concerns	No concerns	No concerns	No concerns	High	[]
BIF:Glargine	0	No concerns	Low risk	No concerns	No concerns	No concerns	No concerns	High	[]
BIF:Icodec	0	No concerns	Low risk	No concerns	No concerns	No concerns	No concerns	High	[]
BIF:Semaglutide 0.5 mg	0	No concerns	Low risk	No concerns	No concerns	No concerns	No concerns	High	[]
BIF:Semaglutide 1 mg	0	No concerns	Low risk	No concerns	No concerns	No concerns	No concerns	High	[]
BIF:Semaglutide 2 mg	0	No concerns	Low risk	No concerns	No concerns	No concerns	No concerns	High	[]
BIF:Tirzepatide 10 mg	0	No concerns	Low risk	No concerns	No concerns	No concerns	No concerns	High	[]
BIF:Tirzepatide 15 mg	0	No concerns	Low risk	No concerns	No concerns	No concerns	No concerns	High	[]
BIF:Tirzepatide 5 mg	0	No concerns	Low risk	No concerns	No concerns	No concerns	No concerns	High	[]
Degludec:Dulaglutide 0.75 mg	0	No concerns	Low risk	No concerns	No concerns	No concerns	No concerns	High	[]
Degludec:Dulaglutide 1.5 mg	0	No concerns	Low risk	No concerns	No concerns	No concerns	No concerns	High	[]
Degludec:Exenatide 2 mg	0	No concerns	Low risk	No concerns	No concerns	No concerns	No concerns	High	[]
Degludec:Glargine	0	No concerns	Low risk	No concerns	No concerns	No concerns	No concerns	High	[]

Degludec:Semaglutide 0.5 mg	0	No concerns	Low risk	No concerns	No concerns	No concerns	No concerns	High	[]
Degludec:Semaglutide 1 mg	0	No concerns	Low risk	No concerns	No concerns	No concerns	No concerns	High	[]
Degludec:Semaglutide 2 mg	0	No concerns	Low risk	No concerns	No concerns	No concerns	No concerns	High	[]
Dulaglutide 0.75 mg:Exenatide 2 mg	0	No concerns	Low risk	No concerns	No concerns	No concerns	No concerns	High	[]
Dulaglutide 0.75 mg:Icodec	0	No concerns	Low risk	No concerns	No concerns	No concerns	No concerns	High	[]
Dulaglutide 0.75 mg:Semaglutide 2 mg	0	No concerns	Low risk	No concerns	No concerns	No concerns	No concerns	High	[]
Dulaglutide 1.5 mg:Exenatide 2 mg	0	No concerns	Low risk	No concerns	No concerns	No concerns	No concerns	High	[]
Dulaglutide 1.5 mg:Icodec	0	No concerns	Low risk	No concerns	No concerns	No concerns	No concerns	High	[]
Dulaglutide 1.5 mg:Semaglutide 2 mg	0	No concerns	Low risk	No concerns	No concerns	No concerns	No concerns	High	[]
Dulaglutide 1.5 mg:Tirzepatide 10 mg	0	No concerns	Low risk	No concerns	No concerns	No concerns	No concerns	High	[]
Dulaglutide 1.5 mg:Tirzepatide 15 mg	0	No concerns	Low risk	No concerns	No concerns	No concerns	No concerns	High	[]
Dulaglutide 1.5 mg:Tirzepatide 5 mg	0	No concerns	Low risk	No concerns	No concerns	No concerns	No concerns	High	[]
Exenatide 2 mg:Icodec	0	No concerns	Low risk	No concerns	No concerns	No concerns	No concerns	High	[]
Exenatide 2 mg:Semaglutide 0.5 mg	0	No concerns	Low risk	No concerns	No concerns	No concerns	No concerns	High	[]
Exenatide 2 mg:Semaglutide 2 mg	0	No concerns	Low risk	No concerns	No concerns	No concerns	No concerns	High	[]
Exenatide 2 mg:Tirzepatide 10 mg	0	No concerns	Low risk	No concerns	No concerns	No concerns	No concerns	High	[]

Exenatide 2 mg:Tirzepatide 15 mg	0	No concerns	Low risk	No concerns	No concerns	No concerns	No concerns	High	[]
Exenatide 2 mg:Tirzepatide 5 mg	0	No concerns	Low risk	No concerns	No concerns	No concerns	No concerns	High	[]
Glargine:Semaglutide 2 mg	0	No concerns	Low risk	No concerns	No concerns	No concerns	No concerns	High	[]
Icodec:Semaglutide 0.5 mg	0	No concerns	Low risk	No concerns	No concerns	No concerns	No concerns	High	[]
Icodec:Semaglutide 1 mg	0	No concerns	Low risk	No concerns	No concerns	No concerns	No concerns	High	[]
Icodec:Semaglutide 2 mg	0	No concerns	Low risk	No concerns	No concerns	No concerns	No concerns	High	[]
Icodec:Tirzepatide 10 mg	0	No concerns	Low risk	No concerns	No concerns	No concerns	No concerns	High	[]
Icodec:Tirzepatide 15 mg	0	No concerns	Low risk	No concerns	No concerns	No concerns	No concerns	High	[]
Icodec:Tirzepatide 5 mg	0	No concerns	Low risk	No concerns	No concerns	No concerns	No concerns	High	[]
Semaglutide 0.5 mg:Semaglutide 2 mg	0	No concerns	Low risk	No concerns	No concerns	No concerns	No concerns	High	[]
Semaglutide 0.5 mg:Tirzepatide 10 mg	0	No concerns	Low risk	No concerns	No concerns	No concerns	No concerns	High	[]
Semaglutide 0.5 mg:Tirzepatide 15 mg	0	No concerns	Low risk	No concerns	No concerns	No concerns	No concerns	High	[]
Semaglutide 0.5 mg:Tirzepatide 5 mg	0	No concerns	Low risk	No concerns	No concerns	No concerns	No concerns	High	[]
Semaglutide 2 mg:Tirzepatide 10 mg	0	No concerns	Low risk	No concerns	No concerns	No concerns	No concerns	High	[]
Semaglutide 2 mg:Tirzepatide 15 mg	0	No concerns	Low risk	No concerns	No concerns	No concerns	No concerns	High	[]
Semaglutide 2 mg:Tirzepatide 5 mg	0	No concerns	Low risk	No concerns	No concerns	No concerns	No concerns	High	[]

S6.5 Certainty in effect estimates for incidence of hypoglycemia

Comparison	Number of studies	Within-study bias	Reporting bias	Indirectness	Imprecision	Heterogeneity	Incoherence	Confidence rating	Reason(s) for downgrading
BIF:Degludec	2	Some concerns	Low risk	No concerns	No concerns	No concerns	Major concerns	Low	["Within-study bias","Incoherence"]
Degludec:Icodec	2	No concerns	Low risk	No concerns	No concerns	No concerns	Some concerns	Moderate	["Incoherence"]
Degludec:Tirzepatide 10 mg	1	No concerns	Low risk	No concerns	No concerns	No concerns	No concerns	High	[]
Degludec:Tirzepatide 15 mg	1	No concerns	Low risk	No concerns	No concerns	No concerns	Some concerns	Moderate	["Incoherence"]
Degludec:Tirzepatide 5 mg	1	No concerns	Low risk	No concerns	No concerns	No concerns	No concerns	High	[]
Dulaglutide 0.75 mg:Dulaglutide 1.5 mg	5	No concerns	Low risk	No concerns	No concerns	No concerns	Some concerns	Moderate	["Incoherence"]
Dulaglutide 0.75 mg:Glargine	4	No concerns	Low risk	No concerns	No concerns	No concerns	Some concerns	Moderate	["Incoherence"]
Dulaglutide 0.75 mg:Semaglutide 0.5 mg	3	No concerns	Low risk	No concerns	No concerns	No concerns	No concerns	High	[]
Dulaglutide 0.75 mg:Semaglutide 1 mg	1	No concerns	Low risk	No concerns	No concerns	No concerns	No concerns	High	[]
Dulaglutide 0.75 mg:Tirzepatide 10 mg	1	No concerns	Low risk	No concerns	No concerns	No concerns	No concerns	High	[]
Dulaglutide 0.75 mg:Tirzepatide 15 mg	1	No concerns	Low risk	No concerns	No concerns	No concerns	Some concerns	Moderate	["Incoherence"]
Dulaglutide 0.75 mg:Tirzepatide 5 mg	1	No concerns	Low risk	No concerns	No concerns	No concerns	No concerns	High	[]
Dulaglutide 1.5 mg:Glargine	4	No concerns	Low risk	No concerns	No concerns	No concerns	No concerns	High	[]

Dulaglutide 1.5 mg:Semaglutide 0.5 mg	1	No concerns	Low risk	No concerns	No concerns	No concerns	No concerns	High	[]
Dulaglutide 1.5 mg:Semaglutide 1 mg	1	No concerns	Low risk	No concerns	No concerns	No concerns	No concerns	High	[]
Exenatide 2 mg:Glargine	2	No concerns	Low risk	No concerns	No concerns	No concerns	No concerns	High	[]
Exenatide 2 mg:Semaglutide 1 mg	1	No concerns	Low risk	No concerns	No concerns	No concerns	No concerns	High	[]
Glargine:Icodec	4	No concerns	Low risk	No concerns	No concerns	No concerns	Some concerns	Moderate	["Incoherence"]
Glargine:Semaglutide 0.5 mg	1	No concerns	Low risk	No concerns	No concerns	Some concerns	No concerns	Moderate	["Heterogeneity"]
Glargine:Semaglutide 1 mg	1	No concerns	Low risk	No concerns	No concerns	Some concerns	No concerns	Moderate	["Heterogeneity"]
Glargine:Tirzepatide 10 mg	2	No concerns	Low risk	No concerns	No concerns	Some concerns	No concerns	Moderate	["Heterogeneity"]
Glargine:Tirzepatide 15 mg	2	No concerns	Low risk	No concerns	No concerns	Some concerns	No concerns	Moderate	["Heterogeneity"]
Glargine:Tirzepatide 5 mg	2	No concerns	Low risk	No concerns	No concerns	No concerns	Some concerns	Moderate	["Incoherence"]
Semaglutide 0.5 mg:Semaglutide 1 mg	2	No concerns	Low risk	No concerns	No concerns	No concerns	No concerns	High	[]
Semaglutide 1 mg:Semaglutide 2 mg	1	No concerns	Low risk	No concerns	No concerns	No concerns	Major concerns	Low	["Incoherence"]
Semaglutide 1 mg:Tirzepatide 10 mg	1	No concerns	Low risk	No concerns	No concerns	No concerns	No concerns	High	[]
Semaglutide 1 mg:Tirzepatide 15 mg	1	No concerns	Low risk	No concerns	No concerns	No concerns	Some concerns	Moderate	["Incoherence"]
Semaglutide 1 mg:Tirzepatide 5 mg	1	No concerns	Low risk	No concerns	No concerns	No concerns	No concerns	High	[]
Tirzepatide 10 mg:Tirzepatide 15 mg	5	No concerns	Low risk	No concerns	No concerns	No concerns	Some concerns	Moderate	["Incoherence"]

Tirzepatide 10 mg:Tirzepatide 5 mg	5	No concerns	Low risk	No concerns	No concerns	No concerns	No concerns	High	[]
Tirzepatide 15 mg:Tirzepatide 5 mg	5	No concerns	Low risk	No concerns	No concerns	No concerns	Some concerns	Moderate	["Incoherence"]
BIF:Dulaglutide 0.75 mg	0	No concerns	Low risk	No concerns	No concerns	No concerns	Major concerns	Low	["Incoherence"]
BIF:Dulaglutide 1.5 mg	0	No concerns	Low risk	No concerns	No concerns	Some concerns	Major concerns	Low	["Heterogeneity","Incoherence"]
BIF:Exenatide 2 mg	0	No concerns	Low risk	No concerns	No concerns	No concerns	Major concerns	Low	["Incoherence"]
BIF:Glargine	0	No concerns	Low risk	No concerns	No concerns	Some concerns	Major concerns	Low	["Heterogeneity","Incoherence"]
BIF:Icodec	0	No concerns	Low risk	No concerns	No concerns	No concerns	Major concerns	Low	["Incoherence"]
BIF:Semaglutide 0.5 mg	0	No concerns	Low risk	No concerns	No concerns	No concerns	Major concerns	Low	["Incoherence"]
BIF:Semaglutide 1 mg	0	No concerns	Low risk	No concerns	No concerns	No concerns	Major concerns	Low	["Incoherence"]
BIF:Semaglutide 2 mg	0	No concerns	Low risk	No concerns	No concerns	No concerns	Major concerns	Low	["Incoherence"]
BIF:Tirzepatide 10 mg	0	No concerns	Low risk	No concerns	No concerns	No concerns	Major concerns	Low	["Incoherence"]
BIF:Tirzepatide 15 mg	0	No concerns	Low risk	No concerns	No concerns	No concerns	Major concerns	Low	["Incoherence"]
BIF:Tirzepatide 5 mg	0	No concerns	Low risk	No concerns	No concerns	No concerns	Major concerns	Low	["Incoherence"]
Degludec:Dulaglutide 0.75 mg	0	No concerns	Low risk	No concerns	No concerns	Some concerns	Major concerns	Low	["Heterogeneity","Incoherence"]
Degludec:Dulaglutide 1.5 mg	0	No concerns	Low risk	No concerns	No concerns	Some concerns	Major concerns	Low	["Heterogeneity","Incoherence"]
Degludec:Exenatide 2 mg	0	No concerns	Low risk	No concerns	No concerns	No concerns	Major concerns	Low	["Incoherence"]

Degludec:Glargine	0	No concerns	Low risk	No concerns	No concerns	No concerns	Major concerns	Low	["Incoherence"]
Degludec:Semaglutide 0.5 mg	0	No concerns	Low risk	No concerns	No concerns	No concerns	Major concerns	Low	["Incoherence"]
Degludec:Semaglutide 1 mg	0	No concerns	Low risk	No concerns	No concerns	No concerns	Major concerns	Low	["Incoherence"]
Degludec:Semaglutide 2 mg	0	No concerns	Low risk	No concerns	No concerns	No concerns	Major concerns	Low	["Incoherence"]
Dulaglutide 0.75 mg:Exenatide 2 mg	0	No concerns	Low risk	No concerns	No concerns	No concerns	Major concerns	Low	["Incoherence"]
Dulaglutide 0.75 mg:Icodec	0	No concerns	Low risk	No concerns	No concerns	No concerns	Major concerns	Low	["Incoherence"]
Dulaglutide 0.75 mg:Semaglutide 2 mg	0	No concerns	Low risk	No concerns	No concerns	Some concerns	Major concerns	Low	["Heterogeneity","Incoherence"]
Dulaglutide 1.5 mg:Exenatide 2 mg	0	No concerns	Low risk	No concerns	No concerns	No concerns	Major concerns	Low	["Incoherence"]
Dulaglutide 1.5 mg:Icodec	0	No concerns	Low risk	No concerns	No concerns	Some concerns	Major concerns	Low	["Heterogeneity","Incoherence"]
Dulaglutide 1.5 mg:Semaglutide 2 mg	0	No concerns	Low risk	No concerns	Some concerns	No concerns	Major concerns	Low	["Imprecision","Incoherence"]
Dulaglutide 1.5 mg:Tirzepatide 10 mg	0	No concerns	Low risk	No concerns	No concerns	No concerns	Major concerns	Low	["Incoherence"]
Dulaglutide 1.5 mg:Tirzepatide 15 mg	0	No concerns	Low risk	No concerns	No concerns	No concerns	Major concerns	Low	["Incoherence"]
Dulaglutide 1.5 mg:Tirzepatide 5 mg	0	No concerns	Low risk	No concerns	No concerns	Some concerns	Major concerns	Low	["Heterogeneity","Incoherence"]
Exenatide 2 mg:Icodec	0	No concerns	Low risk	No concerns	No concerns	No concerns	Major concerns	Low	["Incoherence"]
Exenatide 2 mg:Semaglutide 0.5 mg	0	No concerns	Low risk	No concerns	No concerns	No concerns	Major concerns	Low	["Incoherence"]
Exenatide 2 mg:Semaglutide 2 mg	0	No concerns	Low risk	No concerns	No concerns	No concerns	Major concerns	Low	["Incoherence"]

Exenatide 2 mg:Tirzepatide 10 mg	0	No concerns	Low risk	No concerns	No concerns	No concerns	Major concerns	Low	["Incoherence"]
Exenatide 2 mg:Tirzepatide 15 mg	0	No concerns	Low risk	No concerns	No concerns	No concerns	Major concerns	Low	["Incoherence"]
Exenatide 2 mg:Tirzepatide 5 mg	0	No concerns	Low risk	No concerns	No concerns	No concerns	Major concerns	Low	["Incoherence"]
Glargine:Semaglutide 2 mg	0	No concerns	Low risk	No concerns	No concerns	Some concerns	Major concerns	Low	["Heterogeneity","Incoherence"]
Icodec:Semaglutide 0.5 mg	0	No concerns	Low risk	No concerns	No concerns	No concerns	Major concerns	Low	["Incoherence"]
Icodec:Semaglutide 1 mg	0	No concerns	Low risk	No concerns	No concerns	No concerns	Major concerns	Low	["Incoherence"]
Icodec:Semaglutide 2 mg	0	No concerns	Low risk	No concerns	No concerns	No concerns	Major concerns	Low	["Incoherence"]
Icodec:Tirzepatide 10 mg	0	No concerns	Low risk	No concerns	No concerns	No concerns	Major concerns	Low	["Incoherence"]
Icodec:Tirzepatide 15 mg	0	No concerns	Low risk	No concerns	No concerns	No concerns	Major concerns	Low	["Incoherence"]
Icodec:Tirzepatide 5 mg	0	No concerns	Low risk	No concerns	No concerns	No concerns	Major concerns	Low	["Incoherence"]
Semaglutide 0.5 mg:Semaglutide 2 mg	0	No concerns	Low risk	No concerns	No concerns	No concerns	Major concerns	Low	["Incoherence"]
Semaglutide 0.5 mg:Tirzepatide 10 mg	0	No concerns	Low risk	No concerns	No concerns	No concerns	Major concerns	Low	["Incoherence"]
Semaglutide 0.5 mg:Tirzepatide 15 mg	0	No concerns	Low risk	No concerns	No concerns	No concerns	Major concerns	Low	["Incoherence"]
Semaglutide 0.5 mg:Tirzepatide 5 mg	0	No concerns	Low risk	No concerns	No concerns	No concerns	Major concerns	Low	["Incoherence"]
Semaglutide 2 mg:Tirzepatide 10 mg	0	No concerns	Low risk	No concerns	No concerns	No concerns	Major concerns	Low	["Incoherence"]
Semaglutide 2 mg:Tirzepatide 15 mg	0	No concerns	Low risk	No concerns	No concerns	Some concerns	Major concerns	Low	["Heterogeneity","Incoherence"]

Semaglutide 2 mg:Tirzepatide 5 mg	0	No concerns	Low risk	No concerns	No concerns	No concerns	Major concerns	Low	["Incoherence"]

S6.6 Certainty in effect estimates for incidence of treatment discontinuation due to adverse events

Comparison	Number of studies	Within-study bias	Reporting bias	Indirectness	Imprecision	Heterogeneity	Incoherence	Confidence rating	Reason(s) for downgrading
BIF:Degludec	2	Some concerns	Low risk	No concerns	Some concerns	No concerns	No concerns	Moderate	["Within-study bias","Imprecision"]
Degludec:Icodec	2	No concerns	Low risk	No concerns	Some concerns	No concerns	No concerns	Moderate	["Imprecision"]
Degludec:Tirzepatide 10 mg	1	No concerns	Low risk	No concerns	No concerns	No concerns	No concerns	High	[]
Degludec:Tirzepatide 15 mg	1	No concerns	Low risk	No concerns	No concerns	No concerns	No concerns	High	[]
Degludec:Tirzepatide 5 mg	1	No concerns	Low risk	No concerns	No concerns	No concerns	No concerns	High	[]
Dulaglutide 0.75 mg:Dulaglutide 1.5 mg	5	No concerns	Low risk	No concerns	No concerns	No concerns	No concerns	High	[]
Dulaglutide 0.75 mg:Glargine	4	No concerns	Low risk	No concerns	No concerns	No concerns	No concerns	High	[]
Dulaglutide 0.75 mg:Semaglutide 0.5 mg	1	No concerns	Low risk	No concerns	No concerns	No concerns	No concerns	High	[]
Dulaglutide 0.75 mg:Semaglutide 1 mg	1	No concerns	Low risk	No concerns	No concerns	No concerns	No concerns	High	[]
Dulaglutide 0.75 mg:Tirzepatide 10 mg	1	No concerns	Low risk	No concerns	No concerns	Some concerns	No concerns	Moderate	["Heterogeneity"]
Dulaglutide 0.75 mg:Tirzepatide 15 mg	1	No concerns	Low risk	No concerns	No concerns	Some concerns	No concerns	Moderate	["Heterogeneity"]
Dulaglutide 0.75 mg:Tirzepatide 5 mg	1	No concerns	Low risk	No concerns	No concerns	No concerns	No concerns	High	[]
Dulaglutide 1.5 mg:Glargine	4	No concerns	Low risk	No concerns	No concerns	No concerns	No concerns	High	[]

Dulaglutide 1.5 mg:Semaglutide 0.5 mg	1	No concerns	Low risk	No concerns	No concerns	No concerns	No concerns	High	[]
Dulaglutide 1.5 mg:Semaglutide 1 mg	1	No concerns	Low risk	No concerns	No concerns	No concerns	No concerns	High	[]
Exenatide 2 mg:Glargine	2	No concerns	Low risk	No concerns	No concerns	No concerns	No concerns	High	[]
Exenatide 2 mg:Semaglutide 1 mg	1	No concerns	Low risk	No concerns	No concerns	No concerns	No concerns	High	[]
Glargine:Icodec	4	No concerns	Low risk	No concerns	No concerns	No concerns	No concerns	High	[]
Glargine:Semaglutide 0.5 mg	1	No concerns	Low risk	No concerns	No concerns	Some concerns	No concerns	Moderate	["Heterogeneity"]
Glargine:Semaglutide 1 mg	1	No concerns	Low risk	No concerns	No concerns	Some concerns	No concerns	Moderate	["Heterogeneity"]
Glargine:Tirzepatide 10 mg	2	No concerns	Low risk	No concerns	No concerns	No concerns	No concerns	High	[]
Glargine:Tirzepatide 15 mg	2	No concerns	Low risk	No concerns	No concerns	No concerns	No concerns	High	[]
Glargine:Tirzepatide 5 mg	2	No concerns	Low risk	No concerns	No concerns	Some concerns	No concerns	Moderate	["Heterogeneity"]
Semaglutide 0.5 mg:Semaglutide 1 mg	2	No concerns	Low risk	No concerns	No concerns	No concerns	No concerns	High	[]
Semaglutide 1 mg:Semaglutide 2 mg	1	No concerns	Low risk	No concerns	No concerns	No concerns	No concerns	High	[]
Semaglutide 1 mg:Tirzepatide 10 mg	1	No concerns	Low risk	No concerns	No concerns	No concerns	Some concerns	Moderate	["Incoherence"]
Semaglutide 1 mg:Tirzepatide 15 mg	1	No concerns	Low risk	No concerns	No concerns	No concerns	Some concerns	Moderate	["Incoherence"]
Semaglutide 1 mg:Tirzepatide 5 mg	1	No concerns	Low risk	No concerns	No concerns	No concerns	No concerns	High	[]
Tirzepatide 10 mg:Tirzepatide 15 mg	5	No concerns	Low risk	No concerns	No concerns	No concerns	No concerns	High	[]

Tirzepatide 10 mg:Tirzepatide 5 mg	5	No concerns	Low risk	No concerns	No concerns	No concerns	No concerns	High	[]
Tirzepatide 15 mg:Tirzepatide 5 mg	5	No concerns	Low risk	No concerns	No concerns	No concerns	No concerns	High	[]
BIF:Dulaglutide 0.75 mg	0	No concerns	Low risk	No concerns	Some concerns	No concerns	No concerns	Moderate	["Imprecision"]
BIF:Dulaglutide 1.5 mg	0	No concerns	Low risk	No concerns	Some concerns	No concerns	No concerns	Moderate	["Imprecision"]
BIF:Exenatide 2 mg	0	No concerns	Low risk	No concerns	Some concerns	No concerns	No concerns	Moderate	["Imprecision"]
BIF:Glargine	0	No concerns	Low risk	No concerns	Some concerns	Some concerns	No concerns	Moderate	["Imprecision","Heterogeneity"]
BIF:Icodec	0	No concerns	Low risk	No concerns	Some concerns	No concerns	No concerns	Moderate	["Imprecision"]
BIF:Semaglutide 0.5 mg	0	No concerns	Low risk	No concerns	Some concerns	No concerns	No concerns	Moderate	["Imprecision"]
BIF:Semaglutide 1 mg	0	No concerns	Low risk	No concerns	Some concerns	No concerns	No concerns	Moderate	["Imprecision"]
BIF:Semaglutide 2 mg	0	No concerns	Low risk	No concerns	Some concerns	No concerns	No concerns	Moderate	["Imprecision"]
BIF:Tirzepatide 10 mg	0	No concerns	Low risk	No concerns	Some concerns	No concerns	No concerns	Moderate	["Imprecision"]
BIF:Tirzepatide 15 mg	0	No concerns	Low risk	No concerns	Some concerns	No concerns	No concerns	Moderate	["Imprecision"]
BIF:Tirzepatide 5 mg	0	No concerns	Low risk	No concerns	Some concerns	No concerns	No concerns	Moderate	["Imprecision"]
Degludec:Dulaglutide 0.75 mg	0	No concerns	Low risk	No concerns	No concerns	Some concerns	No concerns	Moderate	["Heterogeneity"]
Degludec:Dulaglutide 1.5 mg	0	No concerns	Low risk	No concerns	No concerns	Some concerns	No concerns	Moderate	["Heterogeneity"]
Degludec:Exenatide 2 mg	0	No concerns	Low risk	No concerns	No concerns	No concerns	No concerns	High	[]

Degludec:Glargine	0	No concerns	Low risk	No concerns	Some concerns	No concerns	No concerns	Moderate	["Imprecision"]
Degludec:Semaglutide 0.5 mg	0	No concerns	Low risk	No concerns	No concerns	No concerns	No concerns	High	[]
Degludec:Semaglutide 1 mg	0	No concerns	Low risk	No concerns	No concerns	No concerns	No concerns	High	[]
Degludec:Semaglutide 2 mg	0	No concerns	Low risk	No concerns	No concerns	No concerns	No concerns	High	[]
Dulaglutide 0.75 mg:Exenatide 2 mg	0	No concerns	Low risk	No concerns	No concerns	Some concerns	No concerns	Moderate	["Heterogeneity"]
Dulaglutide 0.75 mg:Icodec	0	No concerns	Low risk	No concerns	No concerns	No concerns	No concerns	High	[]
Dulaglutide 0.75 mg:Semaglutide 2 mg	0	No concerns	Low risk	No concerns	Some concerns	No concerns	No concerns	Moderate	["Imprecision"]
Dulaglutide 1.5 mg:Exenatide 2 mg	0	No concerns	Low risk	No concerns	No concerns	Some concerns	No concerns	Moderate	["Heterogeneity"]
Dulaglutide 1.5 mg:Icodec	0	No concerns	Low risk	No concerns	No concerns	No concerns	No concerns	High	[]
Dulaglutide 1.5 mg:Semaglutide 2 mg	0	No concerns	Low risk	No concerns	Some concerns	No concerns	No concerns	Moderate	["Imprecision"]
Dulaglutide 1.5 mg:Tirzepatide 10 mg	0	No concerns	Low risk	No concerns	No concerns	Some concerns	No concerns	Moderate	["Heterogeneity"]
Dulaglutide 1.5 mg:Tirzepatide 15 mg	0	No concerns	Low risk	No concerns	No concerns	Some concerns	No concerns	Moderate	["Heterogeneity"]
Dulaglutide 1.5 mg:Tirzepatide 5 mg	0	No concerns	Low risk	No concerns	No concerns	No concerns	No concerns	High	[]
Exenatide 2 mg:Icodec	0	No concerns	Low risk	No concerns	No concerns	Some concerns	No concerns	Moderate	["Heterogeneity"]
Exenatide 2 mg:Semaglutide 0.5 mg	0	No concerns	Low risk	No concerns	No concerns	No concerns	No concerns	High	[]
Exenatide 2 mg:Semaglutide 2 mg	0	No concerns	Low risk	No concerns	No concerns	No concerns	No concerns	High	[]

Exenatide 2 mg:Tirzepatide 10 mg	0	No concerns	Low risk	No concerns	No concerns	No concerns	No concerns	High	[]
Exenatide 2 mg:Tirzepatide 15 mg	0	No concerns	Low risk	No concerns	No concerns	No concerns	No concerns	High	[]
Exenatide 2 mg:Tirzepatide 5 mg	0	No concerns	Low risk	No concerns	No concerns	No concerns	No concerns	High	[]
Glargine:Semaglutide 2 mg	0	No concerns	Low risk	No concerns	No concerns	Some concerns	No concerns	Moderate	["Heterogeneity"]
Icodec:Semaglutide 0.5 mg	0	No concerns	Low risk	No concerns	Some concerns	No concerns	No concerns	Moderate	["Imprecision"]
Icodec:Semaglutide 1 mg	0	No concerns	Low risk	No concerns	No concerns	Some concerns	No concerns	Moderate	["Heterogeneity"]
Icodec:Semaglutide 2 mg	0	No concerns	Low risk	No concerns	Some concerns	No concerns	No concerns	Moderate	["Imprecision"]
Icodec:Tirzepatide 10 mg	0	No concerns	Low risk	No concerns	No concerns	No concerns	No concerns	High	[]
Icodec:Tirzepatide 15 mg	0	No concerns	Low risk	No concerns	No concerns	No concerns	No concerns	High	[]
Icodec:Tirzepatide 5 mg	0	No concerns	Low risk	No concerns	Some concerns	No concerns	No concerns	Moderate	["Imprecision"]
Semaglutide 0.5 mg:Semaglutide 2 mg	0	No concerns	Low risk	No concerns	No concerns	No concerns	No concerns	High	[]
Semaglutide 0.5 mg:Tirzepatide 10 mg	0	No concerns	Low risk	No concerns	No concerns	No concerns	No concerns	High	[]
Semaglutide 0.5 mg:Tirzepatide 15 mg	0	No concerns	Low risk	No concerns	No concerns	No concerns	No concerns	High	[]
Semaglutide 0.5 mg:Tirzepatide 5 mg	0	No concerns	Low risk	No concerns	No concerns	No concerns	No concerns	High	[]
Semaglutide 2 mg:Tirzepatide 10 mg	0	No concerns	Low risk	No concerns	No concerns	No concerns	No concerns	High	[]
Semaglutide 2 mg:Tirzepatide 15 mg	0	No concerns	Low risk	No concerns	No concerns	No concerns	No concerns	High	[]

Semaglutide 2 mg:Tirzepatide 5 mg	0	No concerns	Low risk	No concerns	No concerns	No concerns	No concerns	High	[]

S6.7 Certainty in effect estimates for the incidence of serious adverse events

Comparison	Number of studies	Within-study bias	Reporting bias	Indirectness	Imprecision	Heterogeneity	Incoherence	Confidence rating	Reason(s) for downgrading
BIF:Daily insulin	2	No concerns	Low risk	No concerns	No concerns	No concerns	Some concerns	Moderate	["Incoherence"]
Daily insulin:Dulaglutide 0.75 mg	4	No concerns	Low risk	No concerns	No concerns	No concerns	No concerns	High	[]
Daily insulin:Dulaglutide 1.50 mg	4	No concerns	Low risk	No concerns	No concerns	No concerns	No concerns	High	[]
Daily insulin:Exenatide 2 mg	2	No concerns	Low risk	No concerns	No concerns	No concerns	Major concerns	Low	["Incoherence"]
Daily insulin:Icodec	6	No concerns	Low risk	No concerns	No concerns	No concerns	Some concerns	Moderate	["Incoherence"]
Daily insulin:Semaglutide 0.5 mg	1	No concerns	Low risk	No concerns	No concerns	No concerns	No concerns	High	[]
Daily insulin:Semaglutide 1 mg	1	No concerns	Low risk	No concerns	No concerns	No concerns	No concerns	High	[]
Daily insulin:Tirzepatide 10 mg	3	No concerns	Low risk	No concerns	No concerns	No concerns	No concerns	High	[]
Daily insulin:Tirzepatide 15 mg	3	No concerns	Low risk	No concerns	No concerns	No concerns	No concerns	High	[]
Daily insulin:Tirzepatide 5 mg	3	No concerns	Low risk	No concerns	No concerns	No concerns	No concerns	High	[]

Dulaglutide 0.75 mg:Dulaglutide 1.5 mg	1	No concerns	Low risk	No concerns	No concerns	No concerns	No concerns	High	[]
Dulaglutide 0.75 mg:Dulaglutide 1.50 mg	4	No concerns	Low risk	No concerns	No concerns	No concerns	No concerns	High	[]
Dulaglutide 0.75 mg:Semaglutide 0.5 mg	1	No concerns	Low risk	No concerns	No concerns	No concerns	No concerns	High	[]
Dulaglutide 0.75 mg:Semaglutide 1 mg	1	No concerns	Low risk	No concerns	No concerns	No concerns	No concerns	High	[]
Dulaglutide 1.5 mg:Semaglutide 0.5 mg	1	No concerns	Low risk	No concerns	No concerns	No concerns	No concerns	High	[]
Dulaglutide 1.5 mg:Semaglutide 1 mg	1	No concerns	Low risk	No concerns	No concerns	No concerns	No concerns	High	[]
Dulagutide 0.75 mg:Tirzepatide 10 mg	1	No concerns	Low risk	No concerns	No concerns	Some concerns	No concerns	Moderate	["Heterogeneity"]
Dulagutide 0.75 mg:Tirzepatide 15 mg	1	No concerns	Low risk	No concerns	Some concerns	No concerns	No concerns	Moderate	["Imprecision"]
Dulagutide 0.75 mg:Tirzepatide 5 mg	1	No concerns	Low risk	No concerns	No concerns	Some concerns	No concerns	Moderate	["Heterogeneity"]
Exenatide 2 mg:Semaglutide 1 mg	1	No concerns	Low risk	No concerns	No concerns	No concerns	Some concerns	Moderate	["Incoherence"]
Semaglutide 0.5 mg:Semaglutide 1 mg	2	No concerns	Low risk	No concerns	No concerns	No concerns	No concerns	High	[]
Semaglutide 1 mg:Semaglutide 2 mg	1	No concerns	Low risk	No concerns	No concerns	Some concerns	Some concerns	Moderate	["Heterogeneity","Incoherence"]
Semaglutide 1 mg:Tirzepatide 10 mg	1	No concerns	Low risk	No concerns	No concerns	No concerns	No concerns	High	[]
Semaglutide 1 mg:Tirzepatide 15 mg	1	No concerns	Low risk	No concerns	No concerns	No concerns	Some concerns	Moderate	["Incoherence"]
Semaglutide 1 mg:Tirzepatide 5 mg	1	No concerns	Low risk	No concerns	No concerns	No concerns	Some concerns	Moderate	["Incoherence"]
Tirzepatide 10 mg:Tirzepatide 15 mg	5	No concerns	Low risk	No concerns	No concerns	No concerns	No concerns	High	[]

Tirzepatide 10 mg:Tirzepatide 5 mg	5	No concerns	Low risk	No concerns	No concerns	No concerns	No concerns	High	[]
Tirzepatide 15 mg:Tirzepatide 5 mg	5	No concerns	Low risk	No concerns	No concerns	No concerns	Some concerns	Moderate	["Incoherence"]
BIF:Dulaglutide 0.75 mg	0	No concerns	Low risk	No concerns	No concerns	No concerns	Some concerns	Moderate	["Incoherence"]
BIF:Dulaglutide 1.5 mg	0	No concerns	Low risk	No concerns	No concerns	Some concerns	Some concerns	Moderate	["Heterogeneity","Incoherence"]
BIF:Dulaglutide 1.50 mg	0	No concerns	Low risk	No concerns	No concerns	No concerns	Some concerns	Moderate	["Incoherence"]
BIF:Dulagutide 0.75 mg	0	No concerns	Low risk	No concerns	Some concerns	No concerns	Some concerns	Moderate	["Imprecision","Incoherence"]
BIF:Exenatide 2 mg	0	No concerns	Low risk	No concerns	No concerns	Some concerns	Some concerns	Moderate	["Heterogeneity","Incoherence"]
BIF:Icodec	0	No concerns	Low risk	No concerns	No concerns	No concerns	Some concerns	Moderate	["Incoherence"]
BIF:Semaglutide 0.5 mg	0	No concerns	Low risk	No concerns	No concerns	No concerns	Some concerns	Moderate	["Incoherence"]
BIF:Semaglutide 1 mg	0	No concerns	Low risk	No concerns	No concerns	No concerns	Some concerns	Moderate	["Incoherence"]
BIF:Semaglutide 2 mg	0	No concerns	Low risk	No concerns	Some concerns	No concerns	Some concerns	Moderate	["Imprecision","Incoherence"]
BIF:Tirzepatide 10 mg	0	No concerns	Low risk	No concerns	No concerns	No concerns	Some concerns	Moderate	["Incoherence"]
BIF:Tirzepatide 15 mg	0	No concerns	Low risk	No concerns	No concerns	No concerns	Some concerns	Moderate	["Incoherence"]
BIF:Tirzepatide 5 mg	0	No concerns	Low risk	No concerns	No concerns	No concerns	Some concerns	Moderate	["Incoherence"]
Daily insulin:Dulaglutide 1.5 mg	0	No concerns	Low risk	No concerns	No concerns	No concerns	Some concerns	Moderate	["Incoherence"]
Daily insulin:Dulagutide 0.75 mg	0	No concerns	Low risk	No concerns	Some concerns	No concerns	Some concerns	Moderate	["Imprecision","Incoherence"]

Daily insulin:Semaglutide 2 mg	0	No concerns	Low risk	No concerns	No concerns	Some concerns	Some concerns	Moderate	["Heterogeneity","Incoherence"]
Dulaglutide 0.75 mg:Dulagutide 0.75 mg	0	No concerns	Low risk	No concerns	Some concerns	No concerns	Some concerns	Moderate	["Imprecision","Incoherence"]
Dulaglutide 0.75 mg:Exenatide 2 mg	0	No concerns	Low risk	No concerns	No concerns	No concerns	Some concerns	Moderate	["Incoherence"]
Dulaglutide 0.75 mg:Icodec	0	No concerns	Low risk	No concerns	No concerns	No concerns	Some concerns	Moderate	["Incoherence"]
Dulaglutide 0.75 mg:Semaglutide 2 mg	0	No concerns	Low risk	No concerns	No concerns	Some concerns	Some concerns	Moderate	["Heterogeneity","Incoherence"]
Dulaglutide 0.75 mg:Tirzepatide 10 mg	0	No concerns	Low risk	No concerns	No concerns	No concerns	Some concerns	Moderate	["Incoherence"]
Dulaglutide 0.75 mg:Tirzepatide 15 mg	0	No concerns	Low risk	No concerns	No concerns	No concerns	Some concerns	Moderate	["Incoherence"]
Dulaglutide 0.75 mg:Tirzepatide 5 mg	0	No concerns	Low risk	No concerns	No concerns	No concerns	Some concerns	Moderate	["Incoherence"]
Dulaglutide 1.50 mg:Dulaglutide 1.5 mg	0	No concerns	Low risk	No concerns	No concerns	No concerns	Some concerns	Moderate	["Incoherence"]
Dulaglutide 1.5 mg:Dulagutide 0.75 mg	0	No concerns	Low risk	No concerns	Some concerns	No concerns	Some concerns	Moderate	["Imprecision","Incoherence"]
Dulaglutide 1.5 mg:Exenatide 2 mg	0	No concerns	Low risk	No concerns	No concerns	Some concerns	Some concerns	Moderate	["Heterogeneity","Incoherence"]
Dulaglutide 1.5 mg:Icodec	0	No concerns	Low risk	No concerns	No concerns	No concerns	Some concerns	Moderate	["Incoherence"]
Dulaglutide 1.5 mg:Semaglutide 2 mg	0	No concerns	Low risk	No concerns	Some concerns	No concerns	Some concerns	Moderate	["Imprecision","Incoherence"]
Dulaglutide 1.5 mg:Tirzepatide 10 mg	0	No concerns	Low risk	No concerns	No concerns	No concerns	Some concerns	Moderate	["Incoherence"]
Dulaglutide 1.5 mg:Tirzepatide 15 mg	0	No concerns	Low risk	No concerns	No concerns	No concerns	Some concerns	Moderate	["Incoherence"]
Dulaglutide 1.5 mg:Tirzepatide 5 mg	0	No concerns	Low risk	No concerns	No concerns	No concerns	Some concerns	Moderate	["Incoherence"]

Dulaglutide 1.50 mg:Dulagutide 0.75 mg	0	No concerns	Low risk	No concerns	Some concerns	No concerns	Some concerns	Moderate	["Imprecision","Incoherence"]
Dulaglutide 1.50 mg:Exenatide 2 mg	0	No concerns	Low risk	No concerns	No concerns	No concerns	Some concerns	Moderate	["Incoherence"]
Dulaglutide 1.50 mg:Icodec	0	No concerns	Low risk	No concerns	No concerns	No concerns	Some concerns	Moderate	["Incoherence"]
Dulaglutide 1.50 mg:Semaglutide 0.5 mg	0	No concerns	Low risk	No concerns	No concerns	No concerns	Some concerns	Moderate	["Incoherence"]
Dulaglutide 1.50 mg:Semaglutide 1 mg	0	No concerns	Low risk	No concerns	No concerns	No concerns	Some concerns	Moderate	["Incoherence"]
Dulaglutide 1.50 mg:Semaglutide 2 mg	0	No concerns	Low risk	No concerns	Some concerns	No concerns	Some concerns	Moderate	["Imprecision","Incoherence"]
Dulaglutide 1.50 mg:Tirzepatide 10 mg	0	No concerns	Low risk	No concerns	No concerns	No concerns	Some concerns	Moderate	["Incoherence"]
Dulaglutide 1.50 mg:Tirzepatide 15 mg	0	No concerns	Low risk	No concerns	No concerns	No concerns	Some concerns	Moderate	["Incoherence"]
Dulaglutide 1.50 mg:Tirzepatide 5 mg	0	No concerns	Low risk	No concerns	No concerns	No concerns	Some concerns	Moderate	["Incoherence"]
Dulagutide 0.75 mg:Exenatide 2 mg	0	No concerns	Low risk	No concerns	No concerns	Some concerns	Some concerns	Moderate	["Heterogeneity","Incoherence"]
Dulagutide 0.75 mg:Icodec	0	No concerns	Low risk	No concerns	Some concerns	No concerns	Some concerns	Moderate	["Imprecision","Incoherence"]
Dulagutide 0.75 mg:Semaglutide 0.5 mg	0	No concerns	Low risk	No concerns	Some concerns	No concerns	Some concerns	Moderate	["Imprecision","Incoherence"]
Dulagutide 0.75 mg:Semaglutide 1 mg	0	No concerns	Low risk	No concerns	Some concerns	No concerns	Some concerns	Moderate	["Imprecision","Incoherence"]
Dulagutide 0.75 mg:Semaglutide 2 mg	0	No concerns	Low risk	No concerns	Some concerns	No concerns	Some concerns	Moderate	["Imprecision","Incoherence"]
Exenatide 2 mg:Icodec	0	No concerns	Low risk	No concerns	No concerns	No concerns	Some concerns	Moderate	["Incoherence"]
Exenatide 2 mg:Semaglutide 0.5 mg	0	No concerns	Low risk	No concerns	No concerns	Some concerns	Some concerns	Moderate	["Heterogeneity","Incoherence"]

Exenatide 2 mg:Semaglutide 2 mg	0	No concerns	Low risk	No concerns	Some concerns	No concerns	Some concerns	Moderate	["Imprecision","Incoherence"]
Exenatide 2 mg:Tirzepatide 10 mg	0	No concerns	Low risk	No concerns	No concerns	No concerns	Some concerns	Moderate	["Incoherence"]
Exenatide 2 mg:Tirzepatide 15 mg	0	No concerns	Low risk	No concerns	No concerns	Some concerns	Some concerns	Moderate	["Heterogeneity","Incoherence"]
Exenatide 2 mg:Tirzepatide 5 mg	0	No concerns	Low risk	No concerns	No concerns	No concerns	Some concerns	Moderate	["Incoherence"]
Icodec:Semaglutide 0.5 mg	0	No concerns	Low risk	No concerns	No concerns	No concerns	Some concerns	Moderate	["Incoherence"]
Icodec:Semaglutide 1 mg	0	No concerns	Low risk	No concerns	No concerns	No concerns	Some concerns	Moderate	["Incoherence"]
Icodec:Semaglutide 2 mg	0	No concerns	Low risk	No concerns	Some concerns	No concerns	Some concerns	Moderate	["Imprecision","Incoherence"]
Icodec:Tirzepatide 10 mg	0	No concerns	Low risk	No concerns	No concerns	No concerns	Some concerns	Moderate	["Incoherence"]
Icodec:Tirzepatide 15 mg	0	No concerns	Low risk	No concerns	No concerns	No concerns	Some concerns	Moderate	["Incoherence"]
Icodec:Tirzepatide 5 mg	0	No concerns	Low risk	No concerns	No concerns	No concerns	Some concerns	Moderate	["Incoherence"]
Semaglutide 0.5 mg:Semaglutide 2 mg	0	No concerns	Low risk	No concerns	No concerns	Some concerns	Some concerns	Moderate	["Heterogeneity","Incoherence"]
Semaglutide 0.5 mg:Tirzepatide 10 mg	0	No concerns	Low risk	No concerns	No concerns	No concerns	Some concerns	Moderate	["Incoherence"]
Semaglutide 0.5 mg:Tirzepatide 15 mg	0	No concerns	Low risk	No concerns	No concerns	No concerns	Some concerns	Moderate	["Incoherence"]
Semaglutide 0.5 mg:Tirzepatide 5 mg	0	No concerns	Low risk	No concerns	No concerns	No concerns	Some concerns	Moderate	["Incoherence"]
Semaglutide 2 mg:Tirzepatide 10 mg	0	No concerns	Low risk	No concerns	No concerns	Some concerns	Some concerns	Moderate	["Heterogeneity","Incoherence"]
Semaglutide 2 mg:Tirzepatide 15 mg	0	No concerns	Low risk	No concerns	No concerns	Some concerns	Some concerns	Moderate	["Heterogeneity","Incoherence"]

Semaglutide 2 mg:Tirzepatide 5 mg	0	No concerns	Low risk	No concerns	Some concerns	No concerns	Some concerns	Moderate	["Imprecision","Incoherence"]

Supplement S7. Meta-regression results:

S7.1 Meta-regression results for HbA1c (age, diabetes duration)

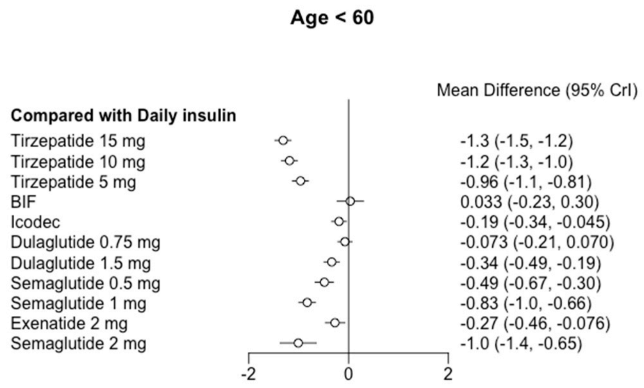

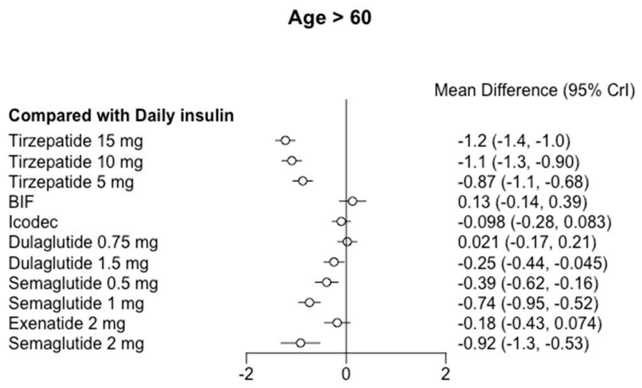

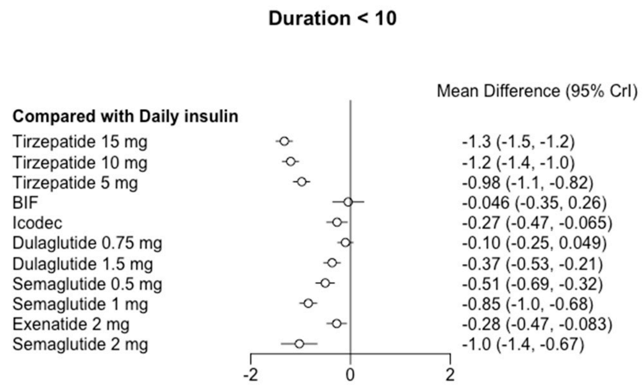

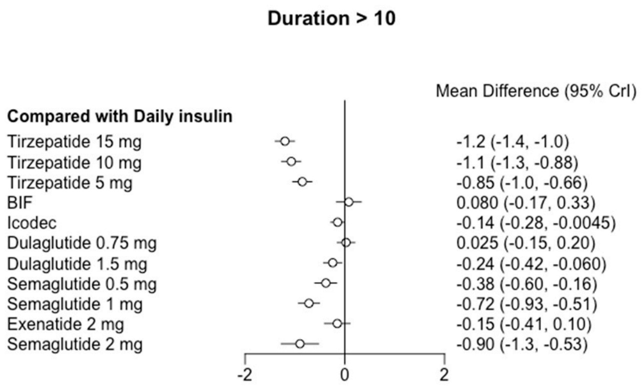

S7.2 Meta-regression results for FPG (age, diabetes duration)

Age > 60

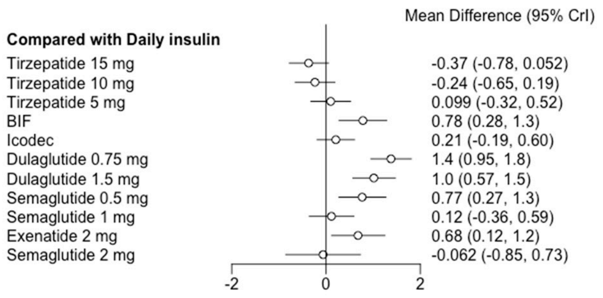

Age < 60

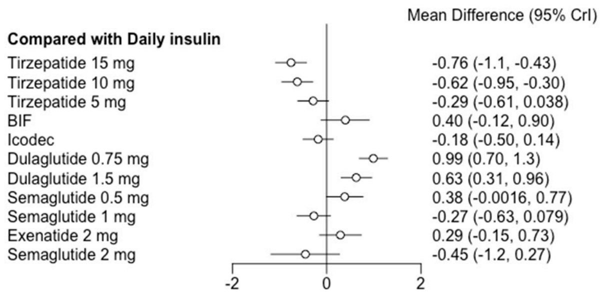

Duration > 10

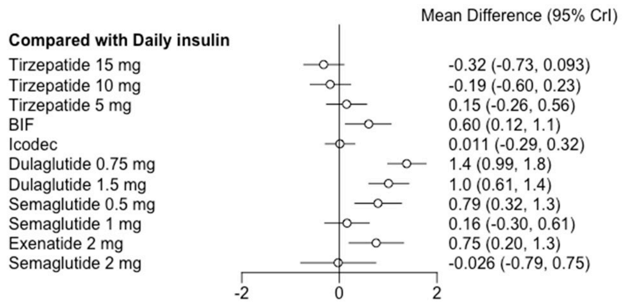

Duration < 10

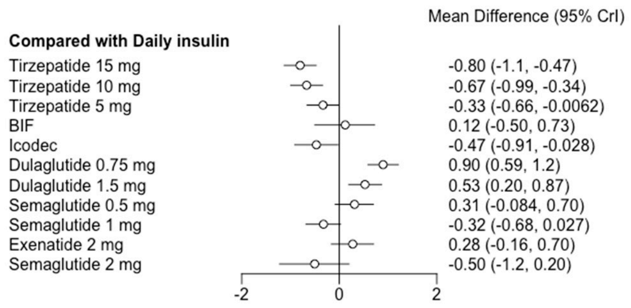

S7.3 Meta-regression results for BW (age, diabetes duration)

Age < 60

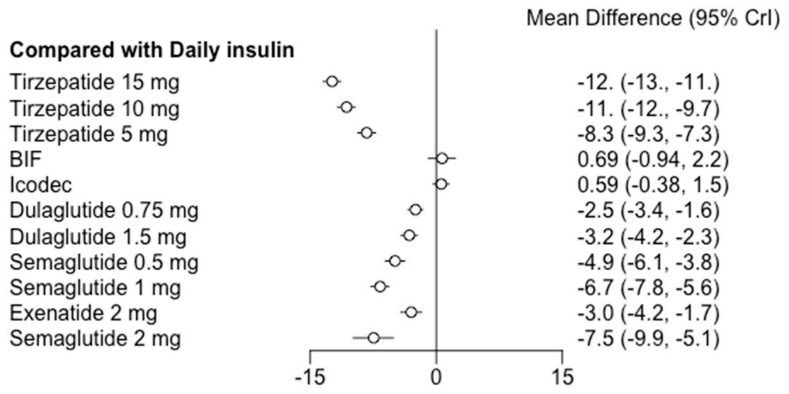

Age > 60

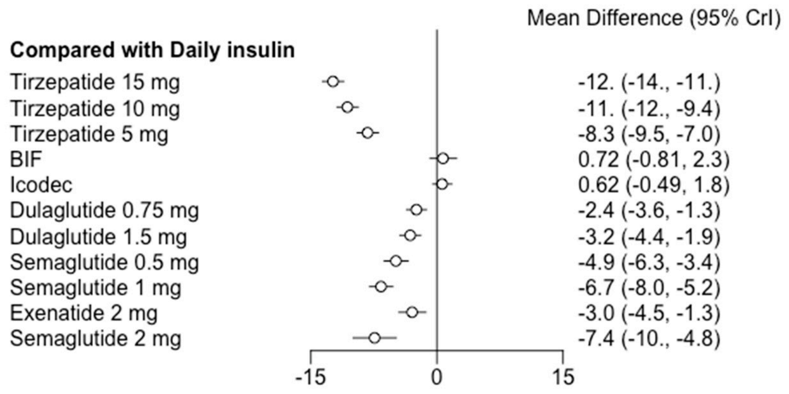

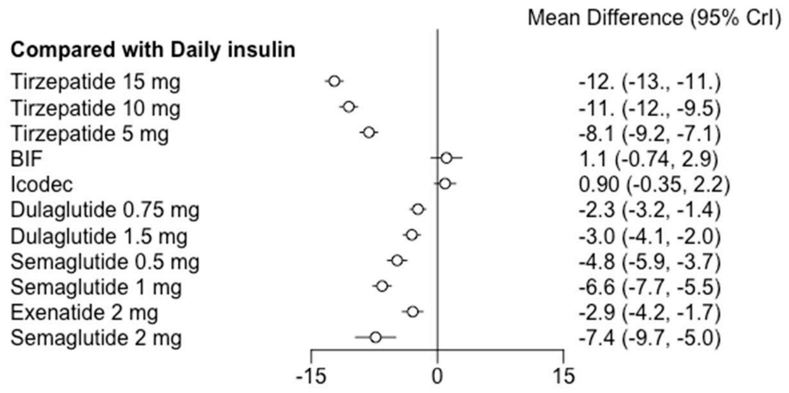

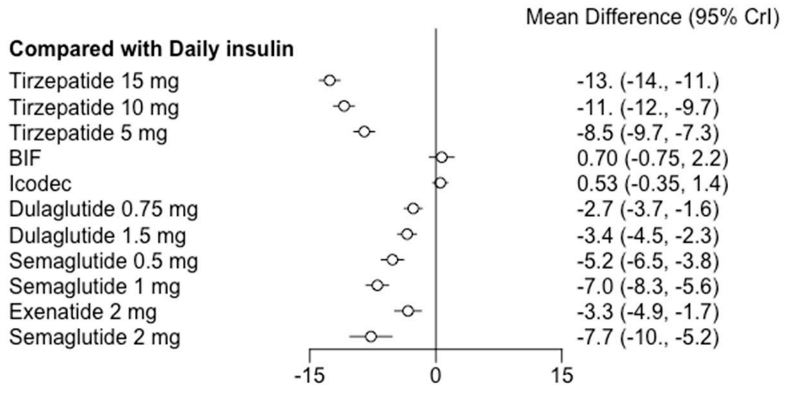

Supplement S8. Summary of the excluded studies:

	Study ID	Reason for exclusion
1.	Umpierrez, 2014[26]	Not the intervention of interest
2.	Wysham, 2014[27]	Not the intervention of interest
3.	Weinstock, 2015[28]	Not the intervention of interest
4.	Dungan, 2014[29]	Not the study design of interest
5.	Dungan, 2016[30]	Not the study design of interest
6.	Pozzilli, 2017[31]	Not the study design of interest
7.	Chen, 2018[32]	Not the intervention of interest
8.	Battelino,2022[33]	Not the study design of interest
9.	Gastaldelli,2022[34]	Not the study design of interest
10.	Wilson, 2020[35]	Not the study design of interest

Supplement S9. Evaluation of heterogeneity and inconsistency under full design-by-treatment interaction random effects model

Outcome	I^2	Q	p-value
Change from baseline in HbA1c	80.6%	8.33	0.6832
Change from baseline in BW	90.3%	10.81	0.4595
Change from baseline in FPG	75.6%	5.19	0.9215
Incidence of hypoglycemia	68.8%	19.50	0.0530
Incidence of Adverse events	63.4%	4.97	0.893
Incidence of treatment emergent adverse events	35.5%	2.506	0.991
Incidence of serious adverse events	64.4%	13.91	0.0841

Supplement S10. PRISMA checklist

Section and Topic	Item #	Checklist item	Location where item is reported
**TITLE**			
Title	1	Identify the report as a systematic review.	1
**ABSTRACT**			
Abstract	2	See the PRISMA 2020 for Abstracts checklist.	1
**INTRODUCTION**			
Rationale	3	Describe the rationale for the review in the context of existing knowledge.	1
Objectives	4	Provide an explicit statement of the objective(s) or question(s) the review addresses.	1
**METHODS**			
Eligibility criteria	5	Specify the inclusion and exclusion criteria for the review and how studies were grouped for the syntheses.	2,3
Information sources	6	Specify all databases, registers, websites, organisations, reference lists and other sources searched or consulted to identify studies. Specify the date when each source was last searched or consulted.	2,3
Search strategy	7	Present the full search strategies for all databases, registers and websites, including any filters and limits used.	2,3
Selection process	8	Specify the methods used to decide whether a study met the inclusion criteria of the review, including how many reviewers screened each record and each report retrieved, whether they worked independently, and if applicable, details of automation tools used in the process.	2,3 protocol
Data collection process	9	Specify the methods used to collect data from reports, including how many reviewers collected data from each report, whether they worked independently, any processes for obtaining or confirming data from study investigators, and if applicable, details of automation tools used in the process.	2,3 protocol
Data items	10a	List and define all outcomes for which data were sought. Specify whether all results that were compatible with each outcome domain in each study were sought (e.g. for all measures, time points, analyses), and if not, the methods used to decide which results to collect.	2,3 protocol
	10b	List and define all other variables for which data were sought (e.g. participant and intervention characteristics, funding sources). Describe any assumptions made about any missing or unclear information.	2,3 protocol
Study risk of bias assessment	11	Specify the methods used to assess risk of bias in the included studies, including details of the tool(s) used, how many reviewers assessed each study and whether they worked independently, and if applicable, details of automation tools used in the process.	2,3
Effect measures	12	Specify for each outcome the effect measure(s) (e.g. risk ratio, mean difference) used in the synthesis or presentation of results.	2,3
Synthesis methods	13a	Describe the processes used to decide which studies were eligible for each synthesis (e.g. tabulating the study intervention characteristics and comparing against the planned groups for each synthesis (item #5)).	2,3
	13b	Describe any methods required to prepare the data for presentation or synthesis, such as handling of missing summary statistics, or data conversions.	2,3
	13c	Describe any methods used to tabulate or visually display results of individual studies and syntheses.	2,3
	13d	Describe any methods used to synthesize results and provide a rationale for the choice(s). If meta-analysis was performed, describe the model(s), method(s) to identify the presence and extent of statistical heterogeneity, and software package(s) used.	2,3
	13e	Describe any methods used to explore possible causes of heterogeneity among study results (e.g. subgroup analysis, meta-regression).	2,3
	13f	Describe any sensitivity analyses conducted to assess robustness of the synthesized results.	2,3

Supplement S10. PRISMA checklist			
**Section and Topic**	**Item #**	**Checklist item**	**Location where item is reported**
Reporting bias assessment	14	Describe any methods used to assess risk of bias due to missing results in a synthesis (arising from reporting biases).	2,3
Certainty assessment	15	Describe any methods used to assess certainty (or confidence) in the body of evidence for an outcome.	2,3
**RESULTS**			
Study selection	16a	Describe the results of the search and selection process, from the number of records identified in the search to the number of studies included in the review, ideally using a flow diagram.	3-6
	16b	Cite studies that might appear to meet the inclusion criteria, but which were excluded, and explain why they were excluded.	3-6
Study characteristics	17	Cite each included study and present its characteristics.	3-6
Risk of bias in studies	18	Present assessments of risk of bias for each included study.	3-6
Results of individual studies	19	For all outcomes, present, for each study: (a) summary statistics for each group (where appropriate) and (b) an effect estimate and its precision (e.g. confidence/credible interval), ideally using structured tables or plots.	3-6
Results of syntheses	20a	For each synthesis, briefly summarise the characteristics and risk of bias among contributing studies.	3-6
	20b	Present results of all statistical syntheses conducted. If meta-analysis was done, present for each the summary estimate and its precision (e.g. confidence/credible interval) and measures of statistical heterogeneity. If comparing groups, describe the direction of the effect.	3-6
	20c	Present results of all investigations of possible causes of heterogeneity among study results.	3-6
	20d	Present results of all sensitivity analyses conducted to assess the robustness of the synthesized results.	3-6
Reporting biases	21	Present assessments of risk of bias due to missing results (arising from reporting biases) for each synthesis assessed.	3-6
Certainty of evidence	22	Present assessments of certainty (or confidence) in the body of evidence for each outcome assessed.	3-6
**DISCUSSION**			
Discussion	23a	Provide a general interpretation of the results in the context of other evidence.	6-7
	23b	Discuss any limitations of the evidence included in the review.	6-7
	23c	Discuss any limitations of the review processes used.	6-7
	23d	Discuss implications of the results for practice, policy, and future research.	6-7
**OTHER INFORMATION**			
Registration and protocol	24a	Provide registration information for the review, including register name and registration number, or state that the review was not registered.	2
	24b	Indicate where the review protocol can be accessed, or state that a protocol was not prepared.	2

Supplement S10. PRISMA checklist

Section and Topic	Item #	Checklist item	Location where item is reported
	24c	Describe and explain any amendments to information provided at registration or in the protocol.	2
Support	25	Describe sources of financial or non-financial support for the review, and the role of the funders or sponsors in the review.	7,8
Competing interests	26	Declare any competing interests of review authors.	7,8
Availability of data, code and other materials	27	Report which of the following are publicly available and where they can be found: template data collection forms; data extracted from included studies; data used for all analyses; analytic code; any other materials used in the review.	7,8

References:

1. Ahmann AJ, Capehorn M, Charpentier G, et al. Efficacy and Safety of Once-Weekly Semaglutide Versus Exenatide ER in Subjects With Type 2 Diabetes (SUSTAIN 3): A 56-Week, Open-Label, Randomized Clinical Trial. *Diabetes Care*. Feb 2018;41(2):258-266. doi:10.2337/dc17-0417
2. Aroda VR, Bain SC, Cariou B, et al. Efficacy and safety of once-weekly semaglutide versus once-daily insulin glargine as add-on to metformin (with or without sulfonylureas) in insulin-naive patients with type 2 diabetes (SUSTAIN 4): a randomised, open-label, parallel-group, multicentre, multinational, phase 3a trial. Article. *The Lancet Diabetes and Endocrinology*. 2017;5(5):355-366. doi:10.1016/S2213-8587(17)30085-2
3. Bajaj HS, Bergenstal RM, Christoffersen A, et al. Switching to Once-Weekly Insulin Icodec Versus Once-Daily Insulin Glargine U100 in Type 2 Diabetes Inadequately Controlled on Daily Basal Insulin: A Phase 2 Randomized Controlled Trial. *Diabetes care*. 2021 07 (Epub 2021 Apr 2021;44(7):1586-1594. doi:10.2337/dc20-2877
4. Blonde L, Jendle J, Gross J, et al. Once-weekly dulaglutide versus bedtime insulin glargine, both in combination with prandial insulin lispro, in patients with type 2 diabetes (AWARD-4): a randomised, open-label, phase 3, non-inferiority study. *Lancet (London, England)*. 2015 May 2015;385(9982):2057-66. doi:10.1016/s0140-6736(15)60936-9
5. Bue-Valleskey JM, Kazda CM, Ma C, et al. Once-Weekly Basal Insulin Fc Demonstrated Similar Glycemic Control to Once-Daily Insulin Degludec in Insulin-Naive Patients With Type 2 Diabetes: A Phase 2 Randomized Control Trial. *Diabetes care*. 2023 05 2023;46(5):1060-1067. doi:10.2337/dc22-2396
6. Diamant M, Van Gaal L, Stranks S, et al. Once weekly exenatide compared with insulin glargine titrated to target in patients with type 2 diabetes (DURATION-3): an open-label randomised trial. *Lancet (London, England)*. 2010 Jun 2010;375(9733):2234-43. doi:10.1016/s0140-6736(10)60406-0
7. Frías JP, Auerbach P, Bajaj HS, et al. Efficacy and safety of once-weekly semaglutide 2·0 mg versus 1·0 mg in patients with type 2 diabetes (SUSTAIN FORTE): a double-blind, randomised, phase 3B trial. *Lancet Diabetes Endocrinol*. Sep 2021;9(9):563-574. doi:10.1016/s2213-8587(21)00174-1
8. Frías JP, Davies MJ, Rosenstock J, et al. Tirzepatide versus Semaglutide Once Weekly in Patients with Type 2 Diabetes. *N Engl J Med*. Aug 5 2021;385(6):503-515. doi:10.1056/NEJMoa2107519
9. Frias J, Chien J, Zhang Q, et al. Safety and efficacy of once-weekly basal insulin Fc in people with type 2 diabetes previously treated with basal insulin: a multicentre, open-label, randomised, phase 2 study. *The lancet Diabetes & endocrinology*. 2023 03 (Epub 2023 Feb 2023;11(3):158-168. doi:10.1016/s2213-8587(22)00388-6
10. Gao L, Lee BW, Chawla M, et al. Tirzepatide versus insulin glargine as second-line or third-line therapy in type 2 diabetes in the Asia-Pacific region: the SURPASS-AP-Combo trial. *Nat Med*. Jun 2023;29(6):1500-1510. doi:10.1038/s41591-023-02344-1
11. Giorgino F, Benroubi M, Sun J-H, Zimmermann AG, Pechtner V. Efficacy and Safety of Once-Weekly Dulaglutide Versus Insulin Glargine in Patients With Type 2 Diabetes on Metformin

and Glimepiride (AWARD-2). *Diabetes care*. 2015 Dec (Epub 2015 Jun 2015;38(12):2241-9. doi:10.2337/dc14-1625
12. Inagaki N, Atsumi Y, Oura T, Saito H, Imaoka T. Efficacy and Safety Profile of Exenatide Once Weekly Compared With Insulin Once Daily in Japanese Patients With Type 2 Diabetes Treated With Oral Antidiabetes Drug(s): Results From a 26-Week, Randomized, Open-Label, Parallel-Group, Multicenter, Noninferiority Study. Article. *Clinical Therapeutics*. 2012;34(9):1892-1908.e1. doi:10.1016/j.clinthera.2012.07.007
13. Inagaki N, Takeuchi M, Oura T, Imaoka T, Seino Y. Efficacy and safety of tirzepatide monotherapy compared with dulaglutide in Japanese patients with type 2 diabetes (SURPASS J-mono): a double-blind, multicentre, randomised, phase 3 trial. *Lancet Diabetes Endocrinol*. Sep 2022;10(9):623-633. doi:10.1016/s2213-8587(22)00188-7
14. Kimura T, Katakura Y, Shimoda M, et al. Comparison of clinical efficacy and safety of weekly glucagon-like peptide-1 receptor agonists dulaglutide and semaglutide in Japanese patients with type 2 diabetes: Randomized, parallel-group, multicentre, open-label trial (COMING study). *Diabetes, Obesity and Metabolism*. 2023;25(12):3632-3647. doi:https://doi.org/10.1111/dom.15258
15. Lingvay I, Asong M, Desouza C, et al. Once-Weekly Insulin Icodec vs Once-Daily Insulin Degludec in Adults With Insulin-Naive Type 2 Diabetes: The ONWARDS 3 Randomized Clinical Trial. *Jama*. 2023 07 2023;330(3):228-237. doi:10.1001/jama.2023.11313
16. Ludvik B, Giorgino F, Jódar E, et al. Once-weekly tirzepatide versus once-daily insulin degludec as add-on to metformin with or without SGLT2 inhibitors in patients with type 2 diabetes (SURPASS-3): a randomised, open-label, parallel-group, phase 3 trial. *Lancet*. Aug 14 2021;398(10300):583-598. doi:10.1016/s0140-6736(21)01443-4
17. Mathieu C, Ásbjörnsdóttir B, Bajaj HS, et al. Switching to once-weekly insulin icodec versus once-daily insulin glargine U100 in individuals with basal-bolus insulin-treated type 2 diabetes (ONWARDS 4): a phase 3a, randomised, open-label, multicentre, treat-to-target, non-inferiority trial. *Lancet*. Jun 10 2023;401(10392):1929-1940. doi:10.1016/s0140-6736(23)00520-2
18. Philis-Tsimikas A, Asong M, Franek E, et al. Switching to once-weekly insulin icodec versus once-daily insulin degludec in individuals with basal insulin-treated type 2 diabetes (ONWARDS 2): a phase 3a, randomised, open label, multicentre, treat-to-target trial. *Lancet Diabetes Endocrinol*. Jun 2023;11(6):414-425. doi:10.1016/s2213-8587(23)00093-1
19. Pratley RE, Aroda VR, Lingvay I, et al. Semaglutide versus dulaglutide once weekly in patients with type 2 diabetes (SUSTAIN 7): a randomised, open-label, phase 3b trial. *Lancet Diabetes Endocrinol*. Apr 2018;6(4):275-286. doi:10.1016/s2213-8587(18)30024-x
20. Del Prato S, Kahn SE, Pavo I, et al. Tirzepatide versus insulin glargine in type 2 diabetes and increased cardiovascular risk (SURPASS-4): a randomised, open-label, parallel-group, multicentre, phase 3 trial. *Lancet*. Nov 13 2021;398(10313):1811-1824. doi:10.1016/s0140-6736(21)02188-7
21. Rosenstock J, Bajaj HS, Janez A, et al. Once-Weekly Insulin for Type 2 Diabetes without Previous Insulin Treatment. *The New England journal of medicine*. 2020 11 26 (Epub 2020 Sep 2020;383(22):2107-2116. doi:10.1056/NEJMoa2022474

22. Rosenstock J, Bain SC, Gowda A, et al. Weekly Icodec versus Daily Glargine U100 in Type 2 Diabetes without Previous Insulin. *N Engl J Med*. Jul 27 2023;389(4):297-308. doi:10.1056/NEJMoa2303208
23. Takahashi Y, Nomoto H, Yokoyama H, et al. Improvement of glycaemic control and treatment satisfaction by switching from liraglutide or dulaglutide to subcutaneous semaglutide in patients with type 2 diabetes: A multicentre, prospective, randomized, open-label, parallel-group comparison study (SWITCH-SEMA 1 study). *Diabetes Obes Metab*. Jun 2023;25(6):1503-1511. doi:10.1111/dom.14998
24. Tuttle KR, Lakshmanan MC, Rayner B, et al. Dulaglutide versus insulin glargine in patients with type 2 diabetes and moderate-to-severe chronic kidney disease (AWARD-7): a multicentre, open-label, randomised trial. *The lancet Diabetes & endocrinology*. 2018 08 (Epub 2018 Jun 2018;6(8):605-617. doi:10.1016/s2213-8587(18)30104-9
25. Wang J, Li H-Q, Xu X-H, et al. The Effects of Once-Weekly Dulaglutide and Insulin Glargine on Glucose Fluctuation in Poorly Oral-Antidiabetic Controlled Patients with Type 2 Diabetes Mellitus. *BioMed research international*. 2019 2019;2019:2682657. doi:10.1155/2019/2682657
26. Umpierrez G, Tofé Povedano S, Pérez Manghi F, Shurzinske L, Pechtner V. Efficacy and safety of dulaglutide monotherapy versus metformin in type 2 diabetes in a randomized controlled trial (AWARD-3). *Diabetes Care*. Aug 2014;37(8):2168-76. doi:10.2337/dc13-2759
27. Wysham C, Blevins T, Arakaki R, et al. Efficacy and safety of dulaglutide added onto pioglitazone and metformin versus exenatide in type 2 diabetes in a randomized controlled trial (AWARD-1). *Diabetes Care*. Aug 2014;37(8):2159-67. doi:10.2337/dc13-2760
28. Weinstock RS, Guerci B, Umpierrez G, Nauck MA, Skrivanek Z, Milicevic Z. Safety and efficacy of once-weekly dulaglutide versus sitagliptin after 2 years in metformin-treated patients with type 2 diabetes (AWARD-5): a randomized, phase III study. *Diabetes Obes Metab*. Sep 2015;17(9):849-58. doi:10.1111/dom.12479
29. Dungan KM, Povedano ST, Forst T, et al. Once-weekly dulaglutide versus once-daily liraglutide in metformin-treated patients with type 2 diabetes (AWARD-6): a randomised, open-label, phase 3, non-inferiority trial. *Lancet (London, England)*. 2014 Oct 11 (Epub 2014 Jul 2014;384(9951):1349-57. doi:10.1016/s0140-6736(14)60976-4
30. Dungan KM, Weitgasser R, Perez Manghi F, et al. A 24-week study to evaluate the efficacy and safety of once-weekly dulaglutide added on to glimepiride in type 2 diabetes (AWARD-8). *Diabetes Obes Metab*. May 2016;18(5):475-82. doi:10.1111/dom.12634
31. Pozzilli P, Norwood P, Jodar E, et al. Placebo-controlled, randomized trial of the addition of once-weekly glucagon-like peptide-1 receptor agonist dulaglutide to titrated daily insulin glargine in patients with type 2 diabetes (AWARD-9). *Diabetes, obesity & metabolism*. 2017 07 (Epub 2017 Apr 2017;19(7):1024-1031. doi:10.1111/dom.12937
32. Chen YH, Huang C-N, Cho YM, et al. Efficacy and safety of dulaglutide monotherapy compared with glimepiride in East-Asian patients with type 2 diabetes in a multicentre, double-blind, randomized, parallel-arm, active comparator, phase III trial. *Diabetes, obesity & metabolism*. 2018 09 (Epub 2018 Jun 2018;20(9):2121-2130. doi:10.1111/dom.13340
33. Battelino T, Bergenstal RM, Rodriguez A, et al. Efficacy of once-weekly tirzepatide versus once-daily insulin degludec on glycaemic control measured by continuous glucose monitoring in adults with type 2 diabetes (SURPASS-3 CGM): a substudy of the randomised, open-label,

parallel-group, phase 3 SURPASS-3 trial. *The lancet Diabetes & endocrinology*. 2022 06 (Epub 2022 Apr 2022;10(6):407-417. doi:10.1016/s2213-8587(22)00077-8
34. Gastaldelli A, Cusi K, Fernández Landó L, Bray R, Brouwers B, Rodríguez Á. Effect of tirzepatide versus insulin degludec on liver fat content and abdominal adipose tissue in people with type 2 diabetes (SURPASS-3 MRI): a substudy of the randomised, open-label, parallel-group, phase 3 SURPASS-3 trial. *Lancet Diabetes Endocrinol*. Jun 2022;10(6):393-406. doi:10.1016/s2213-8587(22)00070-5
35. Wilson JM, Nikooienejad A, Robins DA, et al. The dual glucose-dependent insulinotropic peptide and glucagon-like peptide-1 receptor agonist, tirzepatide, improves lipoprotein biomarkers associated with insulin resistance and cardiovascular risk in patients with type 2 diabetes. *Diabetes Obes Metab*. Dec 2020;22(12):2451-2459. doi:10.1111/dom.14174
